# Supplementary material for: Fatty acid metabolism controls plasma membrane cholesterol accessibility via ATGL-dependent phospholipid remodeling
Source: Nat Commun. 2026 Oct 14;17:11622. doi: 10.1038/s41467-026-78470-4 (PMC13755437; doi:10.1038/s41467-026-78470-4)
Supplement: Supplementary file 2 [file 41467_2026_78470_MOESM2_ESM.pdf]

nature portfolio

Corresponding author(s): Maia Kinnebrew

Last updated by author(s): September 10, 2026

Reporting Summary

Nature Portfolio wishes to improve the reproducibility of the work that we publish. This form provides structure for consistency and transparency in reporting. For further information on Nature Portfolio policies, see our Editorial Policies and the Editorial Policy Checklist.

Please do not complete any field with "not applicable" or n/a. Refer to the help text for what text to use if an item is not relevant to your study.
For final submission: please carefully check your responses for accuracy; you will not be able to make changes later.

Statistics

For all statistical analyses, confirm that the following items are present in the figure legend, table legend, main text, or Methods section.

n/a	Confirmed	
☐	☒	The exact sample size (n) for each experimental group/condition, given as a discrete number and unit of measurement
☐	☒	A statement on whether measurements were taken from distinct samples or whether the same sample was measured repeatedly
☐	☒	The statistical test(s) used AND whether they are one- or two-sided<br>*Only common tests should be described solely by name; describe more complex techniques in the Methods section.*
☒	☐	A description of all covariates tested
☐	☒	A description of any assumptions or corrections, such as tests of normality and adjustment for multiple comparisons
☐	☒	A full description of the statistical parameters including central tendency (e.g. means) or other basic estimates (e.g. regression coefficient) AND variation (e.g. standard deviation) or associated estimates of uncertainty (e.g. confidence intervals)
☐	☒	For null hypothesis testing, the test statistic (e.g. F, t, r) with confidence intervals, effect sizes, degrees of freedom and P value noted<br>*Give P values as exact values whenever suitable.*
☒	☐	For Bayesian analysis, information on the choice of priors and Markov chain Monte Carlo settings
☒	☐	For hierarchical and complex designs, identification of the appropriate level for tests and full reporting of outcomes
☒	☐	Estimates of effect sizes (e.g. Cohen's d, Pearson's r), indicating how they were calculated

Our web collection on statistics for biologists contains articles on many of the points above.

Software and code

Policy information about availability of computer code

Data collection	Flow cytometry data was gathered with the BD CSampler Software (for BD Accuri C6 measurements), or Cell Sorter Software (Sony), or NovoExpress Software (NovoCyte Penteon). Microscopy data was collected using Keyence BZ-X Series Control and Analyzer Software (Keyence Fluorescence Microscope) or with LasX software (Leica confocal). Quantitative PCR data was collected with the QuantStudio 5 Real-time PCR software. Western blots data used film that was scanned on a Epson Perfection V850 Pro scanner. Esterified and free cholesterol levels were measured with a Synergy H1 hybrid plate reader using Gen5 .209 software.
Data analysis	Flow cytometry analysis was completed with the FCS Flow software and microscopy data was analyzed with ImageJ. Mass spectrometry data was analyzed with SciexOS 3.4 software and RNA sequencing data was analyzed with Partek Flow.

For manuscripts utilizing custom algorithms or software that are central to the research but not yet described in published literature, software must be made available to editors and reviewers. We strongly encourage code deposition in a community repository (e.g. GitHub). See the Nature Portfolio guidelines for submitting code & software for further information.

Data

Policy information about availability of data

All manuscripts must include a data availability statement. This statement should provide the following information, where applicable:

- Accession codes, unique identifiers, or web links for publicly available datasets
- A description of any restrictions on data availability
- For clinical datasets or third party data, please ensure that the statement adheres to our policy

Genetic screen data is at NCBI Sequence Read Archive project ID: PRJNA1266240. Metabolomics is at Metabolomics Workbench with project ID: mkinnebrew_20250523_144048, RNA sequencing data is at NCBI GEO with ID: GSE298034. Source data has been provided as Source Data Files. All other data supporting the findings of this study are available from the corresponding author upon request.

Research involving human participants, their data, or biological material

Policy information about studies with human participants or human data. See also policy information about sex, gender (identity/presentation), and sexual orientation and race, ethnicity and racism.

Reporting on sex and gender	N/A
Reporting on race, ethnicity, or other socially relevant groupings	N/A
Population characteristics	N/A
Recruitment	N/A
Ethics oversight	N/A

Note that full information on the approval of the study protocol must also be provided in the manuscript.

Field-specific reporting

Please select the one below that is the best fit for your research. If you are not sure, read the appropriate sections before making your selection.

☑ Life sciences ☐ Behavioural & social sciences ☐ Ecological, evolutionary & environmental sciences

For a reference copy of the document with all sections, see nature.com/documents/nr-reporting-summary-flat.pdf

Life sciences study design

All studies must disclose on these points even when the disclosure is negative.

Sample size	No statistical method was used to predetermine sample sizes, but all in vitro cellular assays were performed in replicate (see replication below), and in vivo studies had sample sizes similar to those reported in previous publications:PMID: 28768177, PMID: 11283375, PMID: 17923673.
Data exclusions	No data exclusions were used in this study.
Replication	To ensure the reproducibility of our findings, all experiments were performed with at least three biological replicates. Mass spectrometry experiments used 4 biological replicates. With the exception of mass spectrometry and RNA sequencing experiments, all experiments were performed at least 2-3 times on independent days.
Randomization	Randomization was not used in this study.
Blinding	The investigators were not blinded during experiments or during outcome assessment. Blinding was not possible for the in vitroand in vivo studies as these experiments were performed by individual investigators who were aware of the experimental groups.

Behavioural & social sciences study design

All studies must disclose on these points even when the disclosure is negative.

Study description	N/A
Research sample	N/A
Sampling strategy	N/A
Data collection	N/A
Timing	N/A
Data exclusions	N/A
Non-participation	N/A
Randomization	N/A

Ecological, evolutionary & environmental sciences study design

All studies must disclose on these points even when the disclosure is negative.

Study description	N/A
Research sample	N/A
Sampling strategy	N/A
Data collection	N/A
Timing and spatial scale	N/A
Data exclusions	N/A
Reproducibility	N/A
Randomization	N/A
Blinding	N/A

Did the study involve field work? ☐ Yes ☑ No

Field work, collection and transport

Field conditions	N/A
Location	N/A
Access & import/export	N/A
Disturbance	N/A

Reporting for specific materials, systems and methods

We require information from authors about some types of materials, experimental systems and methods used in many studies. Here, indicate whether each material, system or method listed is relevant to your study. If you are not sure if a list item applies to your research, read the appropriate section before selecting a response.

Materials & experimental systems

n/a	Involved in the study	
☐	☒	Antibodies
☐	☒	Eukaryotic cell lines
☒	☐	Palaeontology and archaeology
☐	☒	Animals and other organisms
☒	☐	Clinical data
☒	☐	Dual use research of concern
☒	☐	Plants

Methods

n/a	Involved in the study	
☒	☐	ChIP-seq
☐	☒	Flow cytometry
☒	☐	MRI-based neuroimaging

Antibodies

Antibodies used	NPC1, RRID:AB_2152050; P38, RRID: AB_306166; SOAT1(ACAT1), RRID: AB_3750386; GAPDH, RRID:AB_2107436; ACC1 monoclonal, RRID:AB_2882621; ACC1 mouse polyclonal conjugated, RRID:AB_2934949; ACC1 phospho-S79, RRID:AB_11156104; ATGL, RRID:AB_2888664; LDLR, RRID:AB_2281164;LaminA/C, RRID:AB_2296961; GFP, RRID:AB_11182611; FASN, RRID:AB_2100801; Alpha TUBULIN, RRID:AB_2210206; Beta-ACTIN, RRID:AB_476744; ACC1 and ACC2 doi: 10.1016/j.cmet.2017.07.009; HRP-conjugated Affinipure Goat Anti-Rabbit IgG (H+L), RRID:AB_2722564; HRP-conjugated Affinipure Goat Anti-Mouse IgG (H+L), RRID:AB_2722565
Validation	All commercial antibody validation information is available at vendor websites. ACC1 and ACC2 antibodies used in mouse studies (Figure 7d) were validated in the publication: Cell Metab. 2017 Aug 1;26(2):394–406.e6. doi: 10.1016/j.cmet.2017.07.009

Eukaryotic cell lines

Policy information about cell lines and Sex and Gender in Research

Cell line source(s)	HAP1 haploid human cells were kindly provided by Thijn Brummelkamp, now available from Horizon Discovery, Cambridge, United Kingdom. 3T3 cells were purchased from Thermo Fisher Scientific Catalogue # R76107. 293T cells were purchased from ATCC, Catalogue # CRL-3216; NPC1 knockout MEFs were kindly provided by Tim Stearns.
Authentication	All purchased cell lines came with a certificate of authentification from the vendor and were used without further validation. CRISPR knockout cell lines generated in this manuscript were shown to be knockouts using a Western blot whenever a high quality antibody could be found; otherwise, gene knockouts were confirmed by PCR analysis.
Mycoplasma contamination	All cell lines tested negative for mycoplasma.
Commonly misidentified lines (See ICLAC register)	No commonly misidentified cell lines were used

Palaeontology and Archaeology

Specimen provenance	N/A
Specimen deposition	N/A
Dating methods	N/A

☐ Tick this box to confirm that the raw and calibrated dates are available in the paper or in Supplementary Information.

Ethics oversight	N/A

Note that full information on the approval of the study protocol must also be provided in the manuscript.

Animals and other research organisms

Policy information about studies involving animals; ARRIVE guidelines recommended for reporting animal research, and Sex and Gender in Research

Laboratory animals	ACC1 floxed alleles were generated by inserting loxP sites between exons 26-27 and between exon 21-22. Acc2f/f mice were purchased from Jackson labs. ACC1 and ACC2 liver-specific double knockout mice was generated by crossing with albumin-CRE transgenic mice. This was previously reported in detail in PMID: 28768177.
Wild animals	No wild animals were used in this study.
Reporting on sex	All mice used in this study were males, which is reported in our methods.
Field-collected samples	This study did not involve samples collected from the field.
Ethics oversight	All animal experiments were performed with approval from the Institutional Animal Care and Use Committee (IACUC).

Note that full information on the approval of the study protocol must also be provided in the manuscript.

Clinical data

Policy information about clinical studies

All manuscripts should comply with the ICMJE guidelines for publication of clinical research and a completed CONSORT checklist must be included with all submissions.

Clinical trial registration	N/A
Study protocol	N/A
Data collection	N/A
Outcomes	N/A

Dual use research of concern

Policy information about dual use research of concern

Hazards

Could the accidental, deliberate or reckless misuse of agents or technologies generated in the work, or the application of information presented in the manuscript, pose a threat to:

No	Yes	
☒	☐	Public health
☒	☐	National security
☒	☐	Crops and/or livestock
☒	☐	Ecosystems
☒	☐	Any other significant area

Experiments of concern

Does the work involve any of these experiments of concern:

No	Yes	
☒	☐	Demonstrate how to render a vaccine ineffective
☒	☐	Confer resistance to therapeutically useful antibiotics or antiviral agents
☒	☐	Enhance the virulence of a pathogen or render a nonpathogen virulent
☒	☐	Increase transmissibility of a pathogen
☒	☐	Alter the host range of a pathogen
☒	☐	Enable evasion of diagnostic/detection modalities
☒	☐	Enable the weaponization of a biological agent or toxin
☒	☐	Any other potentially harmful combination of experiments and agents

Plants

Seed stocks	N/A
Novel plant genotypes	N/A
Authentication	N/A

ChIP-seq

Data deposition

☐ Confirm that both raw and final processed data have been deposited in a public database such as GEO.

☐ Confirm that you have deposited or provided access to graph files (e.g. BED files) for the called peaks.

Data access links<br>*May remain private before publication.*	N/A
Files in database submission	N/A
Genome browser session<br>(e.g. UCSC)	N/A

Methodology

Replicates	N/A
Sequencing depth	N/A
Antibodies	N/A
Peak calling parameters	N/A
Data quality	N/A

Software |
---|---

Flow Cytometry

Plots

Confirm that:

☒ The axis labels state the marker and fluorochrome used (e.g. CD4-FITC).

☐ The axis scales are clearly visible. Include numbers along axes only for bottom left plot of group (a 'group' is an analysis of identical markers).

☐ All plots are contour plots with outliers or pseudocolor plots.

☒ A numerical value for number of cells or percentage (with statistics) is provided.

Methodology

Sample preparation	Adherent cells were dissociated with trypsin (Gibco). Cells were then resuspended into a single-cell population by mechanical separation (pipetting up and down). Cells were then concentrated with centrifugation, and the cell pellet was resuspended with 25 uL of ice cold 2 uM purified mNeonGreen-ALOD4 protein diluted into filtered 1x PBS and 1% BSA. Cells were then incubated on ice for 15 minutes. Next, the cell mixture was diluted to about 200 uL in ice cold filtered 1x PBS and 1% BSA. Finally, cells were analyzed for FITC signal on a live and single-cell gated population.
Instrument	Flow cytometry analysis was performed on either a BD Accuri C6 Pro flow cytometer, a Sony Cell Sorter or on the NovoCyte Penteon. The choice of which analyzer was based on their availability. The NovoCyte Penteon was used for all Laurdan measurements.
Software	All flow cytometry analysis was carried out on the FCS Flow software.
Cell population abundance	All analyses were carried out on the majority population of cells, which was the single-cell and live-cell population. This population always represented at least 60% of all cells. HAP1 cells are prone to sticking together (doublet cells), which is what limited this from being a higher percentage.
Gating strategy	Cells were first gated for single-cells and live cells using a density plot of the forward scatter-height versus the forward scatter-area. Next, cells were gated based on their granularity (to remove dying or stressed cells) by generating a density plot of forward scatter-area versus side scatter-area.

☒ Tick this box to confirm that a figure exemplifying the gating strategy is provided in the Supplementary Information.

Magnetic resonance imaging

Experimental design

Design type	N/A
Design specifications	N/A
Behavioral performance measures	N/A

Imaging type(s)	N/A
Field strength	N/A
Sequence & imaging parameters	N/A
Area of acquisition	N/A

Diffusion MRI ☐ Used ☒ Not used

Preprocessing

Preprocessing software	N/A
Normalization	N/A
Normalization template	N/A
Noise and artifact removal	N/A
Volume censoring	N/A

Statistical modeling & inference

Model type and settings	N/A
Effect(s) tested	N/A

Specify type of analysis: ☐ Whole brain ☐ ROI-based ☐ Both

Statistic type for inference
(See Eklund et al. 2016)

N/A

Correction

N/A

Models & analysis

n/a	Involved in the study
☒	☐ Functional and/or effective connectivity
☒	☐ Graph analysis
☒	☐ Multivariate modeling or predictive analysis

Functional and/or effective connectivity

N/A

Graph analysis

N/A

Multivariate modeling and predictive analysis

N/A

This checklist template is licensed under a Creative Commons Attribution 4.0 International License, which permits use, sharing, adaptation, distribution and reproduction in any medium or format, as long as you give appropriate credit to the original author(s) and the source, provide a link to the Creative Commons license, and indicate if changes were made. The images or other third party material in this article are included in the article's Creative Commons license, unless indicated otherwise in a credit line to the material. If material is not included in the article's Creative Commons license and your intended use is not permitted by statutory regulation or exceeds the permitted use, you will need to obtain permission directly from the copyright holder. To view a copy of this license, visit http://creativecommons.org/licenses/by/4.0/
