# Supplementary material for: Fatty acid metabolism controls plasma membrane cholesterol accessibility via ATGL-dependent phospholipid remodeling
Source: Nat Commun. 2026 Oct 14;17:11622. doi: 10.1038/s41467-026-78470-4 (PMC13755437; doi:10.1038/s41467-026-78470-4)
Supplement: Supplementary file 3 [file 41467_2026_78470_MOESM3_ESM.pdf]

nature portfolio

Peer Review File

Fatty acid metabolism controls plasma membrane cholesterol accessibility via ATGL-dependent phospholipid remodeling

Corresponding Author: Dr Maia Kinnebrew

This file contains all reviewer reports in order by version, followed by all author rebuttals in order by version.

Version 0:

Reviewer comments:

Reviewer #1

(Remarks to the Author)
This manuscript claims to identify ACC1 as a specific regulator of plasma membrane cholesterol through lipid droplet-mediated trafficking. However, careful examination reveals that the authors are documenting non-specific metabolic collapse that affects all cellular lipids, not a cholesterol-specific regulatory mechanism.
Cholesterol flips between membrane leaflets in microseconds (PMID: 30633868, 34781553). It equilibrates across cellular membranes in seconds to minutes. But ACC1 inhibition takes 2-6 hours to change PM cholesterol levels. That's more than a 1000-fold difference. When you see changes that slow, you're not looking at cholesterol transport anymore - you're watching the entire cellular lipid metabolism fall apart and reorganize.
Think about what happens when you inhibit ACC1: fatty acid synthesis stops immediately, lipid droplets start breaking down through ATGL, fatty acyl-CoA pools crash, membrane phospholipid composition shifts. By hour 6, these aren't normal membranes anymore. The cholesterol changes are just one piece of this larger metabolic disruption.
The RNA-seq data actually support this interpretation (Fig. 6g-h). Both SREBP1 (fatty acid genes) and SREBP2 (cholesterol genes) pathways activate simultaneously. If this were specifically about cholesterol, you'd only see SREBP2 activation. Instead, the cells are mounting a panic response to global lipid depletion. The membrane blebbing in the hepatocytes (Fig. 6e) further suggests these membranes are fundamentally compromised.
Another telling sign: the phenotype is irreversible. ACC1 knockout cells maintain elevated PM cholesterol for weeks despite having functional cholesterol export machinery. That's not how regulated processes work - they're reversible. Once you've broken lipid metabolism this badly, the cells enter a new steady state with altered membrane properties that happen to accommodate more cholesterol.
The oleic acid rescue experiments need much better controls. Oleic acid doesn't just restore lipid droplets - it inhibits ATGL (PMID: 23297223), triggers autophagy pathways (PMID: 25586377), changes membrane fluidity because it's unsaturated, and activates various stress responses. When the authors see partial cholesterol "rescue" with oleic acid, which of these effects is responsible? They don't know. They need to test saturated fatty acids (do they also work?), use specific ATGL inhibitors (does blocking catabolism alone help?), and check whether the rescue correlates with normalized membrane composition rather than just LD numbers.
The correlation between lipid droplet abundance and PM cholesterol is real and interesting, but the causation isn't what the authors think. They're proposing that LDs directly buffer PM cholesterol. More likely, LD loss is a marker of the metabolic catastrophe that indirectly affects cholesterol through wholesale changes in membrane composition. When you lose LDs, you're not just losing cholesterol storage sites - you're documenting the collapse of normal lipid metabolism.
If the authors want to salvage this, they need to:
1. Do a proper lipidomics time course. Show us what happens to all lipids, not just cholesterol.
2. Test whether maintaining normal membrane composition (not just adding oleic acid) prevents the cholesterol changes.
3. Include proper controls for oleic acid's many effects.
4. Consider reframing this as a study of metabolic disruption rather than specific cholesterol regulation.
There's clearly something interesting happening with ACC1 and cellular lipids. The connection to lipid droplets is real. But calling this specific cholesterol regulation misrepresents the biology. The authors are seeing the cholesterol component of a much larger metabolic phenomenon. That's still worth publishing, but it needs to be described accurately.
The therapeutic implications they mention for NPC disease are premature given that they're inducing broad metabolic dysfunction. You can't fix a specific trafficking defect by breaking the entire system in a different way. The cells might have

similar cholesterol levels, but for completely different reasons.
I'd encourage the authors to embrace what they've actually discovered - that ACC1 inhibition causes a cascade of metabolic changes affecting all cellular lipids. That's interesting biology. Forcing it into a "specific cholesterol regulation" story does a disservice to their own data.

Reviewer #2

(Remarks to the Author)
Wijesinghe et al identified ACC1 as a regulator of cholesterol transport from the plasma membrane in a genome-wide screen. Knockdown of ACC1, which is the rate-limiting gene in fatty acid synthesis, unexpectedly reduces cholesterol trafficking, but they find that increasing lipid droplet mass by oleic acid loading or by ATGL knockout restores cholesterol trafficking. They provide evidence that ACC1's effect on cholesterol trafficking is independent of ACAT1/2, NPC1 and new synthesis. The critical assay in this work is to measuring binding of mNeonGreen-ALOD4, which I think is reliable under the conditions used. The effects measured are quite large and seem statistically robust.

They test their hypothesis by manipulating proteins that directly affect lipid droplet volume. Knockout of ACC1 under serum starvation reduces LD number. They claim that knockout of DGAT1 and DGAT2 failed to eliminate lipid droplets, but these were under 5% serum rather than starvation conditions. They show that ATGL knockout (which reduces catabolism of stored lipid in lipid droplets) allows normal trafficking of cholesterol even with ACC1 knockout, consistent with their hypothesis. It would be useful to test ATGL overexpression (perhaps tagged with mCherry), which can considerably reduce lipid droplet mass, at least in some cell lines. In their model, ATGL overexpression (provided it reduced lipid droplet mass) would be expected to reduce cholesterol trafficking even in the presence of normal levels of ACC1.

Overall, this is an interesting paper with good quality data. Further work would be needed to achieve a complete mechanistic understanding of what's going on, but I think this would be of use to the cell biology community.

Reviewer #3

(Remarks to the Author)
The manuscript reports identification of plasma membrane (PM) cholesterol regulation pathway involving ACC1 and lipid droplets (LDs). The work potentially highlights a novel functional role of an essential organelle (LDs) and is thus likely to stimulate broad interest.

However, in its present form, the proposed mechanistic links between ACC1 and PM cholesterol regulation remain not fully substantiated, lacking direct evidence / molecular mechanism, as well as datasets that can unambiguously rule out alternative mechanisms. The following queries should thus be addressed in full, in backing up the authors' claims and to strengthen the key conclusions.

We further request the authors to doubly reassure the novelty statements made in the manuscript, and tone down or clarify such claims as necessary. For instance, Abstract makes novelty claims about what appear to be knowledge already available in the literature, such as: "Mechanistically, we find that ACC1 regulates lipid droplet catabolism…." The fact that ACC1 regulates LDs is established in the field (via AMPK1 kinase phosphorylation suppressing fatty acid synthesis that otherwise supports LD anabolism), meaning also that several of the datasets in main fig. 5 do not represent new knowledge. Thus, statements as such throughout the manuscript should be tightened up and carefully proof-checked.

Major points:

(1) Writing / accessibility

The manuscript is currently written for the readership of field-specialised journals such as Journal of Lipid Research– thus issues below do need major overhaul such that the paper can be made more accessible for broader readership that could be ultimately suited to journals such as Nature Communications. Just to outline a few issues – they are not exhaustive however:

(a) Introduction has a lot of gene acronyms largely without briefly mentioning to the reader what they are / their key roles. Currently too much "jargons" as such is summarised in 3 short paragraphs in the intro, and for almost each abbreviation, readers are forced to look up online what these individual genes/proteins do - at least >10-15 proteins, without spelling out abbreviations (except for CH25H), are all squashed into a single page intro. This issue continues – for instance, what is NPC1, what is LXR, what are their known roles and organelle-location etc., in the context of this paper (?)

(b) It would greatly assist the reader f what is known vs. unknown or unclear, etc. can be presented as a supplementary figure that can clearly show where these various genes lie in the known cholesterol trafficking pathways and associated LD catabolism / anabolism. I see that main fig 1e has an illustration, but it does not cover all the key genes mentioned in the intro several of which are manipulated (inhibited, knocked out, or generally studied further in this work) in order to derive key conclusions – thus they must be integrated in an illustrative figure, which can be in SI, that provides a more comprehensive and accessible information (than fig 1e)

(c) Order of the text and logic is also largely off. For instance, mNeonGreen explanation flies in only in line 115 (Fig 1F) although this assay was used right from the beginning– e.g., Fig. 1A and S1A (line 95 onwards, the paragraph above).

Readers are left to look up everything as they progress through every sentence of this paper… Likewise, NCP1 was explained finally on line 139, when its inhibition was executed/discussed in 2 pages above

(2) Consistency and rigor

(a) Most of the conclusions thus far are derived from gene deficiency test, often through the use of multiple inhibitors. In the case of ATGL, it is clear as discussed in lines 324-328 that there are phenotypic differences between CRISPR KO and inhibitor treatment. That being said, given that many inhibitors are pleotropic / poly-pharmacologic (including not-yet-known-targets etc.), in experiments where inhibitor usage alone has been solely deployed, the corresponding conclusion should be backed up by parallel KO/KD experiments. This includes – but not limited to – AMPK, ACSL, and FASN. [AMPK is key to support the claimed new knowledge (if any) in the context of AMPK / ACC1 (ACC2) – fatty acid – LD regulatory cascade, and the latter two directly affect the level of LDs, especially ACSL inhibition could give similar outcomes as ACC1 KO or inhibition].

(b) The authors must do due diligence to rule out potential off-targets in deploying KO/KD. Multiple guide-RNAs (or si/shRNAs where applicable), should have been used in each case and consistently across the board. Please thus remedy these issues in all cases in the present study, to improve rigor in experimental design, and mitigate any possibility of wrong conclusions caused by unintended off-target effects.

(c) Statistical analysis needs improved rigor as well. Currently several datasets appear to have been over-interpreted. For instance, in datasets with two experimental variables, two-way ANOVA [followed by appropriate corrected t-test (where applicable)] is a go-to statistical treatment approach (as opposed to one-way ANOVA or t-test with Welch corrections that the authors used). Just to point out one-of-many examples, bar 2 and 4 in Fig.1J – likely are different, but the conclusion is that ACC1 inhibition does not raise PM Cholesterol levels through a mechanism involving NPC1 (?). We urge the authors to please redouble their efforts in re-checking / correcting all the statistical analyses and data interpretations / conclusions made.

(d) Related to point (c) above, the choice of statistical treatment overall seems rather arbitrary, non-standard, or occasionally, invalid. Decision tree for making such calls (i.e., the choice of stat test and why), should be provided, after revisiting all the analyses / correcting the types of treatment, and where relevant, standardizing the choice of tests used.

(3) On the proposed mechanism: ACC1/ACSL Inhibition Drives LD Catabolism via ATGL

(a) The manuscript claims that the deficiency in ACC1 induces LD catabolism via ATGL, with key data in Figure 4e and 4f. The mechanism via which ACC1 inhibits ATGL remains highly speculative (based on the data presented thus far, this is likely an overstatement). First, as it stands, the paper has no direct evidence to show for instance inhibitory interaction between ACC1 and ATGL. Regulation of LD levels involves multiple pathways and proteins, rendering the claimed link between ACC1 from fatty-acid synthesis and ATGL in TAG degradation, remains unsubstantiated. Direct experimental validations with orthogonal approaches are necessary to sure up this proposed link, as well as to provide a clear underlying molecular mechanism.

(b) LD amount/levels affecting PM cholesterol is convincing based on the data presented thus far. Hence, in the absence of substantial direct inhibition mechanism that supports the authors' model, one possibility is that LD only plays a cargo role to transport cholesterol from PM. Indeed, there are numerous other sources/pathways that lead to fatty acid accumulation that enables LD formation even when ACC1 is deficient; and active ATGL could serve to suppress the LDs because ACC1-dependent primary flux is inhibited, but inhibited ATGL does not have such an ability. The authors should thus also rule out additional possibilities that could result in the same phenotypic changes.

(c) Addressing the direct molecular mechanisms (mentioned in (a) above) is absolutely crucial before this work can be published. Since LD regulation is of multifaceted network with manifold interdependent players and feedforward/feedback loops (notwithstanding numerous yet-unknowns), only relying solely upon KO/KD and inhibitor treatments may not give us the full truth. To that end, using cleaner biochemical assays involving purified enzymes beyond as cell-based experiments, could greatly assist the authors, and eventual impact and validity of this otherwise very interesting work.

(d) Could oleic acid (OA) addition rescue the amount of LD in ATGL depleted or inhibited background, while also assessing the amount of the PM cholesterol?

(e) ACSL should also be investigated further since ACSL knockdown/inhibition showed similar results to ACC1 deficiency. The authors discussed the crosstalk between ACSL and ATGL in Figure 5a-c, which show that the LD amount is directly related to the PM cholesterol level. This somewhat diminishes the importance and uniqueness of ACC1. It could be argued, based on the presented data, that the processes of fatty acid activation or TAG synthesis may play a more central role instead of ACC1. ATGL and ACSL KO should also be assessed, as generically mentioned above.

(f) Figure 4d and Figure 5b,g show that LD sizes and LD counts are altered in two ATGL-deficient cases. We wonder if there is a way to calculate and discuss the entire LD content level, such as area x numbers (by imaging, or other flow-based or biochemical assays). Such an analysis measuring the total amount of lipids, the total amount of cholesterol, and CEs would provide a more holistic view on phenotypic changes. It is also quite challenging / impossible to discern from the presented data / figure legends, the corresponding sample size / particle counts made (e.g., no. of LDs quantified per cell in each set; no. cell quantified for cholesterol content in each group, etc.)

Other points:

(i) Flow cytometry data – raw data and gating analysis should be shown – these are standards in many journals including this

(ii) likewise, all blots/gels should be standardly shown in full-view uncropped format as supplementary information.

(iii) The authors performed TKO, DKO, etc. The supplementary information description for these procedures is insufficient / lacking details.

(iv) Line 128 - Is ACC1 activity known for the truncated version?

(v) Line 187 - The differences in data Fig. 2f and Fig. 1k should be compared in one single plot and with adjusted comparison.

(vi) Figure 2n - The first bar (WT basal) and the third bar (ACC1 KO basal) should be compared. (As requested above, please use the correct type of statistical treatment)

(vii) Line 225 - The more common format is BODIPY 493/503

(viii) As described, CRISPR KO of ACC1 at various exons cause multiple phenotypes: please specify the specific exons being targeted when discussing the ACC1 KO datasets in subsequent paragraphs.

(ix) In lines 266 – 267, ….stimulates LD catabolism by starving the cell of fatty acyl-CoA. I've not been able to find the evidence in the manuscript or references.

Reviewer #4

(Remarks to the Author)
I co-reviewed this manuscript with one of the reviewers who provided the listed reports. This is part of the Nature Communications initiative to facilitate training in peer review and to provide appropriate recognition for Early Career Researchers who co-review manuscripts.

Version 1:

Reviewer comments:

Reviewer #1

(Remarks to the Author)
The authors have done substantial work in response to my first round, and I want to acknowledge that. The TAG-TKO cell line is elegant, the lipidomics is thorough, and abandoning the LD-buffering model when their data didn't support it reflects scientific integrity I respect. The new mechanistic spine - fatty acyl-CoA depletion activates ATGL, which remodels phospholipid saturation, which alters PM cholesterol accessibility - is more coherent than what they had before.
That said, they've replaced one overreaching story with another. The new manuscript leans on a mechanism the authors themselves admit they can't explain, frames a generalizable biophysical principle as if it were specifically about ACC1, contains a paradox they handwave away, and silently dropped negative data that should have stayed in.

Major concerns:
The central mechanistic claim is unsupported by direct evidence. The headline finding of this revision is that ATGL remodels DAGs toward polyunsaturated species independently of TAG hydrolysis. Everything downstream depends on this. And in their discussion, the authors write that "future work will be needed to determine how ATGL remodels the cellular DAG pool." That sentence is a confession. You don't get to defer the central claim of your paper to future work. The simplest experiment that would resolve this is a catalytic-dead ATGL rescue in the ATGL-KO + ACC1i background. If catalytically inactive ATGL restores PC/PE unsaturation, the activity is allosteric or scaffolding; if it doesn't, ATGL's catalysis is required, and the question becomes against what substrate, given that there is essentially no TAG in TAG-TKO cells. The transacylase hypothesis is suggestive but isn't evidence in this system. One clean experiment would settle it.

ACC1 is no longer the protagonist of this story. ACSL inhibition phenocopies ACC1 inhibition. Palmitic acid, which doesn't require ACC1, rescues it. FASN-KO does the same thing given enough time. The biologically relevant variable is fatty acyl-CoA availability, and ACC1 is one of several enzymes whose inhibition depletes it. That's a fine finding, but it isn't an "ACC1 inhibits ATGL" finding, which is what the title still says. I anticipate the response will be that ACC1 was the top screen hit, that Firsocostat is in clinical development, and that the dLKO model is ACC1/ACC2-specific, which are all true. But the screen and the translational angle don't change the fact that the mechanism, as the authors describe it, has nothing specifically to do with ACC1. The honest framing is that the screen identified ACC1 as a tractable entry point into a general fatty acyl-CoA/ATGL axis. The title and abstract should reflect the mechanism, not the screen hit.

The "trapped accessible cholesterol" model conflates two different observables. The authors argue that ATGL activation

increases ALOD4 binding while decreasing MβCD extractability, and they call this a paradox resolved by an energetic argument about acyl chain shielding. It isn't a paradox. ALOD4 reports cholesterol's chemical activity at the membrane surface - hydroxyl exposure, recognition by a protein with cooperative kinetics. MβCD extraction reports the rate of monomer dissociation into a hydrophobic acceptor. These are not the same physical property and were never required to track linearly. The data may show something simpler and more interesting: in fluid, polyunsaturated membranes, cholesterol presents differently to a protein probe than to a small-molecule acceptor. That's worth reporting, but it requires the authors to define what they mean by "accessible cholesterol" - the operational definition (binds ALOD4) or the functional one (moves out of the membrane). They cannot have both, and this discordance should be the headline of Figure 7, not buried as a paradox to be waved off.

The AMPK data was silently removed. The original submission contained AMPK data implicating AMPK in the regulatory axis. The rebuttal explains it's gone - AMPK1/2-DKO lethal, AMPK2-KO no effect, AMPK1 siRNA ambiguous. But the manuscript contains no trace. Negative results that rule out a plausible mechanism belong in the manuscript, not in a rebuttal letter readers won't see. Add a supplementary figure and a sentence in discussion. Without this, removal of data that was in the original submission looks like selective reporting, even if it isn't.

The actual contribution is being oversold. The principle that PUFA-rich membranes disrupt cholesterol-acyl chain packing is well established in vitro. What's novel here is how this principle is regulated in living cells, by which enzymes, on what timescale. The discussion frames the finding as a discovery about ACC1 and ATGL specifically and obscures the broader biophysical principle the authors have actually demonstrated. A more accurate framing would strengthen the paper, not weaken it.

Minor points:
The Kennedy pathway connection is supported by acyl chain signature matching but not by direct flux measurement; isotopic labeling would demonstrate it and would also strengthen the response to point 1.

The new mechanistic findings are essentially HAP1-only - repeating Laurdan and DAG measurements in one additional cell line would help.

Several lipid classes (PG, plasmalogens, LPE) change with ACC1 inhibition but don't reverse with ATGL inhibition, meaning ACC1 inhibition has substantial ATGL-independent lipidomic effects that receive no discussion; this should be acknowledged honestly rather than dismissed.

In conclusion, the science here is good. The TAG-TKO is clever, the lipidomics resolves much of what I challenged in round one, and the mouse data is appropriate. My objections are mostly about framing, mechanistic completeness on the central new claim, and transparency around AMPK. These are addressable. With a catalytic-dead ATGL experiment, an honest reframing toward phospholipid saturation as the operative mechanism, and restoration of the AMPK negative results, this becomes a strong paper.

Reviewer #2

(Remarks to the Author)
While this is a highly complex system, I believe that the authors have addressed the major concerns adequately and this work will be of general interest to cell biologists.

Reviewer #3

(Remarks to the Author)
The authors have made commendable efforts during revisions. The rewriting of the introduction and inclusion of supporting illustrations have made this revised paper much more accessible to broader mechanistic life scientists as well, including those not specialised in lipid maintenance pathways exclusively.

A few additional questions / comments to be addressed are below:

- what is the rational behind the choice of Sidak correction post one-way ANOVA, as Dunnett is used commonly, at least for many-to-one comparisons - the authors should fix the statement and corresponding plots / re-analyze where applicable. One can also consider Tukey for all possible multiple pairwise comparisons. It is unclear if Sidak should be the go-to for every dataset in this paper following suitable application of one-way ANOVA.

- one-way ANOVA classically assumes equal level of spread so statement 'one-way ANOVA' 'does not require equal variances' is limited and requires update/correction.

- the authors defended in several instances how ">750 or so publications" have used several of these compounds as inhibitors, implying that usage of these compounds could be warranted or should not be queried. Sadly no end of previously reported publications can rule out off-targets or guarantees specificity if these published studies had not carried out responsible orthogonal validations in identical biological contexts (cell lines, etc.) as in this paper - I would think that the authors are just as aware of numerous cases of misconceptions, incorrect pathway models, mechanisms, conclusions, etc., perpetuated over decades in many fields of life sciences. Thus, although I congratulate the authors for improving the rigor

and responsibility in numerous ways through the revisions, I would point out that defending with no. of previous publications dilutes the focus when the soultion exclusively rests on what the authors indeed found in their own orthogonal assay results during revisions. Even clinically approved drugs hit multiple targets.

I believe that responses to the above comments/queries can be overseen by the handling Editor. I further think that the other reviewers have also asked many relevant and important (often subject-specific) concerns. Taken together, I am pleased to support the publication on two conditions:
-- providing these comments above be fully addressed and taken into consideration by the authors, as judged by the Editor, and
-- providing the other reviewers have also fully approved the next round(s) of the revised manuscript. I thus do not need to re-review from my side unless the Editors request otherwise.

I wish the authors good luck with their ongoing and future investigations in this area.

Reviewer #4

(Remarks to the Author)
I co-reviewed this manuscript with one of the reviewers who provided the listed reports. This is part of the Nature Communications initiative to facilitate training in peer review and to provide appropriate recognition for Early Career Researchers who co-review manuscripts.

Version 2:

Reviewer comments:

Reviewer #1

(Remarks to the Author)
The authors have significantly enhanced the manuscript's structure, clarity, and statistical rigour. The TAG TKO approach, lipidomics, and PM cholesterol measurements convincingly establish a regulated axis. This axis involves fatty acyl CoA depletion activating ATGL, remodelling PC/PE saturation, and altering plasma membrane cholesterol accessibility.
My primary concern remains the strength of the mechanistic language surrounding ATGL's triacylglycerol-independent DAG remodelling and transacylase activity. Since there's no catalytic dead ATGL rescue or equivalent experiment in this system, I suggest the authors explicitly frame these aspects as a model supported by prior literature and inhibitor data, rather than as a proven mechanism. This clarification should be made in the abstract and discussion, using phrases like "we propose" or "our data are consistent with the model that…" instead of categorical statements.
With this adjustment, I would be happy to recommend publication in Nature Communications.

Reviewer #2

(Remarks to the Author)
This paper has been extensively revised and now argues that cholesterol accessibility at the cell surface in cells is regulated in part by activation of ATGL with this effect preserved even if TAG formation is blocked. Evidence for this includes mass spec showing ATGL activation elevates DAGs which are precursors to membrane phospholipids. They argue that this depends on transacetylase activity by ATGL leading eventually a resulting increase in unsaturated fatty acids in membrane lipids. In their model, this results in an increase of cholesterol accessibility in the PM. Although this is a highly complex system and the dependence on transacetylase activity specifically may be difficult to definitively show in vivo, it is backed by citation of in vivo studies, and also lipidomics which seems to show an increase in polyunsaturated DAGS dependent on ATGL. Overall, the model of the authors is plausible and backed by considerable evidence in the current revision of the paper. My feeling is that while there are still some difficult unsolved questions the paper has been greatly improved and will be of interest to cell biologists and will also prove useful as a basis for future research.

Reviewer #3

(Remarks to the Author)
In the previous round of revisions, I have included the following final recommendation in my review

"I believe that responses to the above comments/queries can be overseen by the handling Editor. I further think that the other reviewers have also asked many relevant and important (often subject-specific) concerns. Taken together, I am pleased to support the publication on two conditions:
-- providing these comments above be fully addressed and taken into consideration by the authors, as judged by the Editor, and
-- providing the other reviewers have also fully approved the next round(s) of the revised manuscript. I thus do not need to re-review from my side unless the Editors request otherwise.

I wish the authors good luck with their ongoing and future investigations in this area."

Having gone through the latest version of the manuscript and the other review comments, I do realize that the other referees continue to have non-insignificant concerns. It would be helpful if the authors and the remaining referees could identify the middle ground. Below I provide my personal viewpoints and hope these are of help -

First, Nature Communications is a broader readership journal and thus new scientific discoveries of interest and relevance across multiple fields, providing the conclusions and experimental designs, data analyses/interpretations, etc., are rigorous and undertaken with holistic depth and scope should be supported for publications. In this spirit, this manuscript (after several rounds of revisions) do meet such requirements.

However, I must also point out that the goal post of the manuscript has shifted in the process of revisions and cutting down some of the initially reported datasets / claims that were not fully substantiated. It appears to me that other reviewers prefer to remain focused on the initial focus on the paper, leading to their limited satisfaction with the revised manuscript. This appears to be a significant stumbling block. I personally believe that the best way to bring this to amicable conclusion is via open communications between both sides mediated by the handling editor and for the authors to clearly cede the limitations of the paper while stressing the key additions brought along through revisions.

Reviewer #4

(Remarks to the Author)
I co-reviewed this manuscript with one of the reviewers who provided the listed reports. This is part of the Nature Communications initiative to facilitate training in peer review and to provide appropriate recognition for Early Career Researchers who co-review manuscripts.

Open Access This Peer Review File is licensed under a Creative Commons Attribution 4.0 International License, which permits use, sharing, adaptation, distribution and reproduction in any medium or format, as long as you give appropriate credit to the original author(s) and the source, provide a link to the Creative Commons license, and indicate if changes were made.
In cases where reviewers are anonymous, credit should be given to 'Anonymous Referee' and the source.
The images or other third party material in this Peer Review File are included in the article’s Creative Commons license, unless indicated otherwise in a credit line to the material. If material is not included in the article’s Creative Commons license and your intended use is not permitted by statutory regulation or exceeds the permitted use, you will need to obtain permission directly from the copyright holder.
To view a copy of this license, visit https://creativecommons.org/licenses/by/4.0/

Nature Communications manuscript

Dear Reviewers,

We sincerely thank you for the careful evaluation of our manuscript and for the constructive feedback, which has greatly contributed to improving the clarity of our work. We also appreciate the opportunity to submit a revised version for your consideration.

We carefully considered each of the points raised by the reviewers and provided a detailed response to each comment. Where possible, we have incorporated new experimental data and manuscript revisions. Reviewer comments are in blue italics, our responses are in black, and figure text call-outs are in magenta. All figure numbers refer to the revised manuscript. Updated manuscript text is highlighted in light blue.

While multiple reviewers appreciated the lipid droplet (LD) connection in our work, we sought to rigorously test the model that plasma membrane (PM) accessible cholesterol is modulated by LD abundance with an orthogonal method that did not rely on modulating ACC1 or ATGL activity. This analysis has led us to a new model, which is communicated in two entirely new figures (**Figure 5** and **6**). We believe the manuscript is now stronger, more interesting, clarifies the underlying mechanism, and will be of broader reaching importance. We outline the new key results here for your convenience, with further details added in the revised text and in response to specific reviewer comments.

Major new findings:

1. **ACC1 indirectly inhibits ATGL by contributing to the synthesis of long-chain fatty acyl-CoAs (revised Figures 3 and 4)**. We previously overlooked a seminal paper showing that long-chain fatty acyl-CoAs allosterically inhibit ATGL (PMID: 24440819). In light of this paper, it is highly logical that ACC1 impacts ATGL activity given ACC1's function as the rate-limiting enzyme in fatty acid biosynthesis. We propose that ACC1 inhibition stimulates ATGL activation by depleting cellular fatty acyl-CoA pools. Key data supporting this conclusion:
    a. Inhibition or knockout of the ACSL proteins, which charge fatty acids with coenzyme A, have the same effect as ACC1 loss. This suggests that fatty acyl-CoAs (the common metabolic product of ACC1 and ACSLs) are important for PM cholesterol regulation. It also shows that the effects we observe are not specific to ACC1.
    b. The effects of ACC1 loss are reversed by exogenous oleic or palmitic acid addition; ACSL loss, on the other hand, is not reversed by fatty acid addition because ACSLs are required to convert fatty acids into fatty acyl-CoAs. This demonstrates that fatty acyl-CoA abundance is tied to PM cholesterol changes.
    c. Inhibition or knockout of ATGL substantially attenuates the effects of ACC1 and ACSL loss on PM cholesterol accessibility. This demonstrates that ATGL functions downstream of ACC1 and ACSL. Small changes to PM accessible cholesterol levels are still observed when ACC1 is inhibited in ATGL knockout cells, however, these changes are no longer reversible by fatty acid treatments, demonstrating that without ATGL, fatty acyl-CoA availability is no longer sensed.
    d. ATGL inhibitor treatment causes a rapid reduction in PM accessible cholesterol with a temporal profile that mirrors the kinetics of ACC1 inhibition, but in the opposite direction. This suggests that ACC1 and ACSL loss lead to activation of ATGL, since they have the opposite effect to ATGL inhibition.
    e. Targeted mass spectrometry shows that ACC1 inhibition causes the hydrolysis of triacylglycerol (TAG) into diacylglycerol. This demonstrates that ACC1 inhibition causes elevated ATGL activity because ATGL is the rate limiting enzyme in TAG hydrolysis.

    The question of how ACC1 regulates ATGL was raised by us in our previous manuscript Discussion section (previous text lines 467-474), and by Reviewer #3 (Major Point (3)). These findings address this mechanistic question.

2. **LDs are not required for the PM accessible cholesterol changes we observe in our study.** We generated a triple knockout cell line lacking DGAT1, DGAT2 and DIESL, the three enzymes that are known to synthesize TAG- we call this the TAG-TKO cell line (PMID: 37648867). These cells do not have LDs, and LDs are not formed by exogenous fatty acid treatments or by blocking ATGL activity. Strikingly, these cells have normal PM accessible cholesterol levels. Additionally, TAG-TKO cells behave identically to wild-type (WT) cells: their PM accessible cholesterol levels rise when ACC1 or ACSLs are lost (which we propose activates ATGL), and their PM cholesterol levels are reduced by ATGL inhibition (**new Figure 5**).

3. **ATGL activation significantly increases the abundance of polyunsaturated diacylglycerols (DAGs), whereas ATGL inhibition yields the reciprocal effect (new Figure 6a-c).** We performed targeted lipidomics in both WT and TAG-TKO cells to determine which lipid species are altered in response to ATGL activation or inhibition. Regardless of TAG levels, we saw significant changes in DAGs, suggesting that ATGL activity can remodel the cellular DAG pool independently of TAGs. DAGs are used to generate the abundant PM lipids phosphatidylcholine (PC) and phosphatidylethanolamine (PE) through the Kennedy pathway. Lipidomics revealed that both PC and PE polyunsaturation was elevated upon ATGL activation (and was lowered by ATGL inhibition). Visual inspection supported this precursor-product relationship, showing a striking conservation of acyl-chain signatures between DAGs and PC/PE (**Figure 6d-i, underlined lipids**).

4. **ATGL activation raises PM fluidity and cholesterol accessibility.** We use the membrane fluidity probe Laurdan to measure PM fluidity changes with confocal microscopy. This revealed that PM fluidity is elevated when ATGL is active (in concordance with elevated lipid unsaturation), and PM fluidity is reduced (membranes are more rigid) when ATGL is inhibited. Multiple previous studies have demonstrated that saturated lipids (including sphingolipids and phospholipids) sequester cholesterol more efficiently because of increased van der Waal interactions; in contrast, polyunsaturated lipids raise the accessibility of cholesterol (PMID: 24138860, 24000774). Our detection of increased PC/PE unsaturation and a concordant increase in PM fluidity provides the mechanistic basis for why we observe elevated PM accessible cholesterol in this study.

In conclusion, our data supports a model in which ACC1 inhibition activates ATGL to generate polyunsaturated DAGs, which are channeled into PC and PE to remodel PM fluidity and elevate cholesterol accessibility.

We hope you appreciate our updated manuscript. The accessible cholesterol field has largely focused on the role of sphingomyelin in sequestering cholesterol; here we show that phospholipid saturation can have an equally potent effect (direct comparison in **Figure 2f**). Our data also demonstrates that ATGL impacts the lipidome independently from its role in TAG hydrolysis, an insight that was only made possible by genetic ablation of all three TAG-synthesizing enzymes (**Figure 5, 6**). Finally, we clarify a mechanism for how ACC1 and ACSLs indirectly regulate ATGL, which has not been clearly linked or broadly accepted in the literature (**Figure 3**).

REVIEWER COMMENTS

Comments from Reviewer #1

1. This manuscript claims to identify ACC1 as a specific regulator of plasma membrane cholesterol through lipid droplet-mediated trafficking. However, careful examination reveals that the authors are documenting non-specific metabolic collapse that affects all cellular lipids, not a cholesterol-specific regulatory mechanism.
Think about what happens when you inhibit ACC1: fatty acid synthesis stops immediately, lipid droplets start breaking down through ATGL, fatty acyl-CoA pools crash, membrane phospholipid composition shifts. By hour 6, these aren't normal membranes anymore. The cholesterol changes are just one piece of this larger metabolic disruption.

We appreciate the reviewer's comment that ACC1 inhibition will change the organization of cellular lipids. We no longer claim that ACC1 is a specific regulator of plasma membrane cholesterol. Instead, as you suggest, we show that ACC1 inhibition leads to a lipidome remodeling response. However, we do not believe that the

changes in cholesterol accessibility that we measure in this study are caused by a generalized metabolic collapse for three reasons.

First, the changes in cholesterol accessibility that we observe with ACC1 loss can be reversed by genetic or pharmacological ablation of ATGL (**Figure 3e-g**). If a metabolic collapse is triggered by fatty acid starvation, then ATGL loss would be expected to worsen this collapse since ATGL functions to restore fatty acid levels by hydrolyzing TAGs. The fact that ATGL inhibition reverses the effects of ACC1 inhibition argues against this phenotype being caused by a fatty acid starvation-induced metabolic collapse. Instead, we propose that ATGL is the downstream effector of the PM cholesterol changes we observe: PM cholesterol accessibility is elevated when ATGL is active and lowered when ATGL is inhibited.

Secondly, we now report global lipidomics data which reveals that only a small subset of lipids show reciprocal changes in response to ATGL activation and ATGL inhibition. We nominate a small subset of lipids, notably polyunsaturated DAGs, PCs and PEs, as contributors to the PM cholesterol phenotype (**Figure 6**). The observation that limited lipidomic changes correlate with PM cholesterol accessibility changes suggests that the PM cholesterol phenotype is not triggered by a global metabolic collapse.

Finally, we show that many lipids do not change in response to ACC1 inhibition. Phosphatidylinositol, phosphatidylserine, and sphingomyelin are not affected by ACC1 inhibition (**Extended data Fig. 3, Extended data Fig. 7** and **Extended data Fig. 8**). Additionally, while many polyunsaturated PCs are elevated in abundance in response to ACC1 inhibition, the most abundant PC species, including 16:0_16:1, 16:0_18:1, 18:0_18:1, and 18:1_18:1 do not change (**Fig. 6d**). Some lipids do indeed change in response to ACC1 inhibition, including phosphatidylglycerol and plasmalogens, but these changes are not reversed by ATGL loss, and thus these changes cannot explain the PM cholesterol phenotype. Altogether, we have reframed this manuscript to demonstrate that ACC1 loss causes a selective remodeling of the lipidome that in turn results in changes in cholesterol accessibility.

2. Cholesterol flips between membrane leaflets in microseconds (PMID: 30633868, 34781553). It equilibrates across cellular membranes in seconds to minutes. But ACC1 inhibition takes 2-6 hours to change PM cholesterol levels. That's more than a 1000-fold difference. When you see changes that slow, you're not looking at cholesterol transport anymore - you're watching the entire cellular lipid metabolism fall apart and reorganize.

We agree with the reviewer that the slow kinetics of the phenotype are inconsistent with direct cholesterol transport, and we no longer implicate cholesterol transport as the underlying mechanism for the changes we observe. We now provide evidence that, as you suggest, cellular lipid metabolism is reorganized in response to ACC1 inhibition. As you point out in your comment #1, ACC1 inhibition results in depletion of fatty acyl-CoA pools– a process that starts immediately but with gradually worsening effects over a ~2-6 hour time scale. We provide evidence (discussed in our Major New Findings point 1), that this process stimulates ATGL activation. ATGL is the downstream critical mediator that leads to changes in PM fluidity, which in turn modulates cholesterol accessibility.

3. The RNA-seq data actually support this interpretation (Fig. 6g-h). Both SREBP1 (fatty acid genes) and SREBP2 (cholesterol genes) pathways activate simultaneously. If this were specifically about cholesterol, you'd only see SREBP2 activation. Instead, the cells are mounting a panic response to global lipid depletion.

We appreciate your comment that cells mount a variety of responses to mitigate changes in lipid availability, which was helpful for reframing our manuscript. We believe that our RNA sequencing data is indicative of a coordinated lipid metabolic reprogramming which impacts both cholesterol and fatty acid biosynthesis pathways. The RNA sequencing does not strongly indicate a global panic response. For example, Gene Set Enrichment Analysis of the RNA sequencing data revealed that "cholesterol metabolism" was the most significantly enriched pathway induced by ACC1 inhibition, followed by "cholesterol synthesis disorders" (**Fig. 7j**). In contrast, the RNA sequencing data did not reveal the activation of canonical stress-response pathways such as the ER stress response pathway.

As you point out, our data shows that SREBP1 is also activated by ACC1 inhibition. This is consistent with the field which shows that SREBP1 and SREBP2 are both activated in response to ER cholesterol

depletion, since SCAP (which tethers SREBP1 and SREBP2 in the ER) is a cholesterol sensor (PMID: 11994399). In support of this, SREBP1 was also activated by blocking the rate-limiting enzyme in cholesterol biosynthesis, HMGCR (**Fig. 7i**). We do note that the magnitude of SREBP1 activation is lower for HMGCR inhibition compared to ACC1 inhibition, though this could be for a variety of reasons. For example, SREBP1 is sensitive to factors such as polyunsaturated fatty acids and insulin (PMID: 20145241, PMID: 22927400). Altogether, our RNA sequencing data indicates that cells sense alterations to cholesterol levels; they do not strongly indicate ER stress or global lipid panic.

We find it very intriguing that SREBP2 signaling/cholesterol metabolism is the most altered pathway activated in response to ACC1 inhibition– it is even more significant than the "fatty acid and lipoprotein transport" pathway which was the third enriched pathway. This discovery is important to report because ACC1 is a core metabolic enzyme and human drug target which has not yet been linked to cholesterol metabolism. Our revised manuscript provides a mechanistic explanation for SREBP2 activation: ACC1 inhibition stimulates ATGL activity by depleting fatty acyl-CoA pools; ATGL activation leads to elevated DAG unsaturation which is shunted into PC and PE biosynthesis pathways. Finally, elevated PC and PE unsaturation raises PM fluidity, which reduces cholesterol transport out of the membrane (**Fig. 6** and **Fig. 7a-c**).

4. The membrane blebbing in the hepatocytes (Fig. 6e) further suggests these membranes are fundamentally compromised.

We thank the reviewer for this observation. We note that membrane blebbing is also observed in wild-type hepatocytes, indicating that this phenotype is not specific to *Acc1/Acc2* double-liver knockout (dLKO) hepatocytes and does not reflect fundamentally compromised membranes. We have added a more clear image of both wild-type and dLKO mouse hepatocytes to the revised manuscript (**Extended data Fig. 9c**, **d**) to better illustrate the membrane blebbing phenotype in each. See example here:

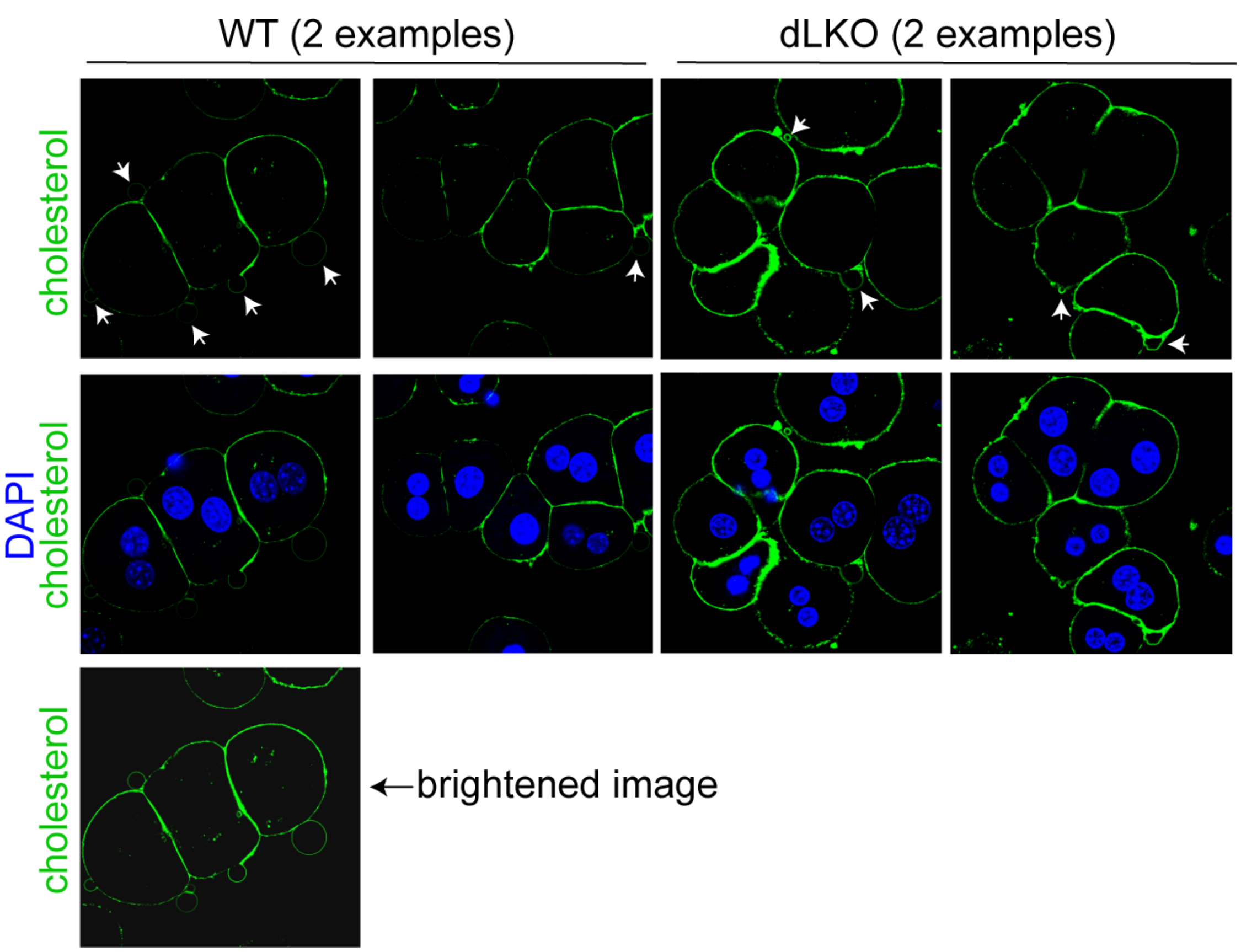

Added to main text document, **Extended data Figure 9c and **d**.*

5. Another telling sign: the phenotype is irreversible. ACC1 knockout cells maintain elevated PM cholesterol for weeks despite having functional cholesterol export machinery. That's not how regulated processes work - they're reversible. Once you've broken lipid metabolism this badly, the cells enter a new steady state with altered membrane properties that happen to accommodate more cholesterol.

We thank the reviewer for raising this concern. We have removed all language from the text claiming that this is a regulated process. We would like to point out that the loss of many proteins that mediate cellular cholesterol homeostasis cause prolonged alterations to PM cholesterol levels. For example, we show in **Fig. 1a** and **Extended data Fig. 1e** that GRAMD1A/GRAMD1B/GRAMD1C knockout causes prolonged increases in PM cholesterol, despite functional cholesterol export machinery like LXRs/ABCA1 efflux. Similarly, we and others have shown that NPC1 knockout, which impairs LDL-derived cholesterol export from lysosomes, causes reduced PM accessible cholesterol that does not recover (**Extended Fig. 2b,d**; also PMID: 32690708 Fig. 5).

We would also like to emphasize that the ACC1 phenotype is in fact reversible. Specifically, inhibiting or knocking out ATGL largely restores PM cholesterol to normal levels. In treatments where ATGL inhibition is performed subsequently to ACC1 loss, PM cholesterol is restored (**Extended data Fig. 5h**). This shows that the ACC1 phenotype is reversible, though we agree that cells appear to lack an intrinsic mechanism to respond and turn off the activity of ATGL that we observe. While ACC1 loss may have many effects on cellular metabolism, our data demonstrates that the effects that lead to PM cholesterol changes can be reversed by blocking ATGL.

6. The oleic acid rescue experiments need much better controls. Oleic acid doesn't just restore lipid droplets - it inhibits ATGL (PMID: 23297223), triggers autophagy pathways (PMID: 25586377), changes membrane fluidity because it's unsaturated, and activates various stress responses. When the authors see partial cholesterol "rescue" with oleic acid, which of these effects is responsible? They don't know. They need to test saturated fatty acids (do they also work?), use specific ATGL inhibitors (does blocking catabolism alone help?), and check whether the rescue correlates with normalized membrane composition rather than just LD numbers.

We now include many controls for oleic acid treatments. First, we show that palmitic acid also rescues ACC1 inhibited cells (**Extended data Fig. 5g**). This shows the changes are not related to fatty acid unsaturation. Secondly, we show that fatty acids restore PM cholesterol levels in cells that lack LDs (and do not make lipid droplets in response to fatty acids), which rules out the LD contribution (**Extended data Fig. 7b**,**c**). We test whether triggering autophagy causes PM accessible cholesterol changes, which reveals minor effects compared to ACC1 inhibition (**Revision Figure 1a**). In support of your assessment, <u>blocking ATGL alone does restore PM cholesterol</u>. This demonstrates that ATGL is acting downstream of ACC1. Finally, we show that cells lacking ATGL are no longer responsive to fatty acids (**Fig. 3i**). As you state (we now enthusiastically agree) long chain fatty acyl-CoAs inhibit ATGL. Thus, adding oleic acid or palmitic acid reverses the ACC1 phenotype by restoring the fatty acyl-CoA pools that inhibit ATGL.

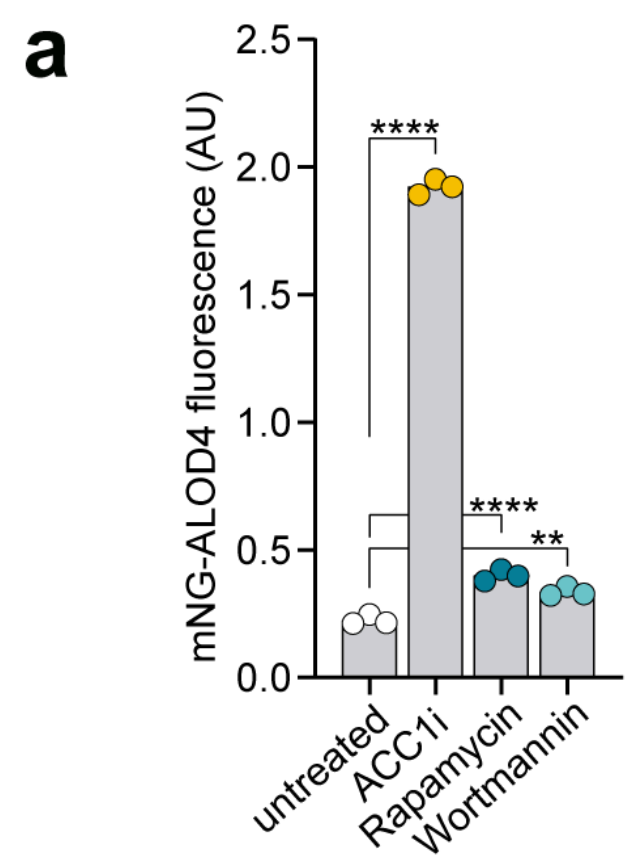

Revision Figure 1. a Flow cytometry measurement of PM accessible cholesterol in cells left untreated or treated with the ACC1 inhibitor Firsocostat, 5 µM Rapamycin (a potent inducer of autophagy) or 5 µM Wortmannin (a potent inhibitor of autophagy). Statistical significance was determined with an ordinary one-way ANOVA followed by a Sidak correction. Exact *p*-values are as follows: untreated vs ACC1i $p<0.0001$, untreated vs Rapamycin $p<0.0001$, and untreated vs Wortmannin $p=0.0011$.

7. The correlation between lipid droplet abundance and PM cholesterol is real and interesting, but the causation isn't what the authors think. They're proposing that LDs directly buffer PM cholesterol. More likely, LD loss is a marker of the metabolic catastrophe that indirectly affects cholesterol through wholesale changes in membrane composition. When you lose LDs, you're not just losing cholesterol storage sites - you're documenting the collapse of normal lipid metabolism.

As discussed in our section on “New Major Findings” we no longer believe that LDs are linked to the PM cholesterol changes we observed. Cells lacking the TAG synthesizing enzymes DGAT1, DGAT2, and DIESL (TAG-TKO cells) have no observable LDs. However, these cells show robust ATGL-dependent changes in PM cholesterol accessibility. In light of our new data, we agree completely that LD loss is simply a consequence of ATGL activation, and that this is not causative for our phenotype. The abundance of LDs in our experiments turned out to be a very robust metric of ATGL activity, and nicely demonstrates that ACC1 inhibition triggers ATGL activation, but this is unrelated to the PM cholesterol changes we observe.

8. Do a proper lipidomics time course. Show us what happens to all lipids, not just cholesterol.

We thank the reviewer for this valuable suggestion. We have, in part, addressed this in response to your comment #1. We did not perform a time course because, after discovering that LDs are dispensable for our phenotype, we needed to identify which lipid species were changing in our assays (and with limited financial resources, a full time course was restrictive). With the knowledge that ATGL inhibition reversed the ACC1 phenotype (**Fig. 3e-g**), and that ATGL inhibition alone had the opposite phenotype to ACC1 loss (**Fig. 3j**), we decided to perform lipidomics on ACC1 and ATGL inhibited cells (and the combination). Additionally, we performed lipidomics on both WT and TAG-TKO cells, both to further characterize this engineered cell line and to find lipids that were equally changed in both cell lines (given their equivalent PM cholesterol changes). We reasoned this set of conditions would enable us to identify the relevant lipid changes that result in either elevated or reduced PM cholesterol accessibility. This was extremely fruitful, and allowed us to determine that DAGs, PC and PE increase or decrease in unsaturation in response to ATGL activation or inhibition, respectively. We have also included two supplementary figures (**Extended data Fig. 7** and **8**) showing the changes in other lipid species including PI, PG, PS, PE-o, PE-p, LPC, LPE, LPI, LPG, LPS, sphingomyelin,

ceramide, hexosylceramide, lactosylceramide and cholesterol esters. As discussed in our manuscript, these lipids did not show ACC1 inhibition-dependent changes that were reversed by ATGL loss of function.

9. Test whether maintaining normal membrane composition (not just adding oleic acid) prevents the cholesterol changes.

We have shown reversibility of the ACC1 inhibition phenotype in multiple ways. First, we showed that exogenous oleic (18:1) or palmitic acid (16:0) addition was able to partially restore PM cholesterol levels (**Fig. 3h**, **Extended data Fig. 5g**, and **Extended data Fig. 7c**). We propose that this restores fatty acyl-CoA pools, which then inhibit ATGL (PMID: 24440819). The key findings supporting this are discussed above in the "Major new findings" section. Most importantly, PM cholesterol changes induced by ACC1 loss are prevented by blocking ATGL activity (either using a CRISPR knockout or with pharmacological approaches). Our data demonstrates that ATGL is the downstream mediator of the effects we observe.

10. Include proper controls for oleic acid's many effects.

We believe we have addressed this in response to your comment #6 (please see above).

11. Consider reframing this as a study of metabolic disruption rather than specific cholesterol regulation.

We appreciate the reviewer's suggestion to consider reframing our work in the context of metabolic disruption. As discussed above, we do not believe the changes we observe in response to ACC1 inhibition are due to a global metabolic disruption because they are largely reversed by inhibiting ATGL. The combination of ACC1 and ATGL inhibition would be expected to worsen fatty acid starvation because cells will be unable to scavenge fatty acids from TAG when ATGL is inactive. Instead, we observe that ATGL loss mitigates the ACC1 phenotype- this argues that the changes we observe are not due to a metabolic collapse caused by reduced fatty acids.

A second signature that ACC1 loss-of-function does not alter PM cholesterol by triggering metabolic disruption comes from mass spectroscopy on TCA cycle intermediates, glycolytic metabolites, redox cofactors, and acyl-carnitines involved in fatty acid beta-oxidation. These metabolic changes were quantified for multiple treatments including: ACC1 inhibition and ACSL inhibition (both of which raise PM cholesterol accessibility), FASN inhibition (with the inhibitor C75, which did not alter cholesterol accessibility- see **Fig. 4e**), and finally with Lovastatin (an FDA-approved statin that inhibits HMGCR to block cholesterol biosynthesis). We observe that TCA cycle intermediates are not dramatically altered across all treatments (**Revision Figure 2a**). In contrast, glycolysis metabolites are lowered in response to ACC1 and HMGCR inhibition, and are less responsive to ACSL and FASN inhibition; this trend does not correlate with the PM cholesterol changes we observed (**Revision Figure 2b**). The ratio of NADH/NAD+ or GSH/GSSG were not significantly different between treatments, and the overall abundance of these metabolites does not correlate with PM cholesterol changes (**Revision Figure 2c**). Finally, acyl-carnitine formation was significantly elevated in response to HMGCR inhibition (PMID: 38641905), and reduced in response to ACSL inhibition (**Revision Figure 2d**). The latter effect is expected because the enzyme carnitine palmitoyltransferase 1 (CPT1) requires acyl-CoAs to make acyl-carnitines (fatty acids must first be charged with CoA before they can be converted to acyl-carnitines). Once again, the changes in acyl-carnitines observed do not explain the changes in PM cholesterol observed in our study. Altogether, we do not find evidence that a generalized metabolic collapse caused by fatty acid starvation is causing the PM cholesterol phenotypes we observe.

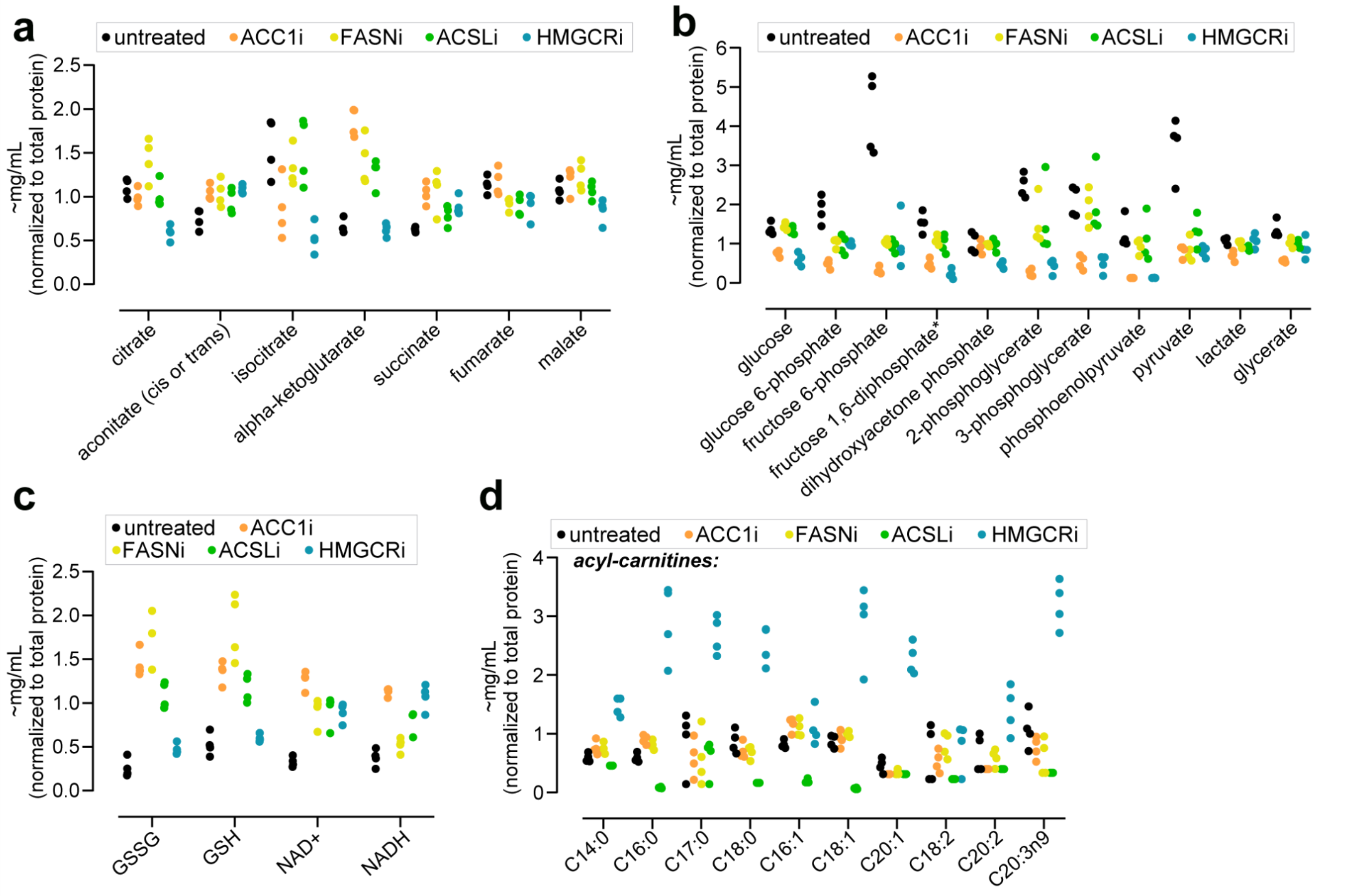

Revision Figure 2. a-d Mass spectrometry analysis of cells grown in 5% lipoprotein-depleted serum media for 24 hours and then either left untreated or treated for 6 hrs with inhibitors targeting ACC1 (ACC1i; 30 μM Firsocostat), FASN (FASNi; 10 μM C75), ACSLs (ACSLi; 5 μM Triacsin-C) or HMGCR (HMGCRi; 10 μM Lovastatin). Graphs show the relative abundance of metabolites in the TCA cycle (**a**), glycolysis (**b**), redox metabolism (**c**), and fatty acid beta-oxidation (**d**). *fructose 1,6-diphosphate is indistinguishable from glucose 1,6-diphosphate and myo-inositol diphosphates.

12. There's clearly something interesting happening with ACC1 and cellular lipids. The connection to lipid droplets is real. But calling this specific cholesterol regulation misrepresents the biology. The authors are seeing the cholesterol component of a much larger metabolic phenomenon. That's still worth publishing, but it needs to be described accurately.

We greatly appreciate the reviewer's comment that we should reassess our data and be cautious in the conclusions we draw. We have avoided describing our observations as a regulated process. We now show that many lipid species are altered, which together modulate the accessibility of cholesterol in the membrane. We appreciate that this reviewer thinks these findings are interesting and worth publishing.

13. The therapeutic implications they mention for NPC disease are premature given that they're inducing broad metabolic dysfunction. You can't fix a specific trafficking defect by breaking the entire system in a different way. The cells might have similar cholesterol levels, but for completely different reasons.

We have removed all mentions of therapeutic implications for NPC disease. We have also moved the results of this screen (and its follow-up) from the main figure to a supplementary figure (**Extended data Figure 2**). In the main text, we now simply state the results of the screen and that we were able to validate the screen results.

14. I'd encourage the authors to embrace what they've actually discovered - that ACC1 inhibition causes a cascade of metabolic changes affecting all cellular lipids. That's interesting biology. Forcing it into a "specific cholesterol regulation" story does a disservice to their own data.

We appreciate you encouraging us to view lipid changes more broadly. We believe we have done this and it has significantly strengthened our manuscript. Our data supports that ACC1 inhibition triggers a lipidome remodeling response. We show that DAG, PC and PE are specific lipids that are remodeled (becoming more or less unsaturated) in ways that predictably influence the accessibility of cholesterol.

Comments from Reviewer #2

Wijesinghe et al identified ACC1 as a regulator of cholesterol transport from the plasma membrane in a genome-wide screen. Knockdown of ACC1, which is the rate-limiting gene in fatty acid synthesis, unexpectedly reduces cholesterol trafficking, but they find that increasing lipid droplet mass by oleic acid loading or by ATGL knockout restores cholesterol trafficking. They provide evidence that ACC1's effect on cholesterol trafficking is independent of ACAT1/2, NPC1 and new synthesis. The critical assay in this work is to measuring binding of mNeonGreen-ALOD4, which I think is reliable under the conditions used. The effects measured are quite large and seem statistically robust.

1. They test their hypothesis by manipulating proteins that directly affect lipid droplet volume. Knockout of ACC1 under serum starvation reduces LD number. They claim that knockout of DGAT1 and DGAT2 failed to eliminate lipid droplets, but these were under 5% serum rather than starvation conditions. They show that ATGL knockout (which reduces catabolism of stored lipid in lipid droplets) allows normal trafficking of cholesterol even with ACC1 knockout, consistent with their hypothesis.

We appreciate this analysis of our work. We did not previously claim that knockout of DGAT1/2 failed to eliminate LDs- we showed that DGAT1/2 <u>inhibition</u> failed to eliminate LDs after 24 hours. To address your point that these experiments were performed in 5% serum, we here show that inhibiting DGAT1 and DGAT2 in lipoprotein-depleted serum also failed to eliminate LDs after 24 hours (**Revision Figure 3a**). We also tested DGAT1 and DGAT2 inhibitors in *DIESL* knockout (*DIESL*-KO) cells, which showed reduced but not eliminated LDs (**Revision Figure 3b**). This supports our model that blocking LD synthesis causes slow kinetics of observed LD loss. In contrast, ACC1 inhibition caused total elimination of LDs within ~20 hours. As you identified in your assessment, the most compelling evidence that ACC1 inhibition triggers LD catabolism through ATGL is that *ATGL*-KO cells were resistant to LD loss triggered by ACC1 inhibition.

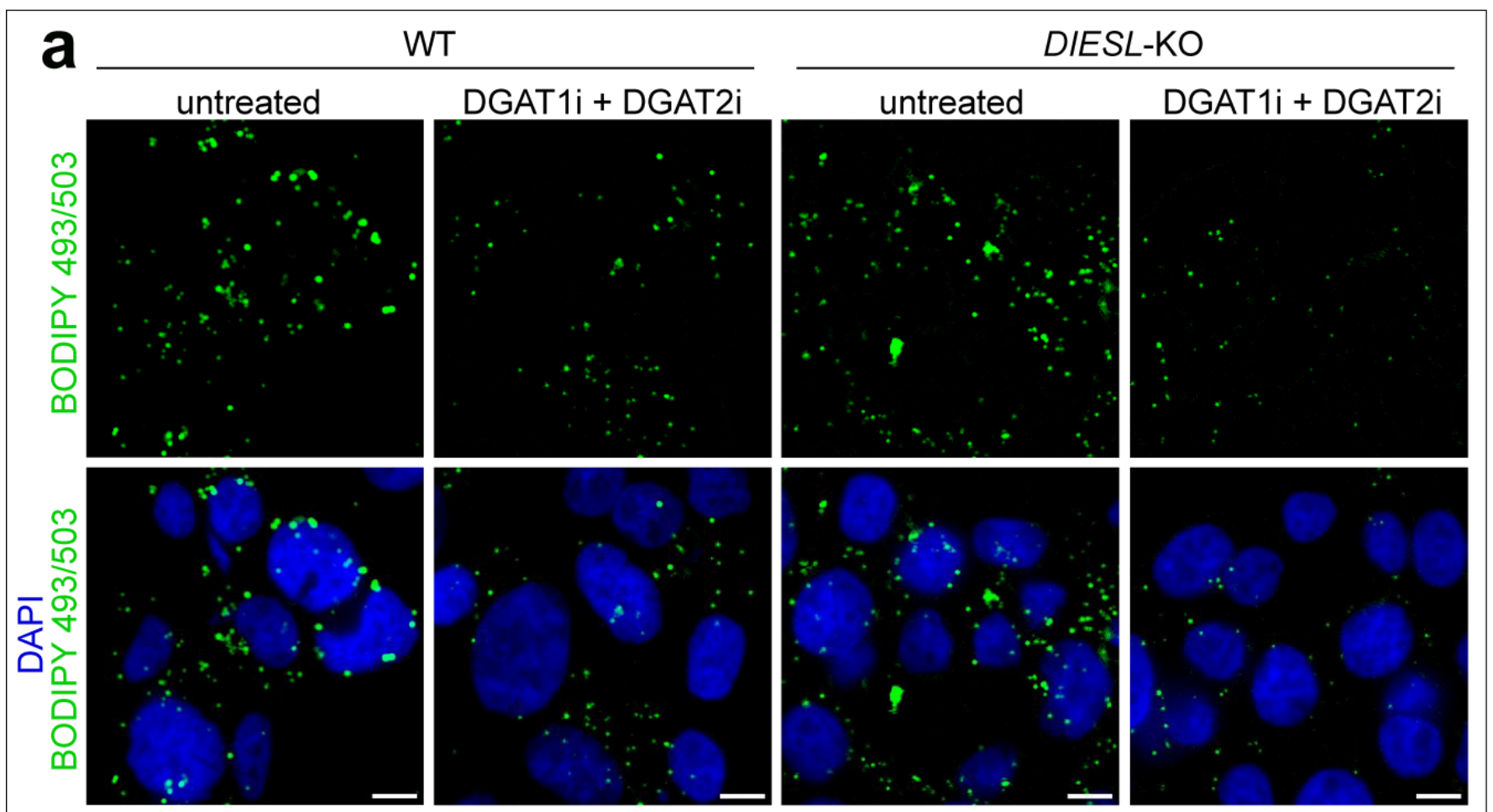

Revision Figure 3. a WT and *DIESL*-KO HAP1 cells were grown in 5% lipoprotein-depleted serum media for 24 hours and then treated with DGAT1 (DGAT1i; 20 μM T863) and DGAT2 inhibitors (DGAT2; 10 μM PF-06424439) for 24 hours in 5% lipoprotein-depleted media. Cells were then fixed an stained with BODIPY 493/503 to measure LDs and DAPI to detect nuclei; the scale bars are 5 microns.

2. It would be useful to test ATGL overexpression (perhaps tagged with mCherry), which can considerably reduce lipid droplet mass, at least in some cell lines. In their model, ATGL overexpression (provided it reduced lipid droplet mass) would be expected to reduce cholesterol trafficking even in the presence of normal levels of ACC1.

We thank the reviewer for this thoughtful suggestion and agree that ATGL overexpression is an elegant strategy to further test our model. We transiently overexpressed mCherry-tagged ATGL (mCherry-ATGL) in HAP1 cells and observed that LDs were still present. mCherry-ATGL was enriched at LDs, suggesting that it was expressed well (and localized properly), but was inactive. We further attempted to combine ATGL-mCherry overexpression with a 24 hour treatment with the ABDH5 activator SR-4995 (PMID: 26411340), however, LDs still remained (**Revision Figure 4a**). We do not fully understand why LDs still remained in our transfected cells; however, as described in our "Major new findings" section and our revised manuscript, we now provide compelling evidence that ATGL activation can elevate PM accessible cholesterol in cells lacking LDs. In cell lines lacking DGAT1, DGAT2 and DIESL, LDs were absent, and yet we still observed reduced PM cholesterol accessibility in response to ATGL inhibition, and elevated cholesterol accessibility in response to ATGL activation. This data strongly suggested to us that LDs are not causative for the PM cholesterol phenotype–therefore, we did not further pursue this line of experiments. The necessary factors (protein binders or small molecule regulators) that are required for ATGL to modulate lipids independently of LDs will be important to elucidate in future studies.

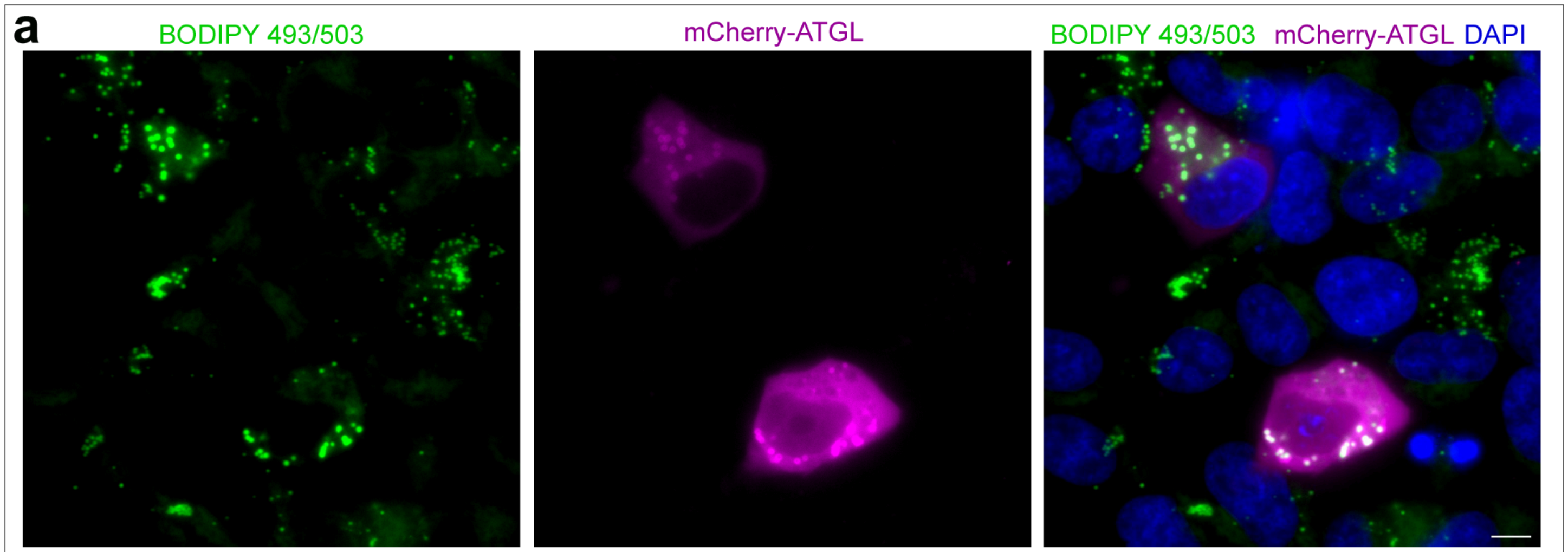

Revision Figure 4. a HAP1 cells were seeded in live cell imaging chambers (Fisher, Catalog # NC1930679) and then transfected with mCherry-ATGL. 16 hours later, cells were moved into 5% lipoprotein-depleted serum media with the ABHD5 activator SR-4995 (10 µM, MedChemExpress Catalog # HY-111952) for 24 hours prior to live-cell imaging. LDs were detected by adding BODIPY 493/503 for 30 minutes at 37°C prior to imaging and nuclei were detected with NucBlue Live ReadyProbes Reagent with Hoechst 33342 (Thermo Fisher Scientific, Catalog # R37605). The scale bar is 5 microns.

3. Overall, this is an interesting paper with good quality data. Further work would be needed to achieve a complete mechanistic understanding of what's going on, but I think this would be of use to the cell biology community.

We greatly appreciate your enthusiasm for our manuscript. We believe our revised manuscript has revealed new mechanistic insights. We now provide evidence that ACC1 inhibition depletes fatty acyl-CoA pools, leading to ATGL activation. While this is mechanistically logical and ATGL is known to be regulated by fatty acyl-CoA (PMID: 24440819), we provide evidence that this regulation holds true in cell models and make this connection clear in the literature for the first time. For example, we show that ACC1 inhibition triggers ATGL-dependent TAG hydrolysis with mass spectrometry and LD microscopy (**Fig. 4h-j**). Secondly, our revised manuscript shows that in cells lacking LDs (DGAT1/DGAT2/DIESL triple knockout cells), ATGL activation causes elevated DAG, PC and PE unsaturation. Mechanistically, we propose that PC and PE unsaturation ultimately raises cholesterol accessibility (PMID: 24138860, 24000774). We believe these new mechanistic insights significantly strengthen the manuscript.

Reviewer #3 (Remarks to the Author):

The manuscript reports identification of plasma membrane (PM) cholesterol regulation pathway involving ACC1 and lipid droplets (LDs). The work potentially highlights a novel functional role of an essential organelle (LDs) and is thus likely to stimulate broad interest.

However, in its present form, the proposed mechanistic links between ACC1 and PM cholesterol regulation remain not fully substantiated, lacking direct evidence / molecular mechanism, as well as datasets that can unambiguously rule out alternative mechanisms. The following queries should thus be addressed in full, in backing up the authors' claims and to strengthen the key conclusions.

We further request the authors to doubly reassure the novelty statements made in the manuscript, and tone down or clarify such claims as necessary. For instance, Abstract makes novelty claims about what appear to be knowledge already available in the literature, such as: "Mechanistically, we find that ACC1 regulates lipid droplet catabolism…." The fact that ACC1 regulates LDs is established in the field (via AMPK1 kinase phosphorylation suppressing fatty acid synthesis that otherwise supports LD anabolism), meaning also that several of the datasets in main fig. 5 do not represent new knowledge. Thus, statements as such throughout the manuscript should be tightened up and carefully proof-checked.

We have incorporated your feedback and we believe this has improved the quality of our manuscript. For example, we have re-written the manuscript to make it approachable to a broader audience. We have also toned down the novelty statements throughout the manuscript. We have made new CRISPR knockout cell lines to address your concerns on the use of inhibitors in our study, and we have addressed issues of statistical tests used and experimental controls.

We disagree that it has been reported that ACC1 regulates LD **catabolism** in the literature. As you mention "AMPK1 kinase phosphorylation suppresses fatty acid **synthesis** that otherwise supports LD **anabolism.**" We agree that it is very clear that ACC1, as the master regulator of fatty acid biosynthesis, regulates LD formation. It has not been shown, however, that ACC1 inhibition can trigger the breakdown of LDs via ATGL. We have made this distinction more clear in the text. In our section called "ACC1 inhibition triggers ATGL activation and triacylglycerol hydrolysis" (associated with Figure 4), we add clear statements aiming to distinguish between ACC1's role in anabolism vs catabolism of LDs. For example we state: "ATGL activation triggers the hydrolysis of TAG and thus the catabolism of LDs. ACC1 has primarily been viewed as a driver of LD biogenesis through its role in de novo lipogenesis rather than a regulator of LD breakdown via ATGL…" and also "To determine whether LD depletion stemmed from impaired fatty acid synthesis or accelerated catabolism, we compared the kinetics of LD loss induced by ACC1 inhibition to known LD synthesis inhibitors…." We hope this has added clarity to the text.

Major points:

1. ***Writing / accessibility***

The manuscript is currently written for the readership of field-specialised journals such as Journal of Lipid Research– thus issues below do need major overhaul such that the paper can be made more accessible for broader readership that could be ultimately suited to journals such as Nature Communications. Just to outline a few issues – they are not exhaustive however:

1a. Introduction has a lot of gene acronyms largely without briefly mentioning to the reader what they are / their key roles. Currently too much "jargons" as such is summarised in 3 short paragraphs in the intro, and for almost each abbreviation, readers are forced to look up online what these individual genes/proteins do - at least >10-15 proteins, without spelling out abbreviations (except for CH25H), are all squashed into a single page intro. This issue continues – for instance, what is NPC1, what is LXR, what are their known roles and organelle-location etc., in the context of this paper (?)

Thank you for bringing this to our attention. We have addressed this issue. Every gene name throughout the text is spelled out and its function is stated the first time the gene is mentioned. We maintain that it is important to mention many of these genes because it 1) sets up what is known/accepted in the field, 2) it demonstrates that we are not narrowly focused on our hypothesis and instead are more broadly considering different models, and finally 3) this paper is entwined in transcriptional signaling cascades and metabolic biosynthetic pathways. To understand the key findings and importance of the work, readers must grasp the function of multiple genes. To make this more approachable, we have removed unnecessary jargon and only mention genes that we feel are absolutely necessary for conveying the message of the work. For example, we no longer mention SCAP, StarD4, StarD5 or StarD6. We have entirely re-written the introduction of this paper, and feel it is stronger and

more clear now. Additionally, we have added at least one schematic to each figure of the paper which clearly outlines the relevant genes discussed in that figure.

1b. It would greatly assist the reader if what is known vs. unknown or unclear, etc. can be presented as a supplementary figure that can clearly show where these various genes lie in the known cholesterol trafficking pathways and associated LD catabolism / anabolism. I see that main fig 1e has an illustration, but it does not cover all the key genes mentioned in the intro several of which are manipulated (inhibited, knocked out, or generally studied further in this work) in order to derive key conclusions – thus they must be integrated in an illustrative figure, which can be in SI, that provides a more comprehensive and accessible information (than fig 1e)

We have provided two new figures (**Extended data Figure 1a** and **1b**) that show cholesterol trafficking pathways and the genes mentioned in the Introduction section. Additionally, every figure in the text now has one or more schematics that outline the key metabolic steps or conceptual ideas relevant for that particular figure. We understand that our manuscript involves many signaling pathways and biosynthetic pathways, as well as analysis that relies on understanding negative feedback mechanisms and epistasis. We hope that the added figures have made it more approachable for all audiences.

1c. Order of the text and logic is also largely off. For instance, mNeonGreen explanation flies in only in line 115 (Fig 1F) although this assay was used right from the beginning– e.g., Fig. 1A and S1A (line 95 onwards, the paragraph above). Readers are left to look up everything as they progress through every sentence of this paper… Likewise, NCP1 was explained finally on line 139, when its inhibition was executed/discussed in 2 pages above

We have improved the logical flow of the entire manuscript. We now introduce ALOD4 and PFO in the introduction with these sentences: "The discovery and characterization of accessible cholesterol was made possible through the identification of a family of bacterial cholesterol-dependent cytolysin proteins such as Perfringolysin O (PFO) and Anthrolysin O (ALO), which specifically bind to accessible cholesterol. The non-lytic cholesterol-binding domain 4 of PFO or ALO (PFOD4 or ALOD4) is an invaluable tool to interrogate the localization, function, and regulation of accessible cholesterol."

We have also introduced NPC1 at the appropriate time, and include an explanation of the function of this protein: "Niemann-Pick type C1 protein (NPC1) is required to transport LDL-derived cholesterol out of the lysosome; mutations in NPC1 are the most common cause of Niemann-Pick disease type C (NPC disease), a fatal syndrome." We hope that you find the logical flow to be greatly improved.

2. Consistency and rigor

2a. Most of the conclusions thus far are derived from gene deficiency test, often through the use of multiple inhibitors. In the case of ATGL, it is clear as discussed in lines 324-328 that there are phenotypic differences between CRISPR KO and inhibitor treatment. That being said, given that many inhibitors are pleotropic / poly-pharmacologic (including not-yet-known-targets etc.), in experiments where inhibitor usage alone has been solely deployed, the corresponding conclusion should be backed up by parallel KO/KD experiments. This includes – but not limited to – AMPK, ACSL, and FASN. [AMPK is key to support the claimed new knowledge (if any) in the context of AMPK / ACC1 (ACC2) – fatty acid – LD regulatory cascade, and the latter two directly affect the level of LDs, especially ACSL inhibition could give similar outcomes as ACC1 KO or inhibition].

We appreciate the reviewer's comment emphasizing the importance of validation using genetic loss-of-function approaches. Elimination of ACSL proteins is not trivial because there are 5 isoforms in human cells (ACSL1, ACSL3, ACSL4, ACSL5 and ACSL6) (PMID: 41288931), this is why the molecule Triacsin-C is commonly used (in over 200 publications) as a method to potently block ACSLs. Regardless, to address your point we generated double-knockout cells lacking both ACSL1 and ACSL3 (*ACSL1/3*-DKO). These cells showed elevated PM accessible cholesterol similar to *ACC1*-KO cells, validating that ACC1 loss and ACSL loss are phenotypically similar (**Fig. 3c**). These *ACSL1/3*-DKO cells showed a slightly reduced effect compared to Triacsin-C (which is not surprising because these cells still have ACSL4-6), so we treated this cell line with an

ACSL4-specific inhibitor (AS-252424). In concordance with our conclusions, ACSL4 inhibition raised PM even further. These data clearly demonstrate that ACSL loss raises PM accessible cholesterol similar to *ACC1*-KO cells.

FASN knockout data were previously included, located in **Extended data Figure 3g-j**. You can now find this same data in the updated manuscript in **Extended data Figure 5a-c** and **Extended data Fig. 6d.** *FASN*-KO resembles *ACC1*-KO and *ACSL1/3*-DKO, further showing that any treatment that reduces cellular fatty acyl-CoA levels (the metabolite that is made through the coordinated effort of these three enzymes) elevates PM cholesterol accessibility. The fact that multiple independent CRISPR knockout cell lines (targeting different genes) causes the same effect rules out the possibility of these effects arising from CRISPR off-targets.

We previously showed that AMPK inhibition with Dorsomorphin (a small molecule used in over 750 publications) causes reduced PM accessible cholesterol. We proposed that this was due to inhibition of ATGL and/or activation of ACC1. To test AMPK loss with genetic approaches, we first attempted to generate CRISPR knockouts of AMPK1, AMPK2 or AMPK1 and AMPK2 together (*AMPK1/2*-DKO). Not surprisingly, the *AMPK1/2*-DKO was lethal to cells, and we also did not isolate *AMPK1*-KOs, suggesting it is also required for cell survival. An *AMPK2*-KO alone did not alter PM, so we used siRNA against AMPK1 in these cells to acutely reduce AMPK1 protein levels. In contrast to our previous data, this did not modulate PM cholesterol, and instead slightly elevated it. A caveat of this experiment is that AMPK1 was still clearly present in these cells (**Revision Figure 5a-b**). As an alternative approach we treated cells with structurally diverse AMPK inhibitors. As you suggest, small molecules can be pleiotropic; however, the chance that all three of the molecules we tested have the same off target effects affecting PM accessible cholesterol is very low. Each of these inhibitors lowered PM accessible cholesterol (**Revision Figure 5c-d**).

In light of the new data we have gathered since revisions (outlined in our "Major new findings" section of this revision), we do not believe that the conclusions we drew with AMPK are key data, nor do they add significantly to our manuscript. We have thus removed all discussion of AMPK in the revised version. We include our attempts to address your concerns here to show you that we did appreciate them and took them into consideration.

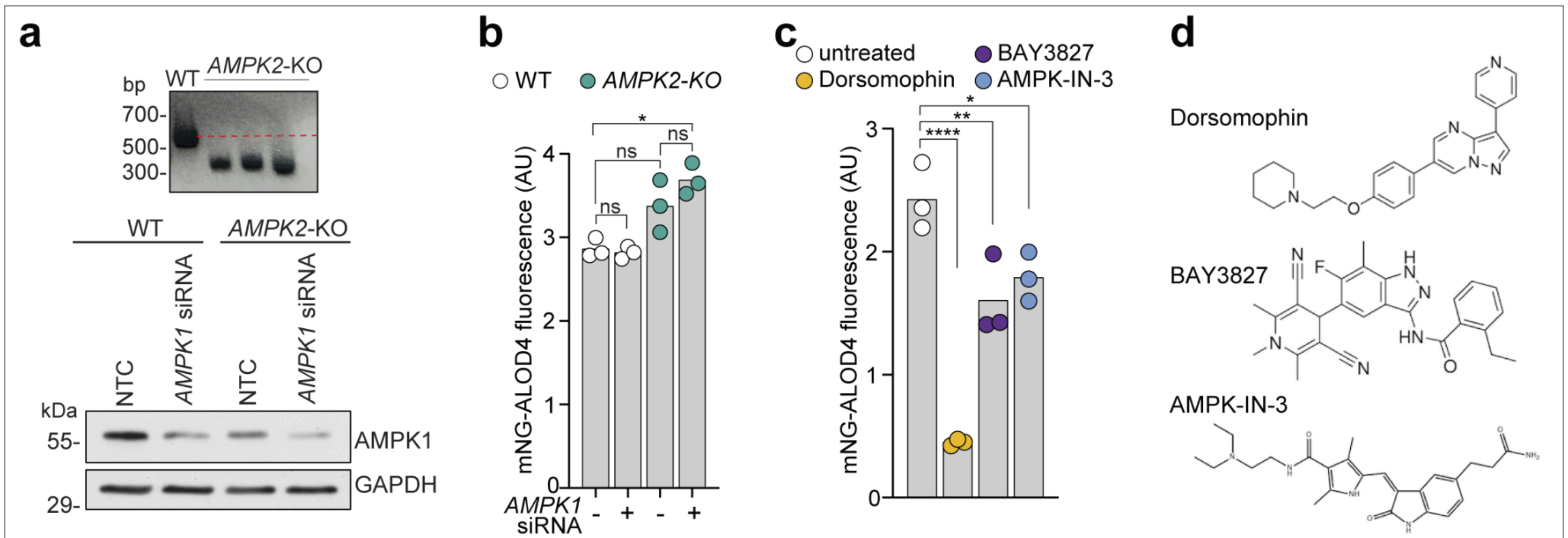

Revision Figure 5. a DNA gel (top) showing AMPK2 knockout clones isolated and Western blot (bottom) showing siRNA knockdown of AMPK1 in wild-type (WT) and *AMPK2*-KO cells. GAPDH is a loading control for the Western blot. NTC= non-targeting siRNA control. **b** Flow cytometry showing PM accessible cholesterol. WT cells were compared to *AMPK2*-KO cells with or without the addition of a NTC or AMPK1 specific siRNA. **c** Flow cytometry of PM accessible cholesterol levels after cells were treated for 20 hours with three distinct AMPK inhibitors (2.5 µM Dorsomorphin, 5 µM BAY3827 and 10 µM AMPK-IN-3). **d** The structure of each AMPK inhibitor. Statistical significance was determined by a two-way ANOVA (**b**) or by a one-way ANOVA (**c**); both tests were followed by a Sidak correction. Exact *p*-values in **b** are: WT NTC siRNA vs. AMPK1 siRNA p=0.9999, WT vs. *AMPK2*-KO (NTC siRNA) p=0.0967, WT (NTC siRNA) vs. *AMPK2*-KO with AMPK1 siRNA p=0.0110, and *AMPK2*-KO cells NTC vs. AMPK1 siRNA p=0.4357. Exact *p*-values in **c** are: untreated vs. Dorsomorpin p<0.0001, untreated vs. BAY3827 p=0.0081, and untreated vs. AMPK-IN-3 p=0.0319.

2b. The authors must do due diligence to rule out potential off-targets in deploying KO/KD. Multiple guide-RNAs (or si/shRNAs where applicable), should have been used in each case and consistently across the board. Please thus remedy these issues in all cases in the present study, to improve rigor in experimental design, and mitigate any possibility of wrong conclusions caused by unintended off-target effects.

We use multiple sgRNAs and inhibitors for all experiments in this paper. For ACC1, we show effects with 5 independent pairs of sgRNAs (see **Extended data Figure 1k**) and two structurally unique inhibitors (**Figure 2d**). ACC1 was also identified to be important in our unbiased Gene Trap random insertion mutagenesis screen. For all assays we assess at least three independently isolated clones; the chance of an off-target sgRNA effect in multiple clones is not likely. We also assess effects of ACC1 loss in 5 different cell types throughout the paper (HAP1, 293T, 3T3, MEFs and primary hepatocytes). We believe this is extremely rigorous.

For ATGL, we used paired sgRNAs and isolated multiple clones; multiple ATGL knockout clones were analyzed together (as biological replicates) throughout the manuscript. This is highly rigorous and requires multiple cell lines to be cultured and assayed simultaneously. We also use two structurally distinct inhibitors: NG-497 and ATGListatin, which phenocopy the ATGL knockouts. The ATGListatin data is not included in the paper but we have added it here for your reference (**Revision Figure 6a-c**). NG-497 (used throughout the manuscript) was recently developed as a human-specific ATGL inhibitor (PMID: 35362954). It is clearly robust in our assays– aside from phenocopying the *ATGL*-KO, it completely blocks TAG breakdown induced by fatty acyl-CoA starvation (**Fig. 4j**). The chances of independent ATGL knockout clones and two structurally distinct inhibitors all having the same effect is exceedingly low.

Similarly, we showed ACSL loss-of-function has the same effect as ACC1 loss of function by employing two structurally distinct ACSL inhibitors and a CRISPR double-knockout of ACSL1 and ACSL3 (**Fig. 3c**). The effects of ACAT1/2 loss were assessed using CRISPR knockouts (three independent clones) and a well-established small molecule inhibitor (PMID: 6693397, PMID: 36695568). ACAT1 was also found in our unbiased random insertion mutagenesis screen to have an effect on PM cholesterol transport (and was less effective at blocking this response than ACC1 loss, consistent with our follow up assays). We confirmed that both the ACAT1/2 double knockout and the ACAT1/2 inhibitor were robust by measuring cholesterol ester levels. The HMGCR inhibitor Lovastatin has been used extensively in humans since its FDA approval in 1987. We feel we have done an exceedingly diligent job in rigorously testing the importance of all key proteins involved in our study.

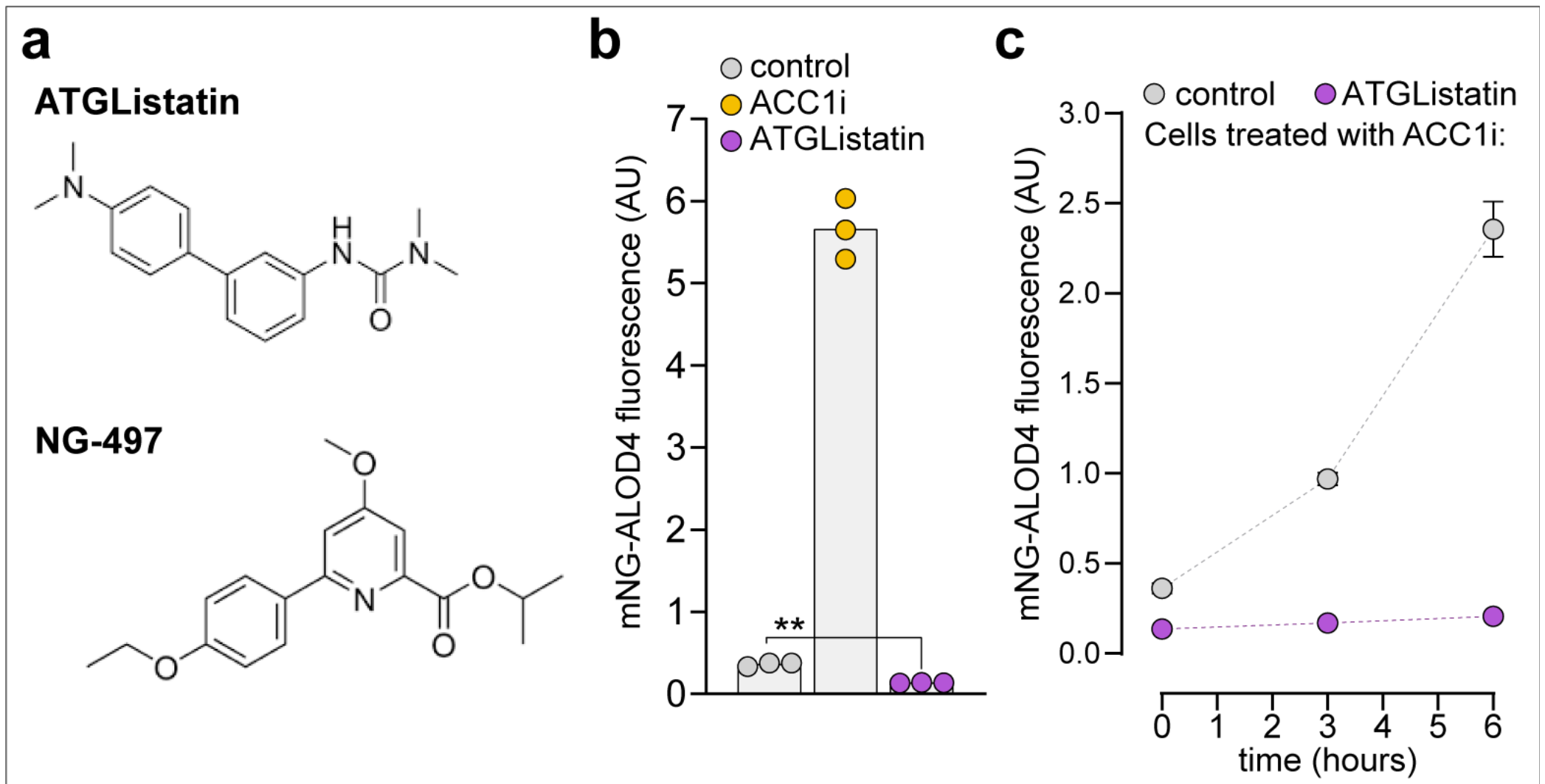

Revison Figure 6. a Structures of ATGListatin and NG-497. **b** Flow cytometry measurement of PM accessible cholesterol after a 16-hour treatment with 10 μM ATGListatin in HAP1 cells (compare to main Figure 3g showing the effects of NG-497). **c** Flow cytometry measurement showing a 16-hour pre-treatment with ATGListatin blocks the effects of ACC1 inhibition on PM accessible cholesterol (compare to main Figure 3e, f).

2c. Statistical analysis needs improved rigor as well. Currently several datasets appear to have been over-interpreted. For instance, in datasets with two experimental variables, two-way ANOVA [followed by appropriate corrected t-test (where applicable)] is a go-to statistical treatment approach (as opposed to one-way ANOVA or t-test with Welch corrections that the authors used). Just to point out one-of-many examples, bar 2 and 4 in Fig.1J – likely are different, but the conclusion is that ACC1 inhibition does not raise PM Cholesterol levels through a mechanism involving NPC1 (?).

We urge the authors to please redouble their efforts in re-checking / correcting all the statistical analyses and data interpretations / conclusions made.

We agree that a two-way ANOVA is a more appropriate analysis for datasets involving two experimental variables, and have addressed this in full throughout the text. This re-analysis of the data did not change the conclusions made. The vast majority of the experimental data in this paper shows large (>5-fold) changes with small variances.

Re-analysis of previous Fig. 1J (now **Extended data Fig. 2b**), using a two-way ANOVA followed by a Sidak correction showed that, as you predict, WT and *NPC1*-KO cells treated with ACC1 inhibitor are different. We previously did not show this comparison because what is more telling than the absolute PM cholesterol levels in these two cell lines is their fold-responsiveness to the inhibitor treatment. WT and *NPC1*-KO cells show a similar fold change in response to the ACC1 inhibitor, which demonstrates that *NPC1*-KO cells are susceptible to PM cholesterol changes caused by ACC1 inhibition. This conclusion is supported by the results of our unbiased screen, which identified that ACC1 loss elevates PM cholesterol in cells lacking NPC1 function (**Extended data Fig. 2e**). Put another way, if cells require NPC1 to show PM cholesterol changes in response to ACC1 inhibition, then *NPC1*-KO cells will be unresponsive to ACC1 inhibition. Instead, we see through multiple approaches (different cell types, different CRISPR knockout lines, and a genetic screen) that ACC1 loss is capable of changing PM cholesterol in the absence of NPC1 function.

We appreciate your suggestion to improve our statistical rigor, and have done this throughout the manuscript. We note again that these new statistical tests did not change the key conclusions of this manuscript. The changed *p*-values from the main figures are summarized here:

a. **Fig. 1d**: the four hour time point has changed from not significant (ns) to a *p*-value of 0.0415. This does not change our interpretation: LXR inhibition does not prevent PM-cholesterol changes induced by 25HC.
b. **Fig. 1h** and **Fig. 1i**: all comparisons are now **** with a $p<0.0001$. This further supports our conclusion.
c. **Fig. 2e**: 1.3 µg/mL PFO* and 3.2 µg/mL PFO* are more significant with $p<0.0001$. This further supports our conclusion.
d. **Fig. 2h**: WT cells -/+ 25HC *p*-value has changed from $p=0.0061$ to p=0.0003. This further supports our conclusion.
e. **Fig. 2j**: all comparisons are now **** with a $p<0.0001$. This further supports our conclusion.
f. **Fig. 3d**: all comparisons are now **** with a $p<0.0001$. This further supports our conclusion.
g. **Fig. 3e**: the *p*-value for the 4 hour treatment has changed from $p=0.0511$ to $p=0.0002$ and the 6 hour treatment *p*-value has changed from $p=0.0121$ to $p<0.0001$. This further supports our conclusion.
h. **Fig 3f**: the *p*-value for the 2 hour treatment has changed from $p=0.0257$ to $p=0.1119$, the *p*-value for the 4 hour treatment has changed from $p=0.0079$ to $p<0.0001$ and the *p*-value for the 6-hour treatment has changed from $p=0.0138$ to $p<0.0001$. While the 2 hour *p*-value is now not significant, the 4 and 6 hour *p*-values have increased in significance, which supports our conclusion.
i. **Fig. 4g**: the *p*-value has changed from $p=0.0362$ to $p=0.0006$. This further supports our conclusion.

2d. Related to point (c) above, the choice of statistical treatment overall seems rather arbitrary, non-standard, or occasionally, invalid. Decision tree for making such calls (i.e., the choice of stat test and why), should be provided, after revisiting all the analyses / correcting the types of treatment, and where relevant, standardizing the choice of tests used.

Thank you for your suggestion. We have added a new paragraph to the Methods section on “Statistical analysis and Schematic generation” which describes our full logic for deciding which statistical test is appropriate.

This paragraph states: “To determine which statistical test to use we used the following decision tree: (**1**) are only two datasets being compared or (**2**) are more than two datasets compared? If two datasets were compared (**1**), we used a Welch’s t-test; this is the recommended approach to compare two independent groups and it is robust when the variance is unequal. If more than two datasets were compared (***2***), we assessed if a single variable was compared across multiple groups (***2a***- e.g. one cell line with different

treatments, comparing untreated to each treatment) or if multiple independent variables were compared (***2b***-e.g., treatment and genotype or treatment and time). When a single variable was compared across multiple groups (***2a***), a one-way ANOVA was used because this test is commonly recommended- it is robust with small sample sizes and does not require equal variances; additionally, by comparing multiple groups simultaneously it prevents false positives that could occur with multiple pairwise t-tests. The one-way ANOVA tests were followed by a Sidak correction, which is recommended when making specific pre-determined pairwise comparisons (as opposed to all possible comparisons, which were rarely made in the manuscript): it provides a balance between protection against false positives and retaining statistical power. In one exception, **Figure 2h**, a Brown-Forsythe one-way ANOVA was used because this dataset had clearly unequal variances and this test is more robust when the variance is highly unequal. Finally, a two-way ANOVA was used for all comparisons involving multiple unique categorical factors (***2b***), which is ideal to determine how different variables work together to elicit an outcome. Two-way ANOVAs were followed by a Sidak correction which reduces false positives while maintaining statistical power."

3. On the proposed mechanism: ACC1/ACSL Inhibition Drives LD Catabolism via ATGL

3a. The manuscript claims that the deficiency in ACC1 induces LD catabolism via ATGL, with key data in Figure 4e and 4f. The mechanism via which ACC1 inhibits ATGL remains highly speculative (based on the data presented thus far, this is likely an overstatement). First, as it stands, the paper has no direct evidence to show for instance inhibitory interaction between ACC1 and ATGL. Regulation of LD levels involves multiple pathways and proteins, rendering the claimed link between ACC1 from fatty-acid synthesis and ATGL in TAG degradation, remains unsubstantiated. Direct experimental validations with orthogonal approaches are necessary to sure up this proposed link, as well as to provide a clear underlying molecular mechanism.

Thank you for your proposed revisions. We agree that a critical piece of data (also noted by Reviewer 2) is that LD loss induced by ACC1 inhibition is ATGL dependent (**Fig. 4g**,**h**). This shows that ATGL is the downstream effector of LD breakdown; without ATGL, ACC1 has no effect on LD levels, which strongly suggests that ACC1 inhibition triggers ATGL-mediated LD catabolism. In the revised manuscript we provide evidence that ACC1 inhibition lowers fatty acyl-CoA pools, which results in ATGL activation. This is supported by in vitro data (PMID: 24440819) which clearly shows that ATGL activity is sensitive to fatty acyl-CoA pools. Since ACC1 is the rate limiting enzyme in fatty acid biosynthesis, it is logical that ACC1 loss will cause fatty acyl-CoA depletion. Consistent with the model that ACC1 inhibits ATGL by depleting fatty acyl-CoAs are the following findings:
(1) ACSL knockout or inhibition phenocopies ACC1 loss, which shows that fatty acyl-CoAs (not fatty acids) are sensed.
(2) The effects of ACC1 loss are reversed by the addition of fatty acids. In contrast, ACSL loss cannot be reversed by fatty acids because ACSLs are required to convert fatty acids into fatty acyl-CoAs.
(3) The effects of ACC1 loss are diminished in ATGL knockout or inhibited cells, showing that ATGL is downstream.
(4) ACC1 inhibition triggers robust TAG loss (now assessed with mass spectrometry), and this is reversed by blocking ATGL (**Fig. 4i**,**j**).
(5) We now include global mass spectrometry data which shows that ATGL inhibition has dramatic effects on DAG, PC and PE saturation. ACC1 inhibition shows a strikingly similar but mirrored/opposite effect, which strongly argues that ACC1 inhibition triggers ATGL activation.

Altogether, we believe we have added significant experimental data that shows that ACC1 inhibition impacts ATGL activation.

3b. LD amount/levels affecting PM cholesterol is convincing based on the data presented thus far. Hence, in the absence of substantial direct inhibition mechanism that supports the authors' model, one possibility is that LD only plays a cargo role to transport cholesterol from PM. Indeed, there are numerous other sources/pathways that lead to fatty acid accumulation that enables LD formation even when ACC1 is deficient; and active ATGL could serve to suppress the LDs because ACC1-dependent primary flux is inhibited, but inhibited ATGL does not have such an ability. The authors should thus also rule out additional possibilities that could result in the same phenotypic changes.

We appreciate your comment and agree that there are multiple mechanisms that could influence LD abundance. In our updated manuscript, we show that LDs are not required for the PM cholesterol changes we observe, showing that they do not play a cargo role in cholesterol transport. We provide evidence that ACC1/ACSL inhibition indirectly activates ATGL by depleting cells of fatty acyl-CoAs. ATGL activation elevates PM cholesterol accessibility, and ATGL inhibition lowers PM cholesterol accessibility. A previous paper elegantly reported that ATGL activity is blocked by long-chain fatty acyl-CoAs (PMID: 24440819). The intellectual advance made by our manuscript was to show a clear link between ACC1/ACSL activity and ATGL activity. We believe it is important to make this clear link in the literature, which does not exist.

3c. Addressing the direct molecular mechanisms (mentioned in (a) above) is absolutely crucial before this work can be published. Since LD regulation is of multifaceted network with manifold interdependent players and feedforward/feedback loops (notwithstanding numerous yet-unknowns), only relying solely upon KO/KD and inhibitor treatments may not give us the full truth. To that end, using cleaner biochemical assays involving purified enzymes beyond as cell-based experiments, could greatly assist the authors, and eventual impact and validity of this otherwise very interesting work.

We are aware that our manuscript previously lacked the mechanism connecting ACC1 inhibition to ATGL activation; it was the focus of an entire paragraph of our discussion section. We believe we have addressed this– please see “Major new findings section.” We show in many assays that ATGL activity is critical for the PM cholesterol phenotype we observe. We also show that ATGL activity is modulated by fatty acyl-CoA abundances through many different approaches. While we agree that in vitro systems are highly elegant, this previous publication (PMID: 24440819) showed that purified ATGL is sensitive to fatty acyl-CoAs. Additionally, in vitro work previously showed that cholesterol accessibility is sensitive to phospholipid acyl chain saturation (PMID: 24138860, 24000774). We believe that our cellular analysis provides a richer picture (benefiting from the multifaceted network of interdependent players) of how effects would play out in vivo. We believe it is important to show that these effects are observed in an in vivo cellular context. We maintain that our manuscript has made multiple contributions that constitute a large and impactful publishable unit of information.
Key contributions that warrant publishing this manuscript without in vitro data:

1. We show that ACC1 inhibition (a strategy tested in humans to combat metabolic syndromes) has the unexpected consequence of raising PM cholesterol accessibility (**Fig. 2a-e**).
2. We show that ACC1 inhibition blocks the interferon-stimulated immune signaling cascade triggered by the enzyme CH25H. ACC1 inhibited cells cannot deplete their PM cholesterol in response to 25HC (**Fig. 1**).
3. We show that ACC1 acts independently from ACAT1 and ACAT2 in 25HC-induced cholesterol responses. It also has a much more dramatic effect, which contrasts previous reports that ACAT1/2 are essential to this pathway (PMID: 36695568) (**Fig. 2g-j**) .
4. We show that ACC1 and ACSL loss result in ATGL activation by depleting fatty acyl-CoA pools. This results in LD catabolism (a highly logical strategy for cells to restore fatty acyl-CoA pools). This connection needs to be clearly made in the literature (**Fig. 3c, Fig. 3h,** and **Figure 4**).
5. We show that ATGL has activity beyond TAG hydrolysis by generating a cell line that is highly depleted of TAGs (**Figure 5**).
6. We show that ATGL activity modulates DAG, PC and PE saturation, which changes membrane fluidity (**Figure 6**).
7. We show that cholesterol accessibility can be dramatically altered through sphingomyelin-independent mechanisms (all Figures, and notably **Fig. 2f**).
8. We challenge the model that cholesterol accessibility always correlates with cholesterol extractability, which has serious implications for how accessible cholesterol is currently defined by an entire field (**Fig. 7a-c**).
9. We show that ACC1 and ACSL loss (unexpectedly) causes dramatically elevated cholesterol biosynthesis with similar effect sizes to a statin (**Fig. 7h**, and in vivo **Fig. 7k**).

3d. Could oleic acid (OA) addition rescue the amount of LD in ATGL depleted or inhibited background, while also assessing the amount of the PM cholesterol?

We thank the reviewer for this suggestion. We interpret this question as asking whether oleic acid (OA) can modulate LD abundance and PM cholesterol under conditions of altered ATGL activity. We show that LDs are not required for the PM cholesterol phenotype we observe in this manuscript (**Figure 5**). In concordance with previous publications, we believe that OA modulates ATGL activity (PMID: 24440819), which we show has consequences on PM cholesterol accessibility in the absence of LDs. For example, OA and palmitic acid (PA) reverse the effects of ATGL activation triggered by ACC1 inhibition (**Fig. 3h**, **Extended data Fig. 5g**, and **Extended data Fig. 7c**– this data is in the absence of LDs). Additionally, we show that PM accessible cholesterol is not altered by OA in the absence of ATGL (**Fig. 3i**). Altogether, this builds a model that OA addition reduces PM accessible cholesterol by inhibiting ATGL.

3e. ACSL should also be investigated further since ACSL knockdown/inhibition showed similar results to ACC1 deficiency. The authors discussed the crosstalk between ACSL and ATGL in Figure 5a-c, which show that the LD amount is directly related to the PM cholesterol level. This somewhat diminishes the importance and uniqueness of ACC1. It could be argued, based on the presented data, that the processes of fatty acid activation or TAG synthesis may play a more central role instead of ACC1. ATGL and ACSL KO should also be assessed, as generically mentioned above.

Thank you for your assessment. We previously showed that ACC1 and ACSL loss are phenotypically identical. We also showed that they act in the same pathway using an epistasis experiment: combining ACC1 and ACSL inhibitors together did not have an additive effect (previous **Fig. 3i**). We agree fully that *ACC1*-KO and *ACSL*-KO have the same effects on ATGL (and PM cholesterol) (**Figure 3**). We do not believe this diminishes the role of ACC1, rather it has revealed a central mechanism behind our work: long-chain fatty acyl-CoA depletion (via ACC1, FASN or ACSL loss) causes ATGL activation, and ATGL is the critical downstream effector of the PM cholesterol changes we observe.

3f. Figure 4d and Figure 5b,g show that LD sizes and LD counts are altered in two ATGL-deficient cases. We wonder if there is a way to calculate and discuss the entire LD content level, such as area x numbers (by imaging, or other flow-based or biochemical assays). Such an analysis measuring the total amount of lipids, the total amount of cholesterol, and CEs would provide a more holistic view on phenotypic changes. It is also quite challenging / impossible to discern from the presented data / figure legends, the corresponding sample size / particle counts made (e.g., no. of LDs quantified per cell in each set; no. cell quantified for cholesterol content in each group, etc.)

We appreciate your suggestion and have added the total area (in μm^2) of BODIPY 493/503 signal to **Figure 4h**, which is the most important figure quantifying changes in LD mass. We believe Figure 5b speaks for itself (there are either LDs or not).

We have added a sentence to the figure legend stating how LDs were quantified for each condition (**Fig. 4g** and **Fig. 5c**). Each single data point shows the analysis of ~75 cells. Specifically, the total number of LDs in a field of cells was divided by the total number of nuclei (~75 nuclei per field). The total number of cells analyzed per treatment was thus ~225 cells from three independent fields of view. This is explained in detail in the Methods section “Quantification of LDs.”

Other points:
(i) Flow cytometry data – raw data and gating analysis should be shown – these are standards in many journals including this.

We have now included a Supplementary file showing gating strategies for flow cytometry data. The flow cytometry data in this manuscript assesses large changes in a uniform cell population. We are never assessing rare or under-represented cell populations. For all flow cytometry data we gated on living cells and single cells using a forward scatter (area) vs forward scatter (height) plot, which allowed for a first-pass selection of single and living cells (**Revision Figure 7a**). This gating strategy eliminates small or dead cell debris and also doublet cells. This population was then gated a second time with a forward scatter vs side scatter plot, which ensured only cells of a similar granularity were analyzed (**Revision Figure 7b**).

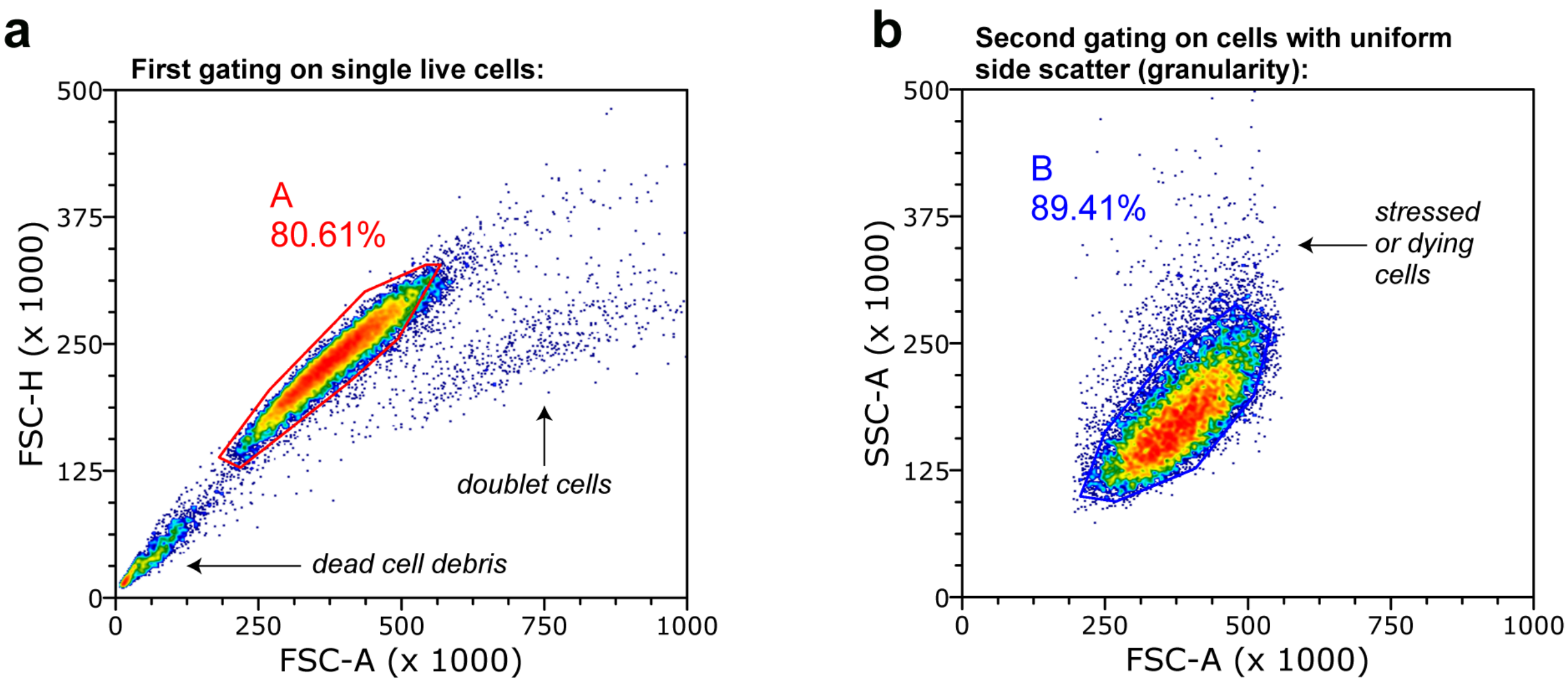

Revision Figure 7. a Flow cytometry data for WT HAP1 cells showing a density plot with forward scatter-area on the x-axis and forward scatter-height on the y-axis. The gated cell population is circled in red. **b** The second gated population which is performed on the "A" gated cells, showing forward scatter-area on the x-axis and side scatter-area on the y-axis. Cells that are analyzed for fluorescence are those shown in the blue "B" gate.

(ii) likewise, all blots/gels should be standardly shown in full-view uncropped format as supplementary information.

Thank you for this request. We have now included a document that shows all uncropped Western blots and DNA gels.

(iii) The authors performed TKO, DKO, etc. The supplementary information description for these procedures is insufficient / lacking details.

We have added a new detailed methods section describing how our lab generates CRISPR knockouts (Page 24, Lines 610-632).

(iv) Line 128 - Is ACC1 activity known for the truncated version?

To my knowledge, the activity of ACC1 truncated at exon 52 has not been previously reported. However, it is clearly shown that the C-terminal carboxyltransferase domain of ACC1 makes extensive contacts that support ACC1 dimerization, and ACC1 dimerization is required for its function (PMID: 29899443). We believe that the ACC1 exon 52 truncation is fully inactive for multiple reasons. First, ACC1 truncated at exon 52 was not detectable by Western blot, suggesting that this protein is either degraded or that the mRNA is subjected to nonsense mediated decay (**Extended data Figure 1m**). Second, ACC1 truncated at exon 11 lacks both enzyme active sites, and this truncation behaved identically to ACC1 truncated at exon 52 (**Extended data Figure 1k**). Finally, cells harboring an ACC1 exon 52 truncation were not responsive to Firsocostat treatment (which blocks ACC1 dimerization), showing that this protein is not active (**Extended data Figure 1l**).

(v) Line 187 - The differences in data Fig. 2f and Fig. 1k should be compared in one single plot and with adjusted comparison.

The sentence you are referring to is: "ACAT1/2 inhibition reduced PM cholesterol depletion induced by 25HC, however the magnitude of the effect was small compared to previous reports and also less than what was observed for ACC1 inhibition (previous text- **Extended data Fig. 2f and Fig. 1k**)." We agree that if we claim that the effect of ACC1 loss is more dramatic than ACAT1/2 loss, we should compare these in one plot. We do

this in **Fig. 2j** and **Extended data Fig. 4d**. In the new manuscript, we avoid claiming that one effect is stronger than the other (despite clear differences). Instead, we focus on how the combination of ACAT1/2 and ACC1 loss (losing all three proteins by various mechanisms) is additive compared to the loss of either ACAT1/2 or ACC1 alone. The revised text states: "If ACC1 and ACAT1/2 are acting in the same pathway to regulate 25HC-induced cholesterol depletion, then knocking out ACC1 and ACAT1/2 together should not have a combinatorial effect. We treated *ACAT1/2*-DKO cells with ACC1 inhibitor, which caused a dramatic increase in PM cholesterol in the presence of 25HC (**Extended data Fig. 4d**). Additionally, ACAT1/2 inhibition with the inhibitor SZ58-035 further blunted the effect seen in *ACC1*-KO cells (**Extended data Fig. 4e-g**)[43]. These findings were further supported by a triple knockout cell line lacking ACC1, ACAT1 and ACAT2 (**Fig. 2j** and **Extended data Fig. 4h**). These epistasis experiments cleanly demonstrate that ACC1 acts through a distinct pathway from the ACAT1/2 proteins, both at baseline and in response to 25HC."

(vi) Figure 2n - The first bar (WT basal) and the third bar (ACC1 KO basal) should be compared. (As requested above, please use the correct type of statistical treatment).

We have added a comparison between the first and third bar in this experiment (which is now **Extended data Fig. 4b**), which is not statistically significant, with p=0.8909. This analysis was made using a two-way ANOVA test followed by a Sidak correction because it compares two independent variables (two cell types and two treatments). We have made appropriate changes to all statistical analyses in this manuscript.

(vii) Line 225 - The more common format is BODIPY 493/503

Thank you for your comment. We have now changed all instances of "Bodipy 493/503" to "BODIPY 493/503."

(viii) As described, CRISPR KO of ACC1 at various exons cause multiple phenotypes: please specify the specific exons being targeted when discussing the ACC1 KO datasets in subsequent paragraphs.

Truncation of ACC1 at different exons does not cause multiple phenotypes. We show that introducing CRISPR lesions at exon 11, 21, 31, 41 and 52 all have the same effect (**Extended data Fig. 1k**). We addressed this in more detail in response to your comment (*iv*). ACC1 truncated at exon 3 was lethal to cells (we were unable to isolate any clones), and we therefore did not analyze these. We do not investigate the exon 3 deletion further, but we cite a manuscript (PMID: 31155494), which shows that exons 2-4 of ACC1 are spliced together to generate a circular RNA that regulates AMPK, and is important for cell viability. To improve clarity, we added this sentence to the main text: "Using CRISPR to generate a series of ACC1 truncations, we found that an exon 52 truncation behaves identically to an exon 11 truncation lacking both catalytic domains. Furthermore, the exon 52 truncation was not detectable by Western blot and it was not responsive to the ACC1 inhibitor Firsocostat, a drug tested in human clinical trials for the treatment of metabolic dysfunction-associated steatotic liver disease (MASLD), demonstrating that it lacks protein function (**Extended data Fig. 1k-m**). Throughout this manuscript, we utilize cell lines with ACC1 truncated at exon 52 for all assays, and we refer to these as *ACC1*-KO cells."

(ix) In lines 266 – 267, ….stimulates LD catabolism by starving the cell of fatty acyl-CoA. I've not been able to find the evidence in the manuscript or references.

This is stated in the following manuscript: PMID: 37494933. We also would like to point out this manuscript (PMID: 24440819) which shows that ATGL is inhibited by long-chain fatty acyl-CoAs.

Reviewer #4 (Remarks to the Author):

I co-reviewed this manuscript with one of the reviewers who provided the listed reports. This is part of the Nature Communications initiative to facilitate training in peer review and to provide appropriate recognition for Early Career Researchers who co-review manuscripts.

Nature Communications manuscript

Dear Reviewers,

We greatly appreciate the opportunity to submit a revision of our manuscript. Based on your feedback, we have not added additional mechanistic data to the manuscript. We have addressed all technical concerns and improved the data interpretation as requested. Below please find the reviewer comments in blue, and our responses in black. All quoted text copied from the revised manuscript is shown with a light yellow highlight.

REVIEWER COMMENTS

Reviewer #1 (Remarks to the Author):

The authors have done substantial work in response to my first round, and I want to acknowledge that. The TAG-TKO cell line is elegant, the lipidomics is thorough, and abandoning the LD-buffering model when their data didn't support it reflects scientific integrity I respect. The new mechanistic spine - fatty acyl-CoA depletion activates ATGL, which remodels phospholipid saturation, which alters PM cholesterol accessibility - is more coherent than what they had before.
That said, they've replaced one overreaching story with another. The new manuscript leans on a mechanism the authors themselves admit they can't explain, frames a generalizable biophysical principle as if it were specifically about ACC1, contains a paradox they handwave away, and silently dropped negative data that should have stayed in.

Major concerns:
1. The central mechanistic claim is unsupported by direct evidence. The headline finding of this revision is that ATGL remodels DAGs toward polyunsaturated species independently of TAG hydrolysis. Everything downstream depends on this. And in their discussion, the authors write that "future work will be needed to determine how ATGL remodels the cellular DAG pool." That sentence is a confession. You don't get to defer the central claim of your paper to future work. The simplest experiment that would resolve this is a catalytic-dead ATGL rescue in the ATGL-KO + ACC1i background. If catalytically inactive ATGL restores PC/PE unsaturation, the activity is allosteric or scaffolding; if it doesn't, ATGL's catalysis is required, and the question becomes against what substrate, given that there is essentially no TAG in TAG-TKO cells. The transacylase hypothesis is suggestive but isn't evidence in this system. One clean experiment would settle it.

We greatly appreciate your thoughtful consideration of our model and for suggesting follow up experiments. At least four previous studies elegantly show that ATGL has transacylase activity on DAG (PMID: 34543623, 35676490, 31035700, 15364929), which demonstrates that ATGL can remodel lipids beyond TAG. While we do not have catalytic dead point mutations in ATGL reintroduced into ATGL knockout cells, we would like to note that the ATGL inhibitor used in the study, NG-497, is an orthosteric (competitive) inhibitor of ATGL. This suggests that ATGL's catalytic activity is indeed required for the PM changes we observe. However, like you mention, a point mutation would be more solid evidence since it is possible that this inhibitor induces allosteric changes in ATGL that disrupts its non-catalytic function.

To the best of knowledge, this is the first report showing how ATGL activity can modulate the phospholipid composition of cells. We show the timescales and outcome of ACC1, FASN and ACSL inhibition on ATGL-mediated lipid remodeling, which was previously not reported. Finally, we show that ATGL activity impacts cellular cholesterol homeostasis, which is a surprising discovery linking fatty acid metabolism to

cholesterol homeostasis. We have completely re-written the discussion section (quoted in response to your comments below) of our manuscript to frame our findings more clearly.

2. ACC1 is no longer the protagonist of this story. ACSL inhibition phenocopies ACC1 inhibition. Palmitic acid, which doesn't require ACC1, rescues it. FASN-KO does the same thing given enough time. The biologically relevant variable is fatty acyl-CoA availability, and ACC1 is one of several enzymes whose inhibition depletes it. That's a fine finding, but it isn't an "ACC1 inhibits ATGL" finding, which is what the title still says. I anticipate the response will be that ACC1 was the top screen hit, that Firsocostat is in clinical development, and that the dLKO model is ACC1/ACC2-specific, which are all true. But the screen and the translational angle don't change the fact that the mechanism, as the authors describe it, has nothing specifically to do with ACC1. The honest framing is that the screen identified ACC1 as a tractable entry point into a general fatty acyl-CoA/ATGL axis. The title and abstract should reflect the mechanism, not the screen hit.

We agree that ACC1 is no longer the protagonist. We have changed the title and abstract to reflect this. Figures 3-7 all point to the main model of fatty acyl-CoA abundance modulating ATGL activity. Additionally, we have changed the discussion section to focus more on this topic. For example, we have added these two paragraphs (new addition in yellow highlight):

“In this study we set out to find new regulators of cholesterol accessibility at the PM. We were surprised to discover that ACC1, a protein extensively studied for its role in fatty acid synthesis, also impacts cellular cholesterol homeostasis. ACC1 knockout raises PM accessible cholesterol levels 5- to 10-fold in cells and a mouse model; additionally, ACC1 loss blocks PM cholesterol depletion induced by 25HC through a mechanism that is independent of cholesterol esterification by ACAT1/2. Inhibition or knockout of the ACSL proteins, which charge fatty acids with CoA, phenocopies ACC1 loss, indicating that fatty acyl-CoA (the shared metabolic product of ACC1 and the ACSLs) is the relevant signal sensed by this pathway. Long-chain fatty acyl-CoAs were previously shown to allosterically inhibit ATGL in vitro[47]. In this study we used epistasis, pharmacology, and fatty-acid rescue experiments to demonstrate that ATGL senses fatty acyl-CoA abundance in cells; ATGL then acts as a rheostat, converting metabolic information into changes in the physical state of the plasma membrane.

Lipidomics data reveals that ATGL activation elevates polyunsaturated DAG abundance, as well as polyunsaturated PC and PE which we propose are made from DAG precursors. We hypothesize that ATGL generates polyunsaturated DAGs through a transacylation reaction for two reasons. First, ATGL was previously reported to have transacylase activity on DAG both in vitro and in vivo[63–66]. Secondly, we observed dramatic changes in PM cholesterol accessibility with *ACSL1/3*-DKO and ACSL inhibition. ACSL loss blocks the formation of fatty acyl-CoAs; therefore, the polyunsaturated DAG species must be made through a mechanism that does not require fatty acid charging with CoA. This rules out de novo lipid synthesis, and nominates either elevated desaturase activity or a transacylation reaction. Future work will be needed to determine how ATGL remodels the cellular DAG pool, and the consequences of this activity on cell and organismal biology.”

3. The "trapped accessible cholesterol" model conflates two different observables. The authors argue that ATGL activation increases ALOD4 binding while decreasing MβCD extractability, and they call this a paradox resolved by an energetic argument about acyl chain shielding. It isn't a paradox. ALOD4 reports cholesterol's chemical activity at the membrane surface - hydroxyl exposure, recognition by a protein with cooperative kinetics. MβCD extraction reports the rate of monomer dissociation into a hydrophobic acceptor. These are not the same physical property and were never required to track linearly. The data may show something simpler and more interesting: in fluid, polyunsaturated membranes, cholesterol presents differently to a protein probe than to a small-molecule acceptor. That's worth reporting, but it requires the authors to define what they mean by "accessible cholesterol" - the operational definition (binds ALOD4) or the functional one (moves out of the membrane). They cannot have both, and this discordance should be the headline of Figure 7, not buried as a paradox to be waved off.

We appreciate you raising this point, and we agree with your assessment. The reason we reported this as a paradox is that the field has **defined** accessible cholesterol as 1) being the fraction of membrane cholesterol that is easily extractable by acceptors like MBCD, and 2) they claim it can be conveniently measured with ALOD4. We report here that, as you explain, ALOD4 binding is not the same as extraction. This is the first

time, to the best of our knowledge, that these reported measurements have been reported to ***not*** correlate with one another. We sought to gently raise this point without directly calling to question the existing definition of “accessible cholesterol” since we do not believe we have sufficiently (or extensively) demonstrated this concept in this paper. To improve clarity, we have rewritten the Discussion section and suggested caution in interpreting ALOD4 binding as a proxy for cholesterol extractability. We state:

“While pioneering studies of accessible cholesterol defined it as the pool of cholesterol readily extractable by MβCD, we find that ALOD4 binding and cholesterol extractability are not equatable in polyunsaturated membranes. Our data show that these two readouts report distinct physical properties of cholesterol that are not required to track together. ALOD4 binding reflects cholesterol's chemical activity at the membrane surface (its ability to be recognized by a cholesterol-binding protein), while MβCD or protein-mediated extraction instead reflects the kinetics of cholesterol dissociation into a small-molecule or protein acceptor. In support of the latter, our data show that polyunsaturated membranes trap cholesterol at the PM to a degree sufficient to trigger SREBP2 activation, indicating that extraction by intracellular cholesterol-binding proteins is impaired. This distinction has practical consequences for how "accessible cholesterol" is defined and measured across the field: an operational definition (cholesterol that binds ALOD4 or related probes) and a functional one (cholesterol that can be readily transported out of a membrane). Future work relating probe-based accessibility to actual transport and extraction rates across a range of defined membrane compositions will be needed to resolve which property governs cholesterol's biological functions in signaling, transport, and disease.”

4. The AMPK data was silently removed. The original submission contained AMPK data implicating AMPK in the regulatory axis. The rebuttal explains it's gone - AMPK1/2-DKO lethal, AMPK2-KO no effect, AMPK1 siRNA ambiguous. But the manuscript contains no trace. Negative results that rule out a plausible mechanism belong in the manuscript, not in a rebuttal letter readers won't see. Add a supplementary figure and a sentence in discussion. Without this, removal of data that was in the original submission looks like selective reporting, even if it isn't.

We agree and we have now added the prior AMPK data and the data gathered in response to Reviewer 3 (revision 1), to a new **Extended data figure 7** (shown below). We mention this data in text lines 289- 298. We state:
“To modulate ATGL activity in an orthogonal way, we targeted AMP-activated protein kinase (AMPK), a master regulator of cellular energy metabolism. AMPK regulates the activity of both ATGL and ACC1 through an activating and inactivating phosphorylation, respectively (**Extended data Fig. 7a**). Consistent with our previous data, inhibition of AMPK with the drug Dorsomorphin (also called Compound C), which is expected to inhibit ATGL and activate ACC1, elevated LD numbers and lowered PM accessible cholesterol (**Extended data Fig. 7b-g**). Testing additional structurally distinctive AMPK inhibitors lowered PM accessible cholesterol to various extents (**Extended data Fig. h-l**). However, this effect was not recapitulated in cells where the AMPK isoforms AMPK1 and AMPK2 were genetically ablated with siRNA and CRISPR knockout, respectively. Given the variability of this assay (and the reliance on small molecule inhibitors), we did not further explore this line of investigation.”

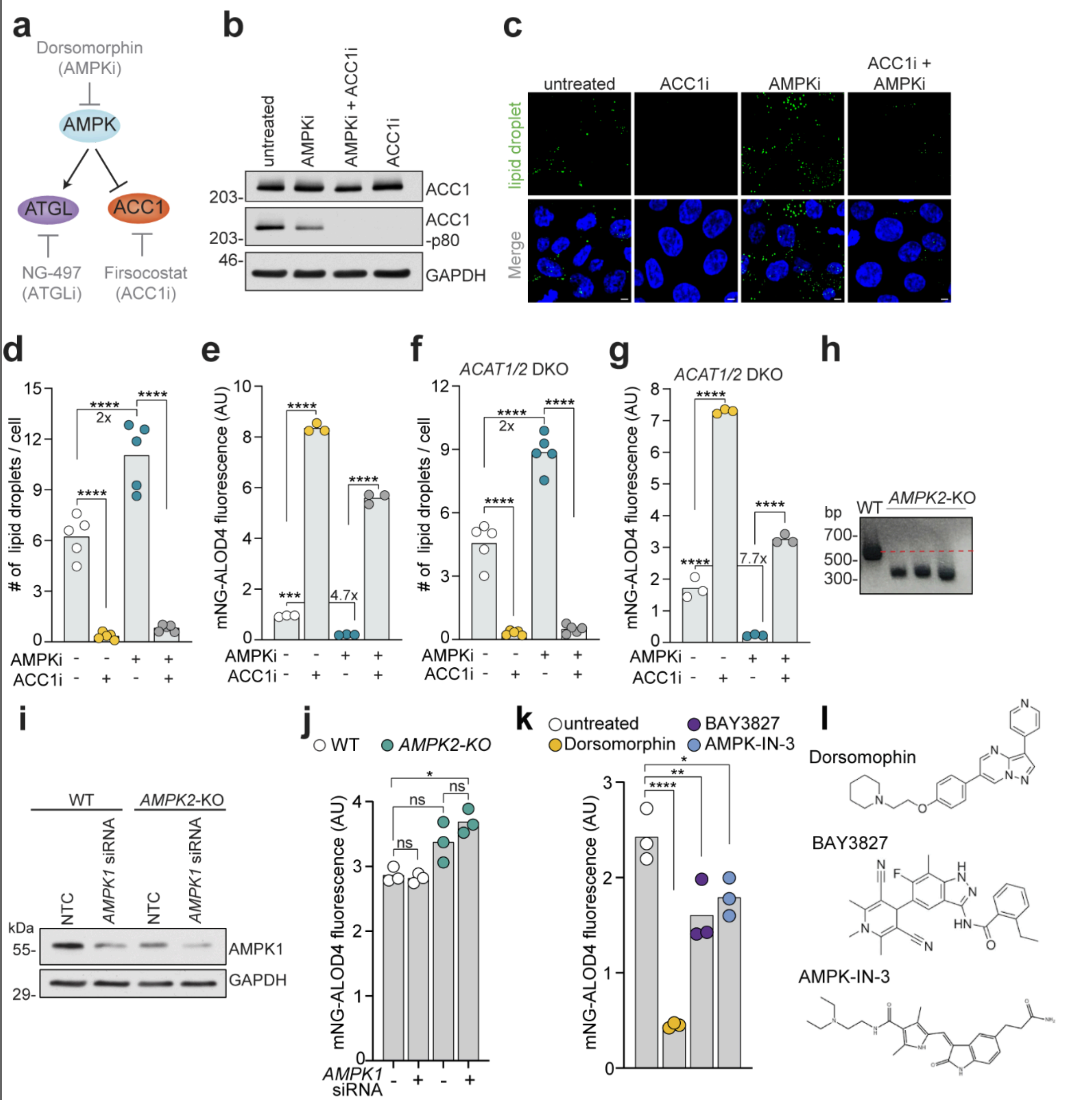

Extended data Figure 7. a Schematic showing that AMPK activates ATGL and inactivates ACC1. Inhibitors for each enzyme are shown in gray. **b** Western blot analysis of ACC1 phosphorylation in WT HAP1 cells left untreated or treated with AMPKi (2.5 μM Dorsomorphin), ACC1i (30 μM Firsocostat) or both. **c**-**e** WT HAP1 cells were left untreated or treated with ACC1i, AMPKi or both and then analyzed by microscopy to quantify LD numbers per cell (**c**, **d**) or by mNG-ALOD4 flow cytometry analysis to determine PM cholesterol levels (**e**); the scale bar is 5 microns. **f**,**g** LD quantification (**f**) and flow cytometry analysis of PM cholesterol using mNG-ALOD4 staining (**g**) in *ACAT1/2*-DKO cells left untreated or treated with AMPKi, ACC1i or both. **h** PCR analysis of *AMPK2* knockout (KO) clones. **i** Western blot analysis showing siRNA knockdown of AMPK1 or a non-targeting control (NTC) in WT and *AMPK2*-KO cells. **j** Flow cytometry analysis of PM cholesterol in WT or *AMPK2*-KO cells with or without AMPK1 siRNA or a NTC. **k** Flow cytometry of PM accessible cholesterol levels after cells were treated for 20 hours with three distinct AMPK inhibitors (2.5 μM Dorsomorphin, 5 μM BAY3827 and 10 μM AMPK-IN-3). **l** The structure of each AMPK inhibitor. All cells were grown in lipoprotein depleted serum media for 16 hours prior to drug treatments. Each data point is the total number of LDs per cell in a single field of view (**d**,**f**). Statistical significance was determined by a one-way ANOVA with a Sidak correction (**d**-**g**), a two-way ANOVA with a Sidak correction (**j**) or by a Dunnett's one-way ANOVA (**k**). Exact *p*-values in are: (**d**) untreated vs ACC1i $p<0.0001$, untreated vs AMPKi $p<0.0001$, AMPKi vs ACC1i + AMPKi $p<0.0001$; (**e**) untreated vs ACC1i $p<0.0001$, untreated vs AMPKi $p=0.0005$, and AMPKi vs ACC1i + AMPKi $p<0.0001$; (**f**) *ACAT1/2*-DKO untreated vs ACC1i $p<0.0001$, untreated vs AMPKi $p<0.0001$, and AMPKi vs ACC1i+ AMPKi $p<0.0001$; (**g**), *ACAT1/2*-DKO untreated vs ACC1i $p<0.0001$, untreated vs AMPKi $p<0.0001$, and AMPKi vs ACC1i + AMPKi $p<0.0001$; (**j**) WT NTC siRNA vs AMPK1 siRNA $p=0.9999$, WT vs *AMPK2*-KO (NTC siRNA) $p=0.0967$, WT (NTC siRNA) vs *AMPK2*-KO with AMPK1 siRNA $p=0.0110$, and *AMPK2*-KO cells NTC vs AMPK1 siRNA $p=0.4357$; (**k**) untreated vs Dorsomorpin $p<0.0001$, untreated vs BAY3827 $p=0.0081$, and untreated vs AMPK-IN-3 $p=0.0319$. AU is arbitrary units.

5. The actual contribution is being oversold. The principle that PUFA-rich membranes disrupt cholesterol-acyl chain packing is well established in vitro. What's novel here is how this principle is regulated in living cells, by which enzymes, on what timescale. The discussion frames the finding as a discovery about ACC1 and ATGL

specifically and obscures the broader biophysical principle the authors have actually demonstrated. A more accurate framing would strengthen the paper, not weaken it.

Our results section where this data first appears clearly states that we are aware of this established concept. In lines 361-364 we say: "Previous work demonstrated that phospholipid saturation directly modulates cholesterol accessibility[53–55]. Intuitively, because cholesterol is a planar molecule, it makes van der Waals interactions more robustly with saturated fatty acyl chains. We thus hypothesized that ATGL activation elevates PM cholesterol accessibility by elevating PC/PE unsaturation.

We appreciate your comment about reframing the Discussion section, and we agree that it strengthens the manuscript. We have added:

"Cholesterol accessibility is fundamentally a biophysical property governed by the strength of the interaction between cholesterol and the surrounding phospholipids and sphingolipids: saturated acyl chains sequester cholesterol efficiently, while polyunsaturated chains pack poorly and leave cholesterol more chemically exposed[67,68]. Consistent with this principle, we find that increased PC and PE unsaturation elevates PM fluidity and cholesterol accessibility. This relationship is well established in reconstituted systems such as liposomes and giant unilamellar vesicles, but has been difficult to test as a regulated, physiological process in cells. Our data suggests that a cell can dial PM cholesterol accessibility up or down by shifting the acyl-chain saturation of PC and PE, and that ATGL can drive this shift bidirectionally. To the best of our knowledge, this is the clearest demonstration that endogenous, physiologically triggered remodeling of bulk phospholipid saturation is sufficient to reset the chemical activity of PM cholesterol in intact cellular membranes.

The kinetics of PM accessible cholesterol changes occur on the timescales of minutes to a few hours after ATGL activation or inhibition (**Fig. 3b**, **j**), which is far faster than the transcriptional programs classically invoked for membrane adaptation. This pathway is thus mechanistically distinct from the transcriptional feedback loops that implement homeoviscous adaptation in response to temperature[69–71]. Here, membrane fluidity is instead tuned post-translationally on the timescale of metabolic fluctuation rather than gene expression."

Minor points:

6. The Kennedy pathway connection is supported by acyl chain signature matching but not by direct flux measurement; isotopic labeling would demonstrate it and would also strengthen the response to point 1.

We agree that isotopic labeling would strengthen our data. Our lab is not equipped for radioactivity based measurements, and because the Editors do not require additional mechanistic insights, we have not performed this experiment.

7. The new mechanistic findings are essentially HAP1-only - repeating Laurdan and DAG measurements in one additional cell line would help.

We agree this would help. However, our data, and many in vitro studies as you discuss in point #5, demonstrate that cholesterol accessibility is a direct reflection of membrane fluidity, and we have demonstrated that cholesterol accessibility changes in HAP1, 3T3, 293T, MEFs and primary hepatocytes.

8. Several lipid classes (PG, plasmalogens, LPE) change with ACC1 inhibition but don't reverse with ATGL inhibition, meaning ACC1 inhibition has substantial ATGL-independent lipidomic effects that receive no discussion; this should be acknowledged honestly rather than dismissed.

We agree, and have added to our section discussing the lipidomics data. We state:

"Only DAG, PC and PE saturation mirrored the bidirectional effects of ATGL on PM cholesterol; other major phospholipid classes, including phosphatidylinositol (PI), phosphatidylglycerol (PG), and phosphatidylserine (PS), were not inversely modulated by ATGL activation and inhibition (**Extended data Fig. 8h-j**). Additionally, lysophosphatidylinositol (LPI), lysophosphatidylglycerol (LPG), lysophosphatidylserine (LPS), sphingomyelin (SM), ceramide (CER), hexosylceramide (hex-CER), lactosylceramide (lac-CER) and cholesterol esters (CE) were not inversely modulated by ATGL activation and inhibition (**Extended data Fig. 9a-k**). Lipidomics analysis revealed that both ACC1 and ATGL inhibition independently lowered PG levels, with the combination treatment showing the most dramatic reduction in PG. Conversely, lysophosphatidylcholine (LPC) levels were elevated

by both treatments, and even more so by the inhibitor combination. Plasmalogens were also elevated by combined ACC1 and ATGL inhibitor treatment. Finally, lysophosphatidylethanolamine (LPE) abundance was elevated by ACC1 inhibition, and unaltered by ATGL inhibition. Altogether, the lipidomics data is consistent with DAG, PC and PE causing the PM cholesterol changes observed."

9. In conclusion, the science here is good. The TAG-TKO is clever, the lipidomics resolves much of what I challenged in round one, and the mouse data is appropriate. My objections are mostly about framing, mechanistic completeness on the central new claim, and transparency around AMPK. These are addressable. With a catalytic-dead ATGL experiment, an honest reframing toward phospholipid saturation as the operative mechanism, and restoration of the AMPK negative results, this becomes a strong paper.

Thank you for your assessment. The Editors do not require additional mechanistic data, and we therefore do not add the ATGL catalytic-dead experiment. As mentioned above, our results with NG-497 *suggest* that ATGL catalysis is required for the observed effect, since this small molecule binds competitively in ATGL's active site. We have added all AMPK data back into the manuscript (discussed in response to point #4) to provide a complete and transparent account of these experiments. Finally, we have completely rewritten the Discussion section of the manuscript, and also changed the title and abstract. We have greatly appreciated your input, which has significantly strengthened the paper.

Reviewer #2 (Remarks to the Author):

While this is a highly complex system, I believe that the authors have addressed the major concerns adequately and this work will be of general interest to cell biologists.

Thank you, we appreciate the time and effort spent by each reviewer.

Reviewer #3 (Remarks to the Author):

The authors have made commendable efforts during revisions. The rewriting of the introduction and inclusion of supporting illustrations have made this revised paper much more accessible to broader mechanistic life scientists as well, including those not specialised in lipid maintenance pathways exclusively.

A few additional questions / comments to be addressed are below:

1. What is the rational behind the choice of Sidak correction post one-way ANOVA, as Dunnett is used commonly, at least for many-to-one comparisons - the authors should fix the statement and corresponding plots / re-analyze where applicable. One can also consider Tukey for all possible multiple pairwise comparisons. It is unclear if Sidak should be the go-to for every dataset in this paper following suitable application of one-way ANOVA.

We previously chose the Sidak correction because it was a conservative post-hoc test for one-way or two-way ANOVAs. We have now taken the reviewer's suggestion and re-analyzed data throughout the manuscript. We describe the rationale for our choices in the Methods section on Statistics.
We have added:
"When a single variable was compared across multiple groups (**2**), a one-way ANOVA was used. For all one-way ANOVAs used, we ensured an equal spread of variance with a Brown-Forsythe test. When making an all-to-one comparison (**2a**) (e.g. all treatments to untreated), we used a Dunnett's test post-hoc, otherwise when making comparisons without a clear comparison structure (**2b**), we used a Sidak correction post-hoc. Post-hoc Tukey tests (**2c**) are ideal when every possible comparison is made, which was not used in this manuscript. One experiment had unequal variance spread (**Fig. 2i**); for this experiment, we performed a Welch's ANOVA with a Dunnett T3."

The following statistical tests were updated:
These figures were modified from a one-way ANOVA with a Sidak correction to a Dunnett's ANOVA:
- Extended data Fig. 1k

- Figure 2d
- Extended data Fig. 3c
- Figure 3b
- Figure 3j
- Figure 6l
- Figure 7c
- Extended data Fig. 9e

2. One-way ANOVA classically assumes equal level of spread so statement 'one-way ANOVA' 'does not require equal variances' is limited and requires update/correction.

We appreciate this comment and have changed this section of our Methods, removing this statement completely. Please see the quoted text in response to point 1. To ensure that all one-way ANOVAs used in this manuscript have equal spread, we have performed Brown-Forsythe tests. Only one experiment showed unequal variance (**Fig. 2i**), which was analyzed with an appropriate test: the Dunnet T3 Welch's ANOVA.

3. The authors defended in several instances how ">750 or so publications" have used several of these compounds as inhibitors, implying that usage of these compounds could be warranted or should not be queried. Sadly no end of previously reported publications can rule out off-targets or guarantees specificity if these published studies had not carried out responsible orthogonal validations in identical biological contexts (cell lines, etc.) as in this paper - I would think that the authors are just as aware of numerous cases of misconceptions, incorrect pathway models, mechanisms, conclusions, etc., perpetuated over decades in many fields of life sciences. Thus, although I congratulate the authors for improving the rigor and responsibility in numerous ways through the revisions, I would point out that defending with no. of previous publications dilutes the focus when the soultion exclusively rests on what the authors indeed found in their own orthogonal assay results during revisions. Even clinically approved drugs hit multiple targets.

We appreciate this response and we agree that publication quantity does not ensure drug specificity. We will refrain from using such arguments in the future, and instead always perform experiments such as knockouts to ensure specificity.

I believe that responses to the above comments/queries can be overseen by the handling Editor. I further think that the other reviewers have also asked many relevant and important (often subject-specific) concerns. Taken together, I am pleased to support the publication on two conditions:
-- providing these comments above be fully addressed and taken into consideration by the authors, as judged by the Editor, and
-- providing the other reviewers have also fully approved the next round(s) of the revised manuscript. I thus do not need to re-review from my side unless the Editors request otherwise.

I wish the authors good luck with their ongoing and future investigations in this area.

Thank you, we appreciate the thoughtfulness and time taken by each reviewer.

Reviewer #4 (Remarks to the Author):

I co-reviewed this manuscript with one of the reviewers who provided the listed reports. This is part of the Nature Communications initiative to facilitate training in peer review and to provide appropriate recognition for Early Career Researchers who co-review manuscripts.

Dear Reviewers,

REVIEWERS' COMMENTS

Reviewer #1 (Remarks to the Author):

1. The authors have significantly enhanced the manuscript's structure, clarity, and statistical rigour. The TAG TKO approach, lipidomics, and PM cholesterol measurements convincingly establish a regulated axis. This axis involves fatty acyl CoA depletion activating ATGL, remodelling PC/PE saturation, and altering plasma membrane cholesterol accessibility.

My primary concern remains the strength of the mechanistic language surrounding ATGL's triacylglycerol-independent DAG remodelling and transacylase activity. Since there's no catalytic dead ATGL rescue or equivalent experiment in this system, I suggest the authors explicitly frame these aspects as a model supported by prior literature and inhibitor data, rather than as a proven mechanism. This clarification should be made in the abstract and discussion, using phrases like "we propose" or "our data are consistent with the model that…" instead of categorical statements.
With this adjustment, I would be happy to recommend publication in Nature Communications.

We appreciate the comment that the mechanistic language could be softened. We have changed the abstract and discussion. In the abstract, we have made this change:

- Previous text: "ATGL activation elevates the abundance of polyunsaturated diacylglycerols through a mechanism that is independent of triacylglycerol hydrolysis."
- New text: "ATGL activation elevates polyunsaturated diacylglycerols even in triacylglycerol-deficient backgrounds, suggesting that ATGL acts independently of triacylglycerol hydrolysis."

We also changed the final sentence of the abstract to reduce the redundancy of this section and to change the claim away from a triacylglyerol-independent mechanism.

- Previous text: “This study reveals a surprising link between fatty acid metabolism and cholesterol homeostasis, demonstrating that ATGL can modify the lipidome through a triacylglycerol-independent mechanism.”
- New text: “This study reveals a surprising link between fatty acid metabolism and cholesterol homeostasis, demonstrating how ATGL-dependent lipid remodeling tunes membrane fluidity to regulate intracellular cholesterol signaling.”

Additionally, in the Discussion we add the phrase “we propose” highlighted in red. We believe the rest of this section already adequately uses terms like “we hypothesize” and “nominates,” which are highlighted in yellow.

“In this study we used epistasis, pharmacology, and fatty-acid rescue experiments to demonstrate that ATGL senses fatty acyl-CoA abundance in cells; we propose that ATGL then acts as a rheostat, converting metabolic information into changes in the physical state of the plasma membrane.

Lipidomics data reveals that ATGL activation elevates polyunsaturated DAG abundance, as well as polyunsaturated PC and PE which we propose are made from DAG precursors. We hypothesize that ATGL generates polyunsaturated DAGs through a transacylation reaction for two reasons. First, ATGL was previously reported to have transacylase activity on DAG both in vitro and in vivo63–66. Secondly, we observed dramatic changes in PM cholesterol accessibility with ACSL1/3-DKO cells and ACSL inhibition. ACSL loss blocks the formation of fatty acyl-CoAs; therefore, the polyunsaturated DAG species must be made through a mechanism that does not require fatty acid charging with CoA. This rules out de novo lipid synthesis, and nominates either elevated desaturase activity or a transacylation reaction. Future work will be needed to determine how ATGL remodels the cellular DAG pool, and the consequences of this activity on cell and organismal biology.”

Reviewer #2 (Remarks to the Author):

This paper has been extensively revised and now argues that cholesterol accessibility at the cell surface in cells is regulated in part by activation of ATGL with this effect preserved even if TAG formation is blocked. Evidence for this includes mass spec showing ATGL activation elevates DAGs which are precursors to membrane phospholipids. They argue that this depends on transacetylase activity by ATGL leading eventually a resulting increase in unsaturated fatty acids in membrane lipids. In their model, this results in an increase of cholesterol accessibility in the PM. Although this is a highly complex system and the dependence on transacetylase activity specifically may be difficult to definitively show in vivo, it is backed by citation of in vivo studies, and also lipidomics which seems to show an increase in polyunsaturated DAGS dependent on ATGL. Overall, the model of the authors is plausible and backed by considerable evidence in the current revision of the paper. My feeling is that while there are still some difficult unsolved questions the paper has been greatly improved and will be of interest to cell biologists and will also prove useful as a basis for future research.

We appreciate the reviewers comments and their enthusiasm for publication at this time.

Reviewer #3 (Remarks to the Author):

In the previous round of revisions, I have included the following final recommendation in my review:
"I believe that responses to the above comments/queries can be overseen by the handling Editor. I further think that the other reviewers have also asked many relevant and important (often subject-specific) concerns. Taken together, I am pleased to support the publication on two conditions:
-- providing these comments above be fully addressed and taken into consideration by the authors, as judged by the Editor, and
-- providing the other reviewers have also fully approved the next round(s) of the revised manuscript. I thus do not need to re-review from my side unless the Editors request otherwise.

I wish the authors good luck with their ongoing and future investigations in this area."

Having gone through the latest version of the manuscript and the other review comments, I do realize that the other referees continue to have non-insignificant concerns. It would be helpful if the authors and the remaining referees could identify the middle ground. Below I provide my personal viewpoints and hope these are of help -

1. First, Nature Communications is a broader readership journal and thus new scientific discoveries of interest and relevance across multiple fields, providing the conclusions and experimental designs, data analyses/interpretations, etc., are rigorous and undertaken with holistic depth and scope should be supported for publications. In this spirit, this manuscript (after several rounds of revisions) do meet such requirements.

2. However, I must also point out that the goal post of the manuscript has shifted in the process of revisions and cutting down some of the initially reported datasets / claims that were not fully substantiated. It appears to me that other reviewers prefer to remain focused on the initial focus on the paper, leading to their limited satisfaction with the revised manuscript. This appears to be a significant stumbling block. I personally believe that the best way to bring this to amicable conclusion is via open communications between both sides mediated by the handling editor and for the authors to clearly cede the limitations of the paper while stressing the key additions brought along through revisions.

We greatly appreciate the reviewer's thoughtful comments during this publication process. We have implemented the suggested changes from Reviewer 1.

Reviewer #4 (Remarks to the Author):

I co-reviewed this manuscript with one of the reviewers who provided the listed reports. This is part of the Nature Communications initiative to facilitate training in peer review and to provide appropriate recognition for Early Career Researchers who co-review manuscripts.
