# Supplementary material for: Fatty acid metabolism controls plasma membrane cholesterol accessibility via ATGL-dependent phospholipid remodeling
Source: Nat Commun. 2026 Oct 14;17:11622. doi: 10.1038/s41467-026-78470-4 (PMC13755437; doi:10.1038/s41467-026-78470-4)
Supplement: Supplementary file 4 [file 41467_2026_78470_MOESM4_ESM.zip › SupplementalData-FlowCytometry-GatingStrategy.pdf]

ACC1 inhibits ATGL to regulate plasma membrane fluidity and cholesterol accessibility

NCOMMS-25-60399-T

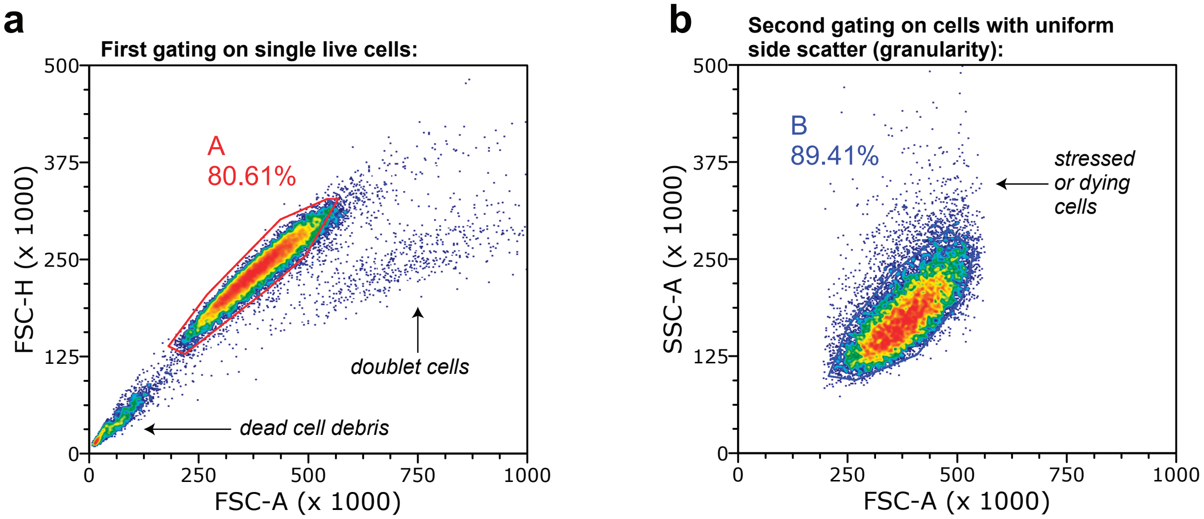

Supplementary File: Flow cytometry gating strategy. All flow cytometry experiments in this manuscript were gated with the following strategy. The example shown above was a typical gating for the HAP1 cell line used in the majority of figures. **a**, First, a density plot showing forward scatter-height (FSC-H) versus forward scatter-area (FSC-A) was made. This plot allowed for gating on single cells and live cell populations (red gate A). **b** The second gated population was performed on the "A" gated cells. A density plot showing side scatter-area (SSC-A) versus forward scatter-area (FSC-A) was made. Cells were then gated to remove cells with high side scatter (high granularity), which removes cells that were sick or dying (blue gate B). Cells from the blue gate "B" were analyzed for fluorescence in the FITC channel to detect mNeonGreen fused to ALOD4 (mNG-ALOD4) or eGFP fused to OlyA (OlyA-EGFP; this is a C-terminal tag) (**Extended data Fig. 3c**). All fluorescence measurments were made on the majority cellular population- not a rare population of cells. As shown above, 80% of cells fell in the "A" gate, and 89% of cells fell in the "B" gate.
