# Supplementary material for: Fatty acid metabolism controls plasma membrane cholesterol accessibility via ATGL-dependent phospholipid remodeling
Source: Nat Commun. 2026 Oct 14;17:11622. doi: 10.1038/s41467-026-78470-4 (PMC13755437; doi:10.1038/s41467-026-78470-4)

This document contains:
1) Uncropped Western Blot scans for all figures and RRID for antibodies used:
- Main figures: 4c and 7d
- Supplementary Figures: 1m, 2c, 4c, 4h, 5a, 5e, 7b, and 7i

2) DNA gel images for all figures:
- Supplementary Figures: 1c, 1d, 1m, 4c, 4h, 5d, 7h, and 8a

-Note, if only part of a Western blot/DNA gel was shown in a figure, a red dotted outline is used to indicate this area.

Uncropped Western blots:

Figure 4c

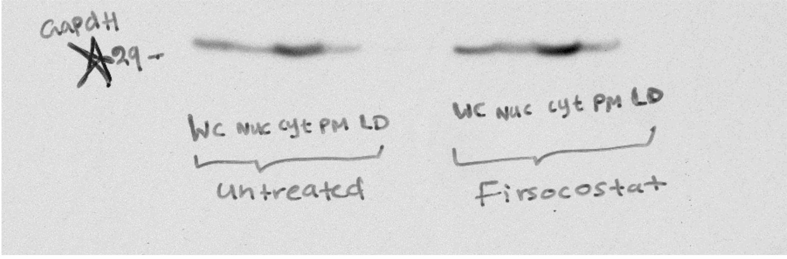

RRID:AB_2107436

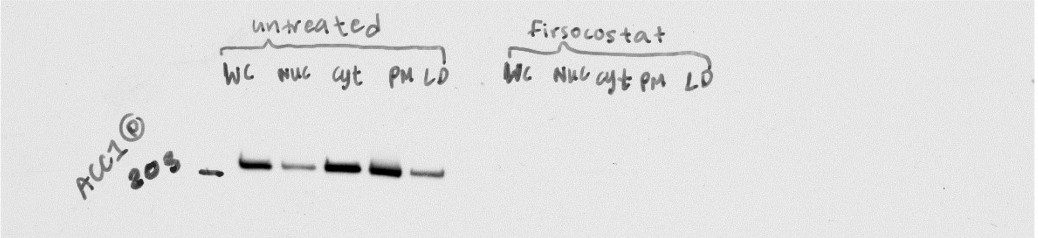

RRID:AB_11156104

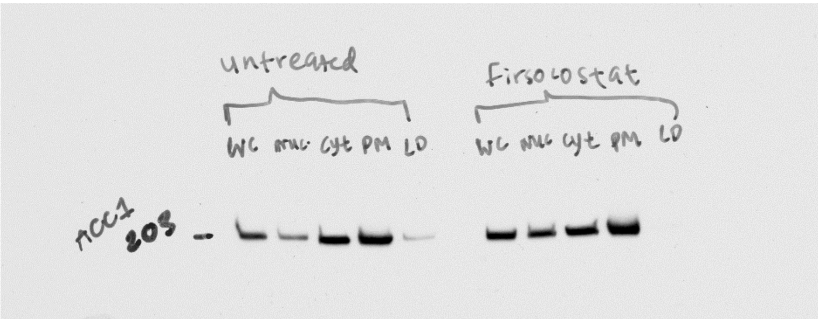

RRID: AB_2882621

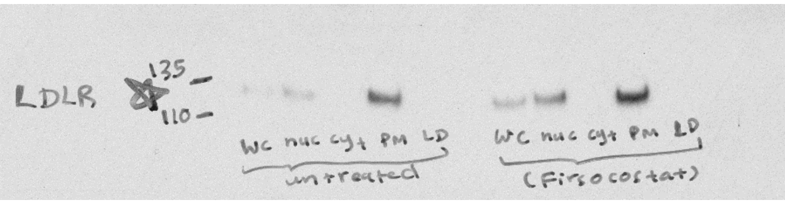

RRID:AB_2281164

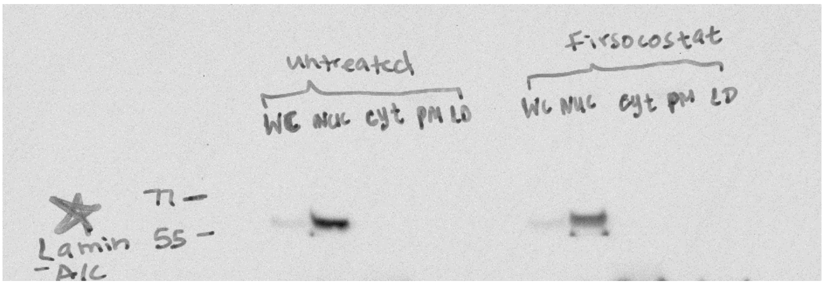

RRID:AB_2296961

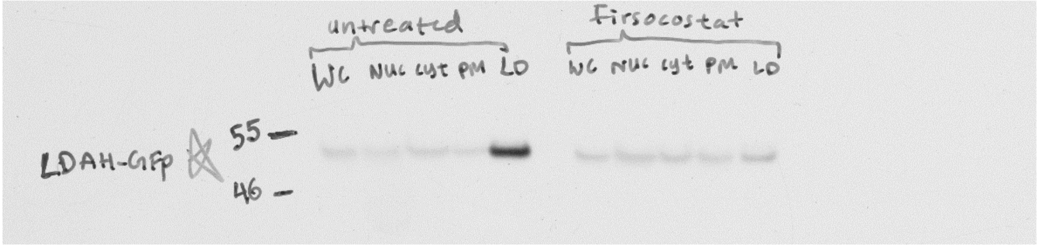

RRID:AB_11182611

Figure 7d

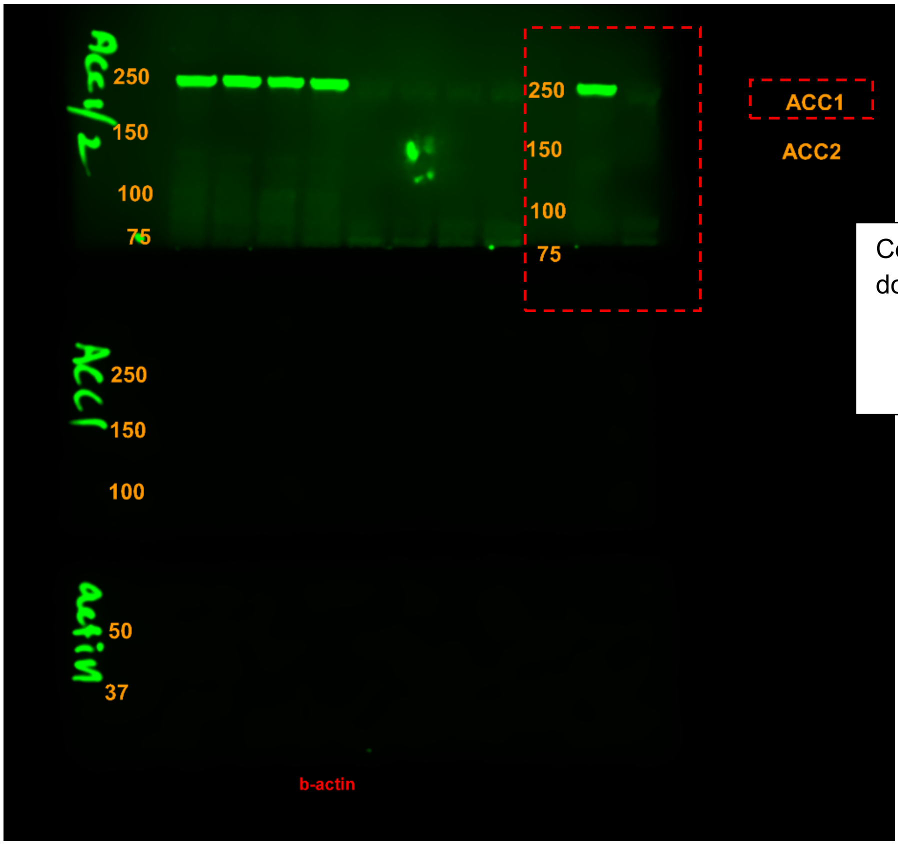

Cell Metab. 2017 Aug 1;26(2):394–406.e6.
doi: 10.1016/j.cmet.2017.07.009

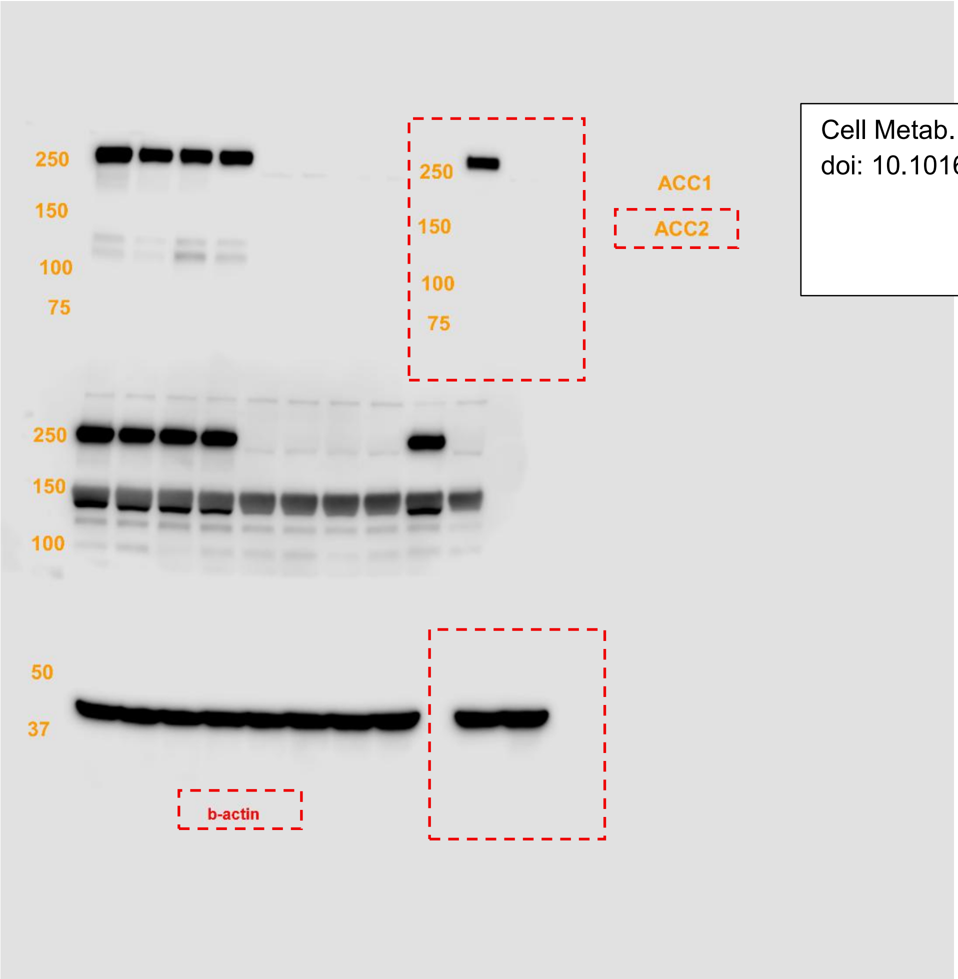

Cell Metab. 2017 Aug 1;26(2):394–406.e6.
doi: 10.1016/j.cmet.2017.07.009

RRID:AB_476744

Supplementary Fig. 1m

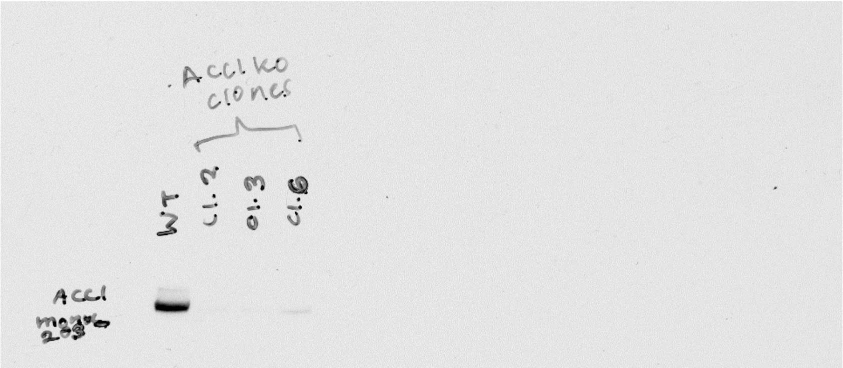

RRID: AB_2882621

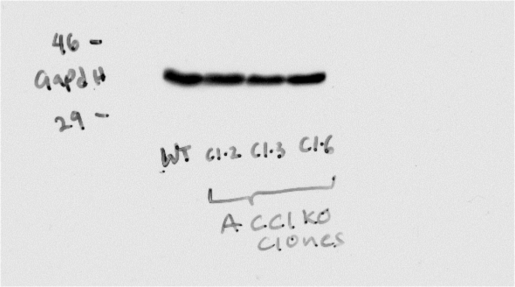

RRID:AB_2107436

Supplementary Fig. 2c

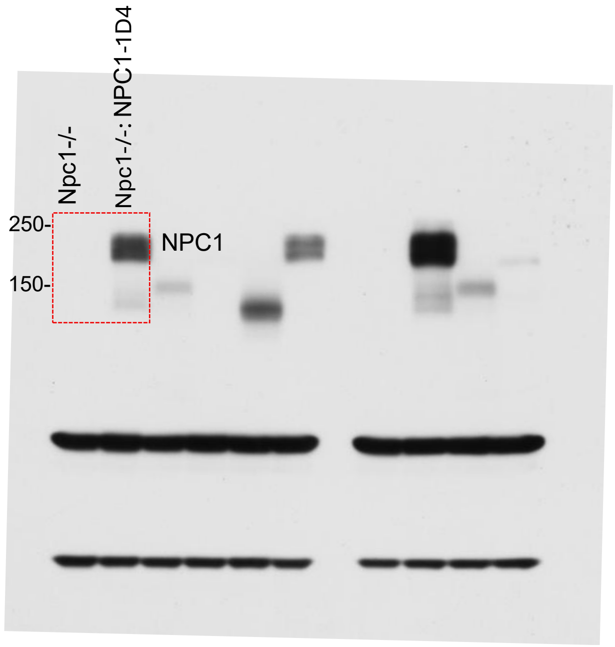

RRID:AB_2152050

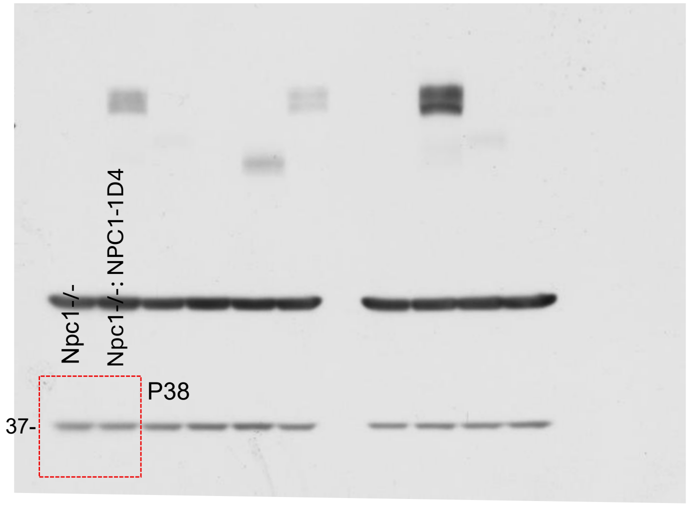

RRID: AB_306166

Supplementary Fig. 4c

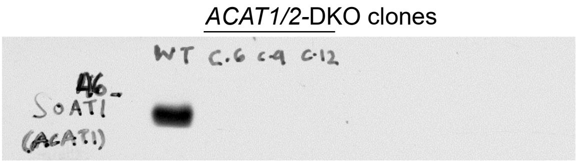

RRID: AB_3750386

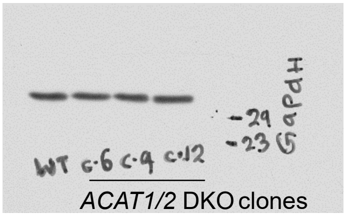

RRID:AB_2107436

Supplementary Fig. 4h

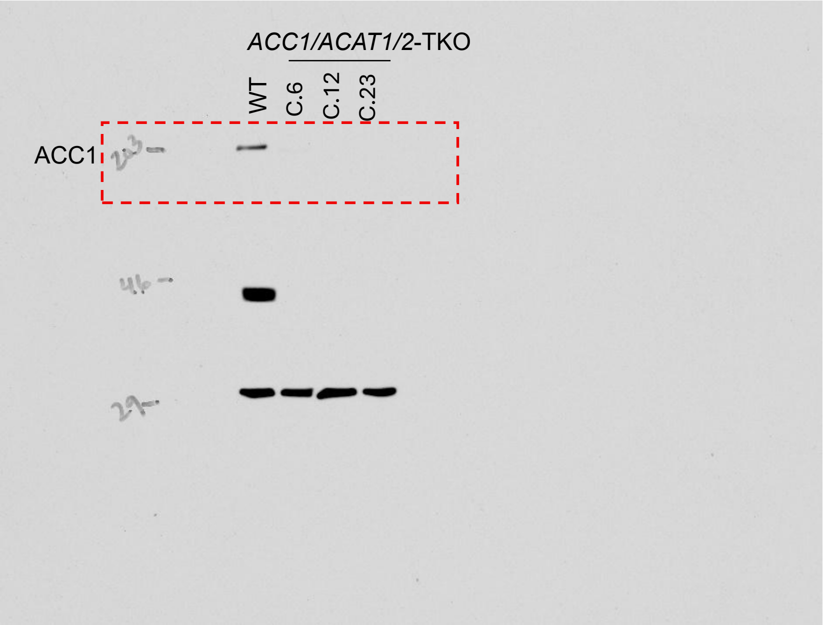

RRID: AB_2882621

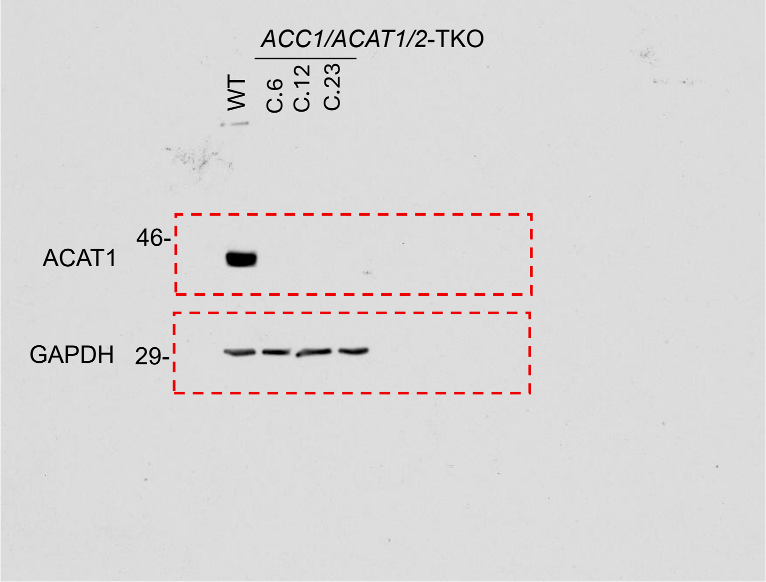

RRID: AB_3750386

RRID:AB_2107436

Supplementary Fig. 5a

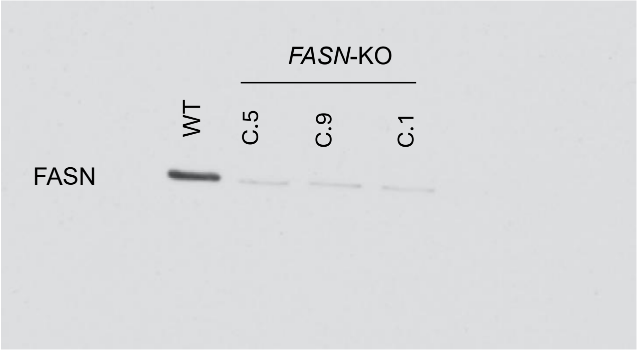

RRID:AB_2100801

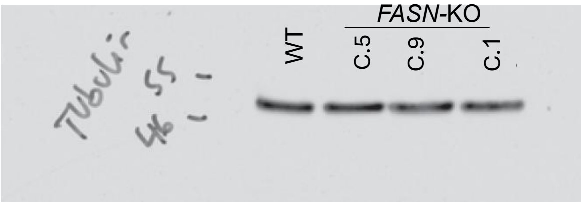

RRID:AB_2210206

Supplementary Fig. 5e

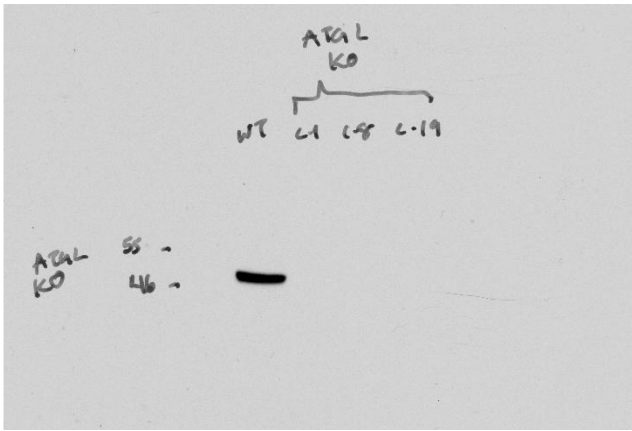

RRID:AB_2888664

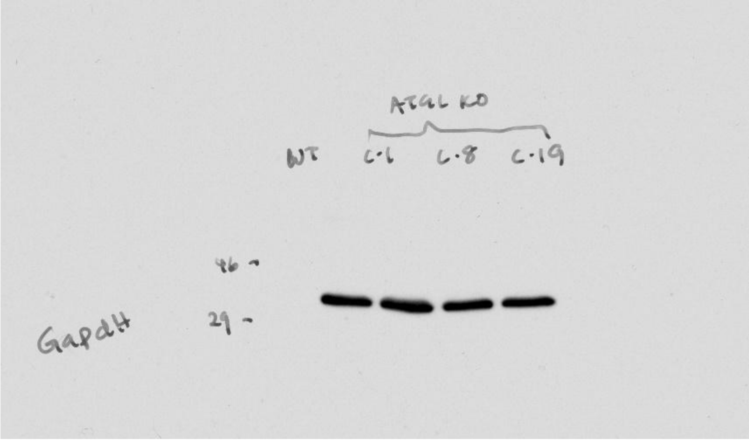

RRID:AB_2107436

Supplementary Figure 7b

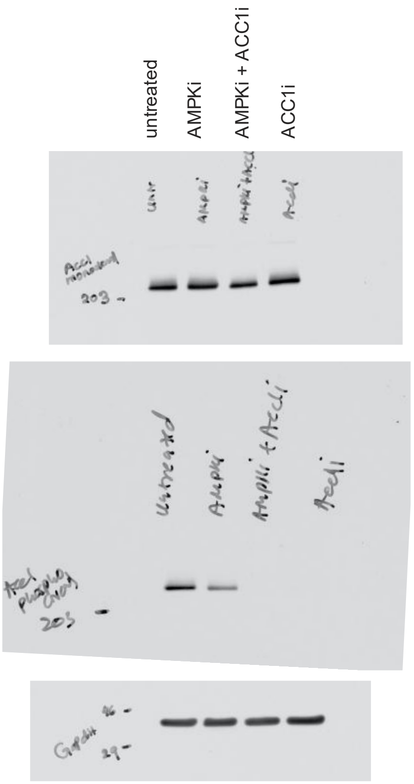

RRID: AB_2882621

RRID:AB_11156104

RRID:AB_2107436

Supplementary Figure 7i

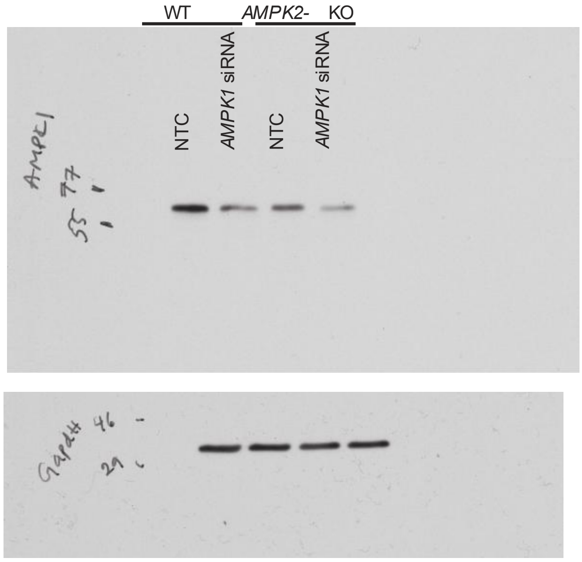

RRID: AB_2881899

RRID:AB_2107436

Uncropped DNA gels:

Supplementary Fig. 1c

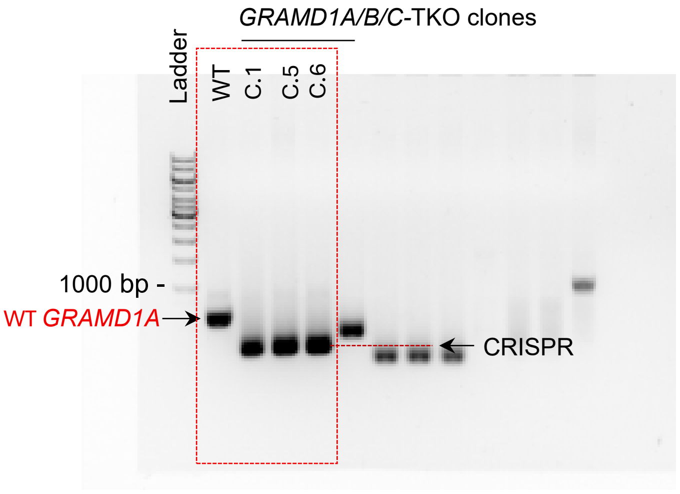

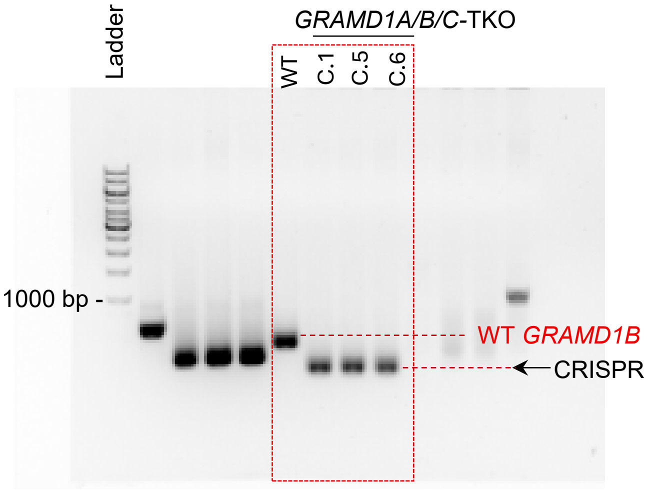

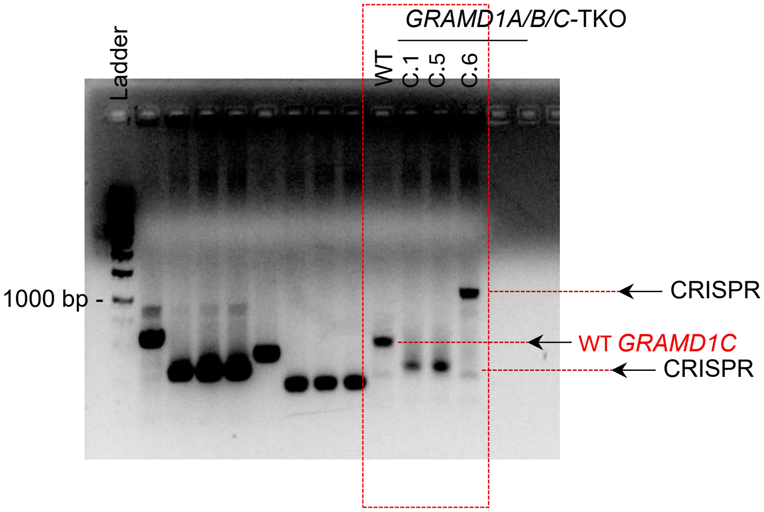

Supplementary Fig. 1d

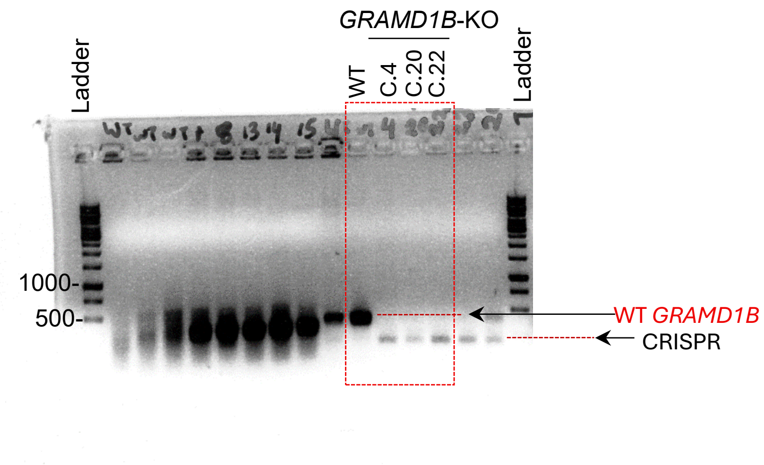

Supplementary Fig. 1m

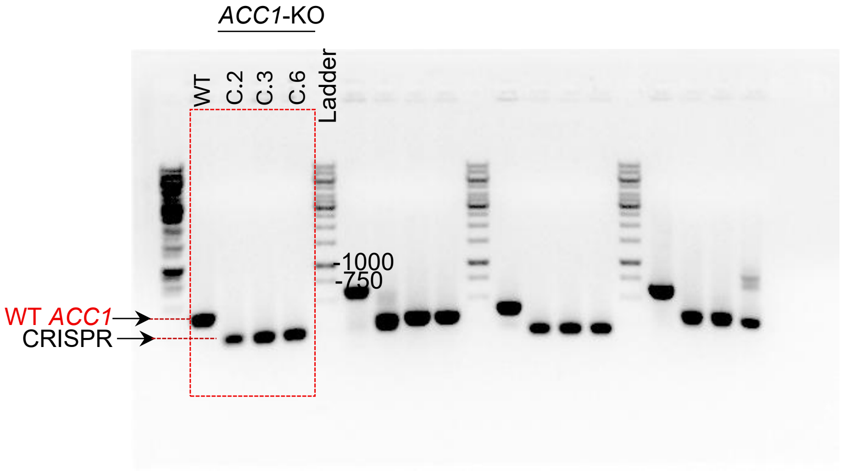

Supplementary Fig. 4c

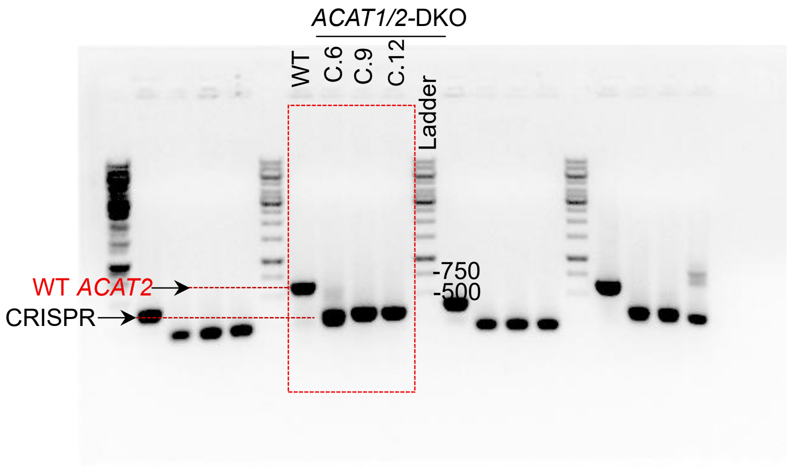

Supplementary Fig. 4h

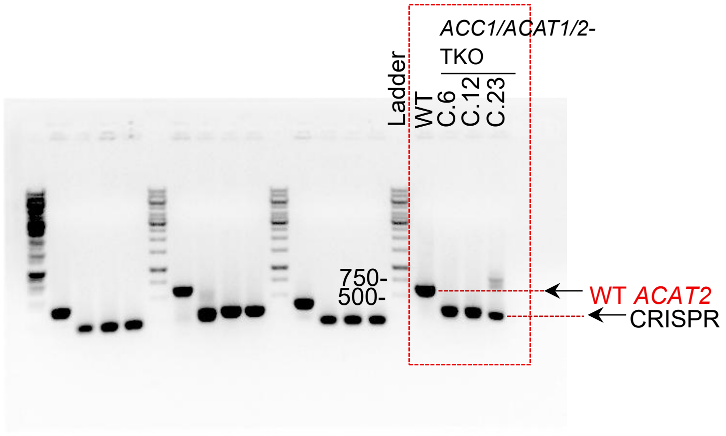

Supplementary Fig. 5d

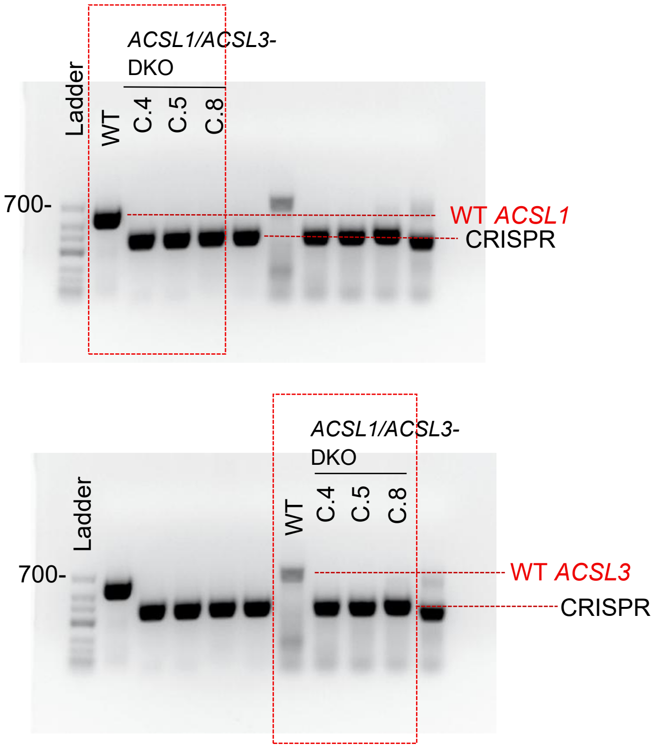

Supplementary Fig. 7h

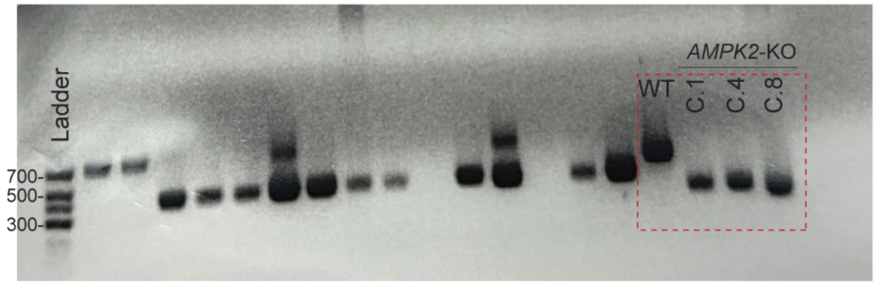

Supplementary Fig. 8a

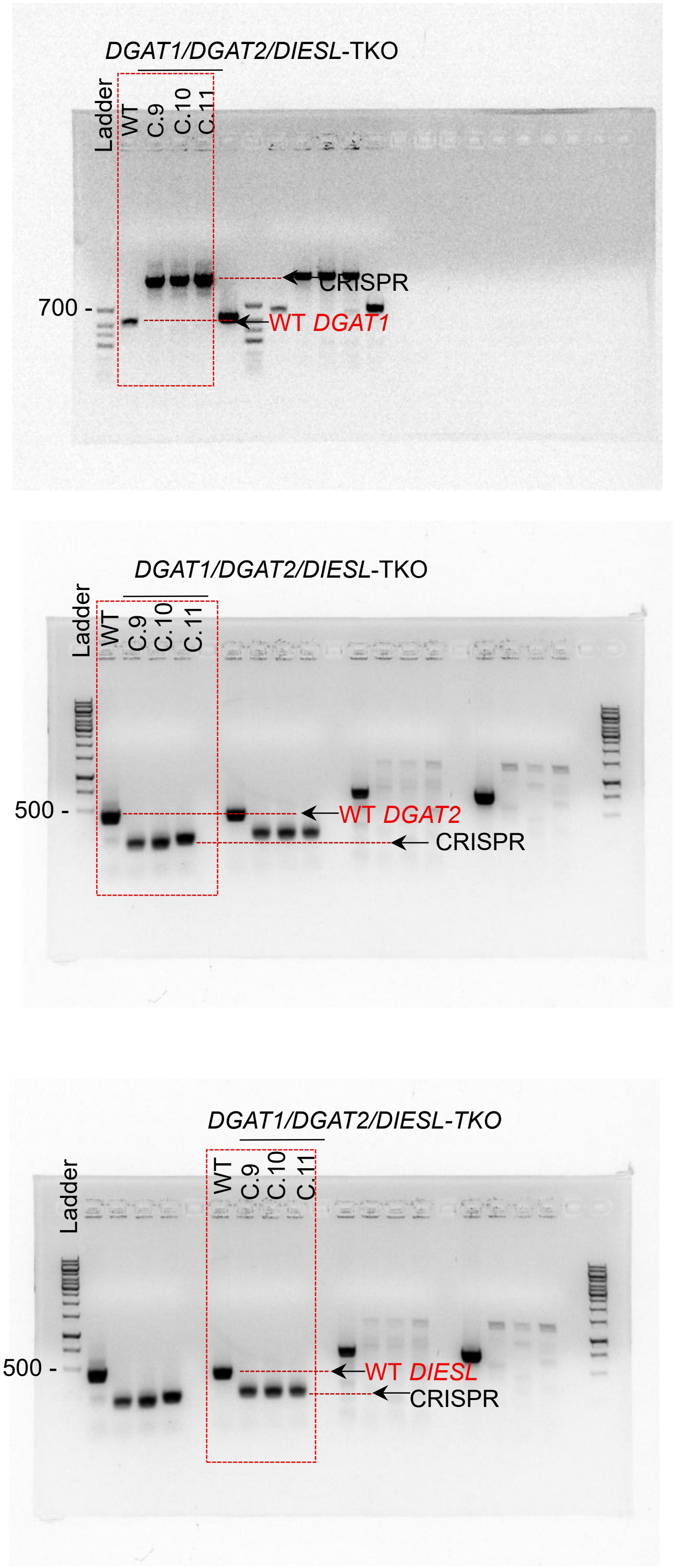

Supplement: Supplementary file 4 [file 41467_2026_78470_MOESM4_ESM.zip › Uncropped-WB-DNAgels-09092026.pdf]
